# Floral Humidity in Flowering Plants: A Preliminary Survey

**DOI:** 10.3389/fpls.2020.00249

**Published:** 2020-03-06

**Authors:** Michael J. M. Harrap, Natalie Hempel de Ibarra, Henry D. Knowles, Heather M. Whitney, Sean A. Rands

**Affiliations:** ^1^School of Biological Sciences, University of Bristol, Bristol, United Kingdom; ^2^Centre for Research in Animal Behaviour, School of Psychology, University of Exeter, Exeter, United Kingdom

**Keywords:** angiosperm, floral displays, floral stomata, humidity, hidden patterns, phylogenetically controlled maximum-likelihood model, pollinator cue, robot arm

## Abstract

The area of space immediately around the floral display is likely to have an increased level of humidity relative to the environment around it, due to both nectar evaporation and floral transpiration. This increased level of floral humidity could act as a close-distance cue for pollinators or influence thermoregulation, pollen viability and infection of flowers by fungal pathogens. However, with a few exceptions, not much is known about the patterns of floral humidity in flowering plants or the physiological traits that result in its generation. We conducted a survey of 42 radially symmetrical flower species (representing 21 widely spread families) under controlled conditions. Humidity was measured using a novel robot arm technique that allowed us to take measurements along transects across and above the floral surface. The intensity of floral humidity was found to vary between different flower species. Thirty of the species we surveyed presented levels of humidity exceeding a control comparable to background humidity levels, while twelve species did not. Patterns of floral humidity also differed across species. Nevertheless, floral humidity tended to be highest near the center of the flower, and decreased logarithmically with increasing distance above the flower, normally declining to background levels within 30 mm. It remains unclear how physiological traits influence the diversity of floral humidity discovered in this survey, but floral shape seems to also influence floral humidity. These results demonstrate that floral humidity may occur in a wide range of species and that there might be greater level of diversity and complexity in this floral trait than previously known.

## Introduction

Some floral traits are difficult for humans to identify but nevertheless influence floral function, or are detectable to floral visitors (Baker, 1977; Clarke et al., 2013; Harrap et al., 2017, 2019; Lawson et al., 2018; Patiño and Grace, 2002; Rands et al., 2011; Whitney et al., 2016). Floral humidity is another such trait and is characterized by an increase in humidity relative to background levels in the space around the flower. It is likely to arise from evaporation of liquid nectar and transpirational water loss through the petal. However, so far it has not yet been systematically characterized.

Humidity increases were found in proximity to *Digitalis*, *Echium*, and *Helleborus* flowers (Corbet et al., 1979a, b). More recently, detailed insights were gained from the study of the evening primrose *Oenothera caespitosa* (von Arx et al., 2012), where it was found that in the space around individual flowers, humidity is approximately 4% higher than the background ambient level over a short distance range. Interestingly, this range corresponds to the hovering distance of their long-tongued hawkmoth pollinators (von Arx et al., 2012). Insects are known to be very sensitive to humidity (Enjin, 2017). Taken together, there is good evidence to suggest floral humidity could be suitable as a short-distance cue in decision-making and spatial orientation of insect pollinators. Floral humidity may have other important impacts on floral fitness that have not been explored. Pollen viability and ability to germinate often depends on pollen water content, which in turn is influenced by moisture and humidity conditions. This is particularly the case in species whose pollen normally has higher water content (Nepi et al., 2001; Prokop et al., 2019). Pollen water content that is too high can cause the pollen to burst under turgor pressure (Jacquemart, 1996; Von Hase et al., 2006), whilst water content that is too low causes dehydration and desiccation of the pollen (Nepi and Pacini, 1993; Nepi et al., 2001); both cases reduce pollen viability. Floral humidity may affect pollen water content and viability after anther dehiscence, perhaps even working as a mechanism to maintain pollen at suitable conditions. Transpiration and nectar evaporation impact floral temperature, perhaps acting as a cooling mechanism (Baker, 1977; Patiño and Grace, 2002; Seymour and Schultze-Motel, 1998). In this manner, the presence of elevated floral humidity may indicate temperature control measures of the flower (Shrestha et al., 2018). Control of floral temperature is important as it influences floral metabolism, pollen and ovule viability (van der Kooi et al., 2019) and pollinator responses to the floral display (Dyer et al., 2006; Rands and Whitney, 2008; Whitney et al., 2008; Harrap et al., 2017). Furthermore, environments with highly elevated humidity enhance the growth of fungal pathogens (e.g., Williamson et al., 1995, 2007; Keller et al., 2003). It is possible that floral humidity levels may likewise influence floral infection.

It is likely that a wide range of flower species produce floral humidity, because liquid nectar is abundant in angiosperms (Percival, 1961; Brandenburg et al., 2009) and transpiration is a ubiquitous plant process (Baker, 1977; Gates, 1968; Jarvis and McNaughton, 1986; Morgan, 1984; Liang et al., 2010). It has been reported that flowers that were preferred by flies in Indian alpine environments had higher humidity levels than flowers that were visited less often (Nordström et al., 2017). However, such surveys have not determined how humidity changes about individual flowers or contrasts with background levels, as compared to the study conducted by von Arx et al. (2012). It remains unclear whether floral humidity occurs more widely and thus may function frequently as a floral cue and provide sensory information that is useful for pollinators. Likewise, the links between the effects of floral humidity on pollen viability, flower temperature regulation and fungal infection remain uncertain.

Humidity intensity may vary amongst different species with different levels of transpiration and nectar production. Further differences in physiology, flower size and morphology may also influence how intensity peaks and how the gradients of floral humidity are distributed in the space surrounding an individual flower, creating structural differences in floral humidity. The location and orientation of nectaries may influence where evaporated vapor accumulates in the flower. In the case of *O. caespitosa* the greatest humidity within the floral headspace was found above the flower corolla, presumably due to the tubular shape of its corolla with nectaries located at the very bottom of the flower (von Arx et al., 2012). Humidity decreased with increasing distance from the flower (Corbet et al., 1979a, b; von Arx et al., 2012). Draining *O. caespitosa* flowers of nectar, or plugging their corolla tube, reduced the intensity of this floral humidity difference, but not completely (von Arx et al., 2012). This strongly suggests that transpirational water loss through the petal contributes to flower humidity. Differences in permeability across the petal cuticle, as well as the location and density of petal stomata (Baker, 1977; Huang et al., 2018) may similarly alter where transpirational water loss occurs.

To evaluate how floral humidity varies across different species, it is important to conduct measurements under controlled conditions. It is possible to sample flowers in the field using a stationary probe, which can detect differences in floral humidity compared to the background. However, these methods carry a risk of underestimating the humidity generated by the flower due to inaccurate positioning of the measuring probes. Furthermore, as humidity declines rapidly over short distances (Corbet et al., 1979a, b; von Arx et al., 2012) it is very difficult to control for other near sources of humidity. Sampling procedures measuring humidity at many points within the flower headspace of isolated flowers, similar to the humidity transects carried out by von Arx et al. (2012), are therefore required for investigating how flower humidity differs across species and changes with distance from the flower.

Understanding the ways that different flower species’ floral humidity varies in intensity and structure may reveal features that potentially could convey sensory information to flower visitors or have implications for pollen viability and floral temperature control, or susceptibility of different species to fungal disease. In the present study, we analyzed the humidity in the headspaces of individual flowers from 42 species, including resampling *O. caespitosa*, using a humidity probe supported by a robotic arm carrying out a similar transect method to that described by von Arx et al. (2012).

## Materials and Methods

### Robot Sampling Setup

All floral humidity measurements were taken within a 6.11 m × 3.67 m room within the Bristol University Life Sciences Building (51°45′N 2°60′W) that had temperature and airflow controls for maintaining a constant background temperature and humidity. A 3.72 m × 3.67 m section of this room, hereafter referred to as the ‘sampling zone,’ was separated off within a 2 m high wall of 10 mm thick clear polycarbonate. A six-axis articulated *Staubli* RX 160 robot arm (Pfäffikon, Switzerland) was mounted in the center of the sampling zone. A scale floorplan for this room is given in Supplementary Figure S1 in Supplementary File 1.

The background temperature was kept constant at 23.01 ± 1.38°C (Mean, SD), as the amount of water vapor indicated by a relative humidity value varies depending on air temperature (Tichy and Kallina, 2014). For example, an increase in air temperature of 10°C approximately doubles the amount of water vapor indicated by the same relative humidity value. Thus, the constant lab temperature allowed us to compare relative humidity values across all flower measurements. The relative humidity of the room (henceforth referred to as background humidity) during sampling was 49.1 ± 12.3%. Background temperature and humidity were checked regularly during flower humidity measurements.

**FIGURE 1:**
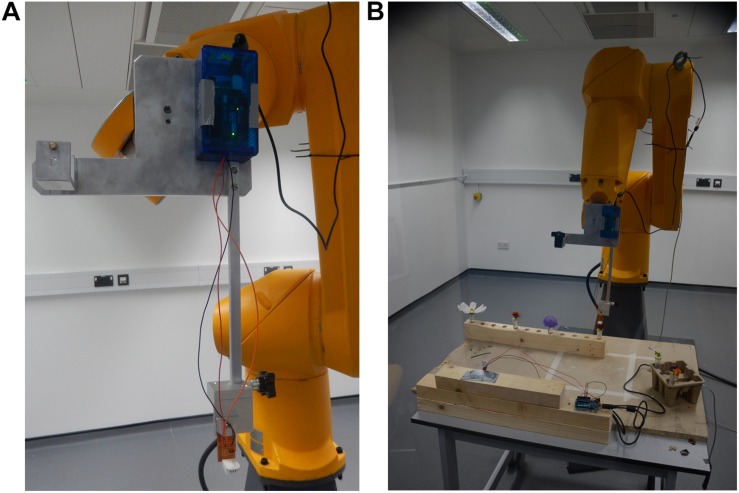
The 6-axis robot arm used for floral humidity sampling. **(A)** The humidity sensing tool mounted onto the robot arm. An adapter mount (a modified Manfrotto 625 adapter, Leicestershire, United Kingdom) is attached to the robot flange (the tool mounting surface at the end of the arm) and a purpose-built sensor tool fitted. This tool consists of a metal plate attached to the adapter mount with a 30 cm long steel bar running parallel to the flange screwed onto it. The DHT-22 humidity sensing probe can be seen at the bottom of the panel attached to the end of the 30 cm steel bar, the probe’s microcontroller at the top of the panel on the metal plate mounting. The position the arm is seen in here is the ‘safe’ position the robot returns to throughout sampling. **(B)** The arm during transect central point teaching. Note flower positions and location of the other background probe on the table in front of the arm.

All humidity measurements were taken using DHT-22 humidity probes (Aosong Electronics, Huangpu, China) attached to an *Arduino UNO* microcontroller (Adafruit Industries, New York, NY, United States). The *Arduino* transmitted the measurements via a USB port to a PC located outside the sampling zone (behind the polycarbonate screen). Two probes were used in measurements: a ‘background probe,’ which measured background humidity, and a ‘focal probe,’ which measured the humidity in the headspace of the object being sampled. The focal probe was mounted on the robot as detailed in Figure 1.

A 75 cm × 90 cm × 74 cm (width × length × height) wooden table was placed in the sampling zone. A rack for 24 cm^3^ horticultural tubes was fixed along one length of the table (Supplementary Figure S1) where horticultural tubes containing flowers could be placed. A 17 cm high mount was attached on the other side of the table for the background probe, with its microcontroller sitting on the table itself. The background probe was located at a sufficiently long distance (44.5–54.5 cm) from any hole in the rack (Figure 1 and Supplementary Figure S1) in order to prevent exposure to flower humidity.

The two microcomputers registering the values from the focal and background probes were at a distance of 34.5 and 32.0 cm from their respective probes. They did not heat up significantly or possess any elements that might have generated air turbulence, whilst all elements relating to the control of the robot arm and the data-storing PC were located behind the polycarbonate screen (Supplementary Figure S1). This meant that both of the humidity probes were unaffected by heat or air turbulence generated by any equipment in the room. Furthermore, installing a 30 cm bar on the measurement tool (Figure 1) ensured that the probe was held far from any moving part of the robot arm. The arm moved slowly during sampling (at a maximum of 3% of nominal speed; estimated to be well below 200 mm s^–1^) and paused before starting to record measurements to allow any minute air disturbances to settle. Overall, the robotic arm was also unlikely to change the intensity and gradients of humidity produced by the sampled flowers.

### Preparation of Flowers

To separate floral humidity from other sources of humidity on the plant, flowers were cut and placed in horticulture tubes prior to the start of the measurements. Furthermore, cutting allowed us to control the orientation of flowers during sampling (Figure 1B). Flowers were either collected from University of Bristol gardens (Royal Fort Garden, the School of Chemistry gardens and Woodland Road gardens, all within 51°45′N 2°60′W), the University of Bristol Botanical Garden (51°47′N 2°63′W), or grown within the Bristol Life Sciences Building glasshouse (51°45′N 2°60′W). Flowers were cut on the stem so no leaf remained on the cutting. Sepals were retained. Immediately after cutting off the plant, the stems were stuck through a hole in the cap of a water-filled 24 cm^3^ plastic horticulture tube. Horticulture tubes were filled to a point 2 cm from the tubes’ lid to provide the flowers unrestricted access to water. Cut flowers were then taken into the lab in these tubes, and measurements started quickly after arrival. If flowers were grown outdoors, care was taken to collect them in dry conditions, so that no standing water from condensation or rain could influence the measurements. Flowers were only used if they were fully open and did not show signs of age, disease or damage.

Table 1 gives details of the number of replicates and growth conditions of 42 sampled flower species (this includes three horticultural varieties which were easily available). We aimed to sample six flowers from different plants, however, this was not always possible due to limited availability of some species (see Table 1). In most species, individual flowers were sampled, but in species with small or compound flowers we sampled inflorescences, for example in flowers of the family Asteraceae (Table 1). Either three or four flowers were sampled each day. The species of flowers sampled each day was mixed. Most often on a single day four individuals of different species would be sampled. However, this varied dependent on available flowers of each species, and shorter flowering periods of some species meant they were prioritized on some days. The full list of dates of individual flower sampling, ordered by species, are found in Appendix S1 in Supplementary File 1.

**TABLE 1:** The plant species sampled with the floral humidity headspace methods.

**Species**	**Order**	**Family**	**Floral unit**	***n***	**Growth conditions**	**Growth location**
*Allium ursinum* L.	Asparagales	Alliaceae	Flower	4 (4)	Outside	Gardens
*Tulbaghia violacea* Harv.	Asparagales	Alliaceae	Flower	6 (6)	Outside	Botanics
*Achillea millefolium* L.	Asterales	Asteraceae	Umbel inflorescence	6 (6)	Outside	Gardens
*Bellis perennis* L.	Asterales	Asteraceae	Compound inflorescence	6 (6)	Outside	Gardens
*Coreopsis* sp. L.	Asterales	Asteraceae	Compound inflorescence	6 (2)	Inside	Glasshouse
*Cosmos bipinnatus* Cav.	Asterales	Asteraceae	Compound inflorescence	6 (2)	Inside	Glasshouse
*Cyanus montanus* Hill	Asterales	Asteraceae	Compound inflorescence	6 (6)	Outside	Gardens
*Cyanus segetum* Hill	Asterales	Asteraceae	Compound inflorescence	6 (6)	Outside	Gardens
*Leucanthemum vulgare* Lam.	Asterales	Asteraceae	Compound inflorescence	5 (5)	Outside	Gardens
*Osteospermum* sp. L.	Asterales	Asteraceae	Compound inflorescence	6 (2)	Inside	Glasshouse
*Rudbeckia hirta* L.	Asterales	Asteraceae	Compound inflorescence	6 (3)	Outside	Gardens
*Taraxacum* agg. F.H. Wigg	Asterales	Asteraceae	Compound inflorescence	6 (6)	Outside	Gardens
*Xerochrysum bracteatum* (Vent.) Tzvelev	Asterales	Asteraceae	Compound inflorescence	6 (3)	Inside	Glasshouse
*Campanula* sp. L.	Asterales	Campanulaceae	Flower	6 (2)	Inside	Glasshouse
*Nepenthes* sp. L.	Caryophyllales	Nepenthaceae	Flower	6 (4)	Inside	Glasshouse
*Scabiosa* sp. L.	Dipsacales	Dipsacaceae	Compound inflorescence	6 (2)	Inside	Glasshouse
*Trifolium pratense* L.	Fabales	Fabaceae	Umbel inflorescence	6 (6)	Outside	Gardens
*Vinca herbacea* Waldst. & Kit.	Gentianales	Apocynaceae	Flower	6 (6)	Outside	Gardens
*Geranium* ‘Roxanne’	Geraniales	Geraniaceae	Flower	6 (6)	Outside	Gardens
*Geranium robertianum* L.	Geraniales	Geraniaceae	Flower	6 (6)	Outside	Gardens
*Geranium sanguineum* L.	Geraniales	Geraniaceae	Flower	6 (6)	Outside	Gardens
*Lavandula angustifolia* Mill.	Lamiales	Lamiaceae	Racemose inflorescence	6 (6)	Outside	Gardens
*Lantana* sp. L.	Lamiales	Verbenaceae	Umbel inflorescence	6 (4)	Inside	Glasshouse
*Lilium* sp. L.	Liliales	Liliaceae	Flower	6 (6)	Inside	Glasshouse
*Euphorbia milii* Des Moul.	Malpighiales	Euphorbiaceae	Compound inflorescence	6 (3)	Inside	Glasshouse
*Linum grandiflorum* Desf.	Malpighiales	Linaceae	Flower	6 (6)	Outside	Gardens
*Linum usitatissimum* L.	Malpighiales	Linaceae	Flower	6 (6)	Outside	Botanics
*Cistus ’*greyswood pink’	Malvales	Cistaceae	Flower	6 (6)	Outside	Gardens
*Abutilon* × *milleri* hort.	Malvales	Malvaceae	Flower	6 (6)	Outside	Botanics
*Epilobium hirsutum* L.	Myrtales	Onagraceae	Flower	6 (6)	Outside	Gardens
*Fuchsia* sp. L.	Myrtales	Onagraceae	Flower	6 (6)	Outside	Gardens
*Oenothera caespitosa* Gillies ex Hook. & Arn.	Myrtales	Onagraceae	Flower	6 (6)	Inside	Glasshouse
*Eschscholzia californica* Cham.	Ranunculales	Papaveraceae	Flower	6 (6)	Outside	Gardens
*Papaver cambricum* L.	Ranunculales	Papaveraceae	Flower	6 (6)	Outside	Gardens
*Papaver rhoeas* L.	Ranunculales	Papaveraceae	Flower	6 (6)	Outside	Gardens
*Clematis chinensis* Osbeck	Ranunculales	Ranunculaceae	Flower	4 (4)	Outside	Botanics
*Ranunculus acris* L.	Ranunculales	Ranunculaceae	Flower	6 (6)	Outside	Gardens
*Ranunculus lingua* L.	Ranunculales	Ranunculaceae	Flower	6 (6)	Outside	Gardens
*Potentilla* sp. L.	Rosales	Rosaceae	Flower	6 (6)	Outside	Gardens
*Calystegia silvatica* (Kit.) Griseb.	Solanales	Convolvulaceae	Flower	6 (6)	Outside	Gardens
*Convolvulus sabatius* Viv.	Solanales	Convolvulaceae	Flower	6 (6)	Outside	Botanics
*Nicotiana tabacum* L.	Solanales	Solanaceae	Flower	6 (6)	Inside	Glasshouse

*The level of the floral unit sampled is indicated, either a single flower or an inflorescence. Plants came from either the Bristol University Glasshouse (“Glasshouse”), Bristol University Gardens (“Gardens”), or Bristol University Botanic Gardens (“Botanics”), and n indicates the number of flowers sampled, with the number of individual plants these came from given in parentheses.*

**FIGURE 2:**
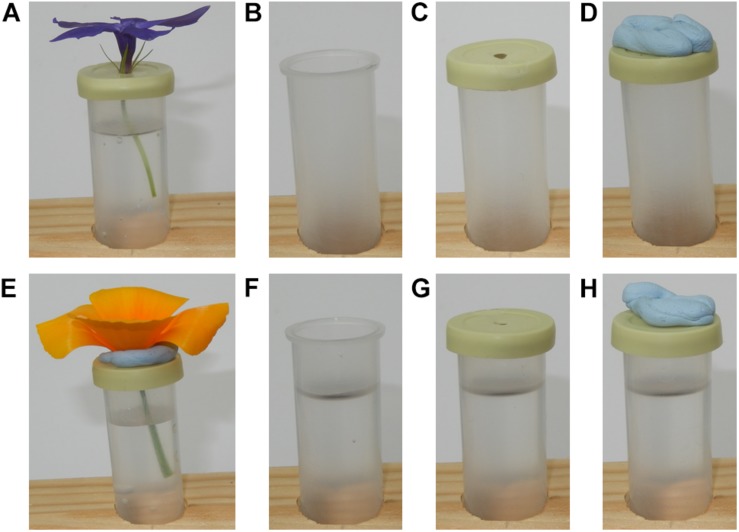
Flowers and control samples as placed under the robot during sampling. **(A)** a *Vinca herbacea* flower. **(B)** The T control; an empty horticulture tube. **(C)** The TL control; an empty horticultural tube with the tubes lid on. **(D)** The TLP control; an empty horticulture tube with a lid and mouldable putty applied. **(E)** An *Eschscholzia californica* flower, note the putty used to keep the flower upright. **(F)** The TW control; a horticultural tube filled to the same level as when flowers were sampled. **(G)** The TWL control; a water filled horticulture tube with its lid applied. **(H)** The TWLP control; a water filled tube with the lid and mouldable putty applied.

Once flowers were taken into the lab, they were placed in the rack on the table in the sampling zone, and spaced at least 15.5 cm apart from one another. Flowers were orientated so that they faced vertically upward: when needed, support was provided to a flower using mouldable putty (*blu tack*: Bostik, Paris, France) stuck to the tube lid (see Figures 2A,E).

### Control Samples

Six different controls were implemented: T, an empty horticultural tube (Figure 2B); TL, an empty tube with a fitted lid (Figure 2C); TLP, an empty tube with fitted lid and putty applied (Figure 2D); TW, a tube filled with water to the same level as those that had contained flowers during sampling (Figure 2F); TWL, a tube filled with water and a fitted lid (Figure 2G); TWLP, a tube filled with water with a fitted lid and putty applied (Figure 2H). Six individual tubes set up for each control were sampled under the robot, except for the TL control where seven individual tubes were sampled. All controls were sampled in exactly the same way as flowers (see below).

These controls were designed to assess the extent of differences in humidity that may occur between the focal and background probe due to influences extraneous to the flower. The flowers sampled were placed in a water filled horticultural tube covered by a lid with a 3 mm diameter hole. Although this hole in the lid was largely blocked by the flower, it was possible that evaporation of the water within the tube could have generated extraneous humidity, consequently we have the TWL and TWLP controls which represent these extraneous humidity sources when the flower is absent. It was also necessary to have a positive control to assess how much humidity and a water source unrestricted by the lid and flower would produce, the TW control. Furthermore, humidity across rooms is rarely completely even, due to how air mixes within a room (Schellenberg, 2002; Lake et al., 2003). Therefore, any difference in humidity between the focal and background probes’ readings due to their different positions in the room had to be measured, this could be assessed using the T and TL controls. Lastly, the putty applied to the flowers could potentially also be a source of humidity extraneous to the flower this could be evaluated by comparing the TL and TLP controls.

### Automated Transect Movements of the Focal Humidity Probe

Humidity measurements commenced no later than 1 h after flowers were cut. The mean start time of a daily sampling trial (taken as the time humidity measurements began after the robot had been activated) was 11:01 h, ranging from 9:10 h to 14:00 h. A full list of the times of the first measurements taken on each individual flower and control are given in Appendix S1 and S2, respectively (see Supplementary File 1). We therefore assume that the flowers were in a late morning or noon state with regards to the plant’s daily cycles such as transpiration, stomatal opening, nectar secretion or floral metabolism.

All humidity measurements were carried out autonomously by the robot after moving the focal probe to predefined measurement positions in a predefined order, creating a transect-like sequence of humidity measurements. As a first step on a sampling day, the measurement positions of the focal probe had to be adjusted using manual controls, in order to account for different sizes and shapes of flowers. Thus, once flowers were ready for sampling, the focal probe was maneuvered to a ‘transect central point’ above each flower – the point above the center of the flower, 5 mm higher than the flower’s highest point. The transect central point for each flower and a single point less than 2.5 cm of the background probe were stored in the robot’s memory. Once a point is stored in the robot’s memory, the robot can return the focal probe to this position, and positions relative to them, in a predefined sequence.

**FIGURE 3:**
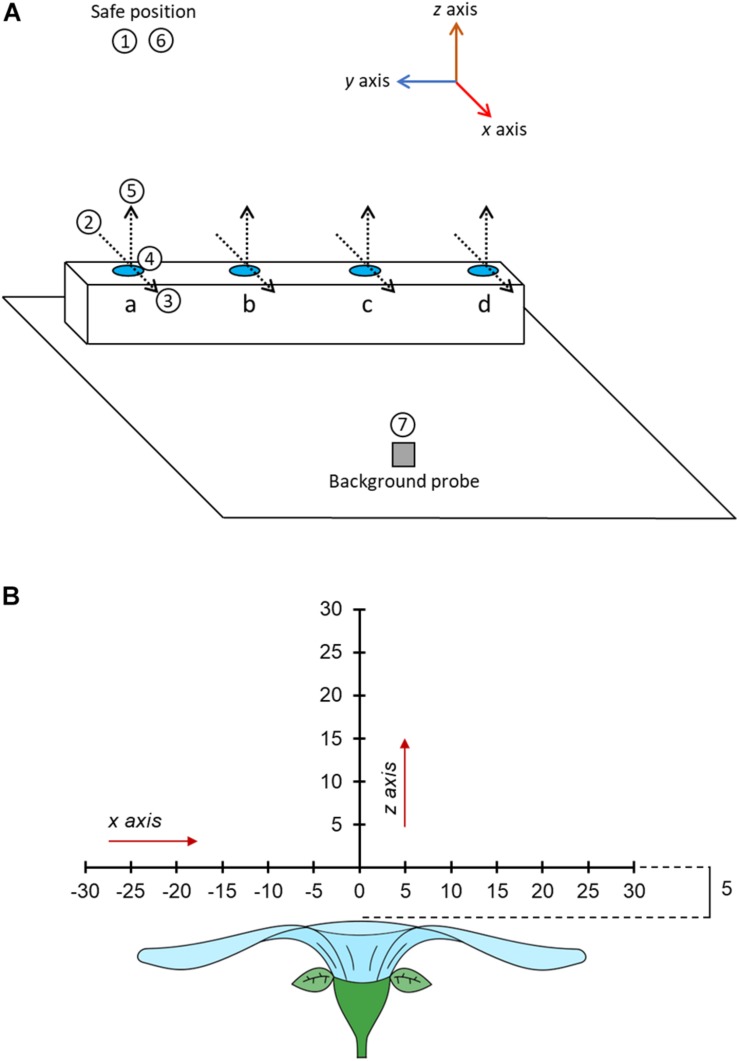
**(A)** A three-dimensional schematic of the table in the sampling zone and the motions of the robot during sampling. Positions of flowers (or controls) to be measured are indicated by blue circles at points a, b, c, and d. The position of the background probe on the table is indicated by the gray square. The position and orientation of humidity transects of each flower (where floral humidity is actually measured) are indicated by dotted lines, arrowheads of these transect lines indicate the direction of probe motion during these transects. The motion of the focal probe while sampling the flower at point a are given in the numbered white circles. 1, The probe begins at the safe position. 2, the probe is moved to a point –30 mm offset in the *x* axis from the transect central point of flower a. 3, the probe carried out the *x* axis transect moving horizontally across the flower to a position 30 mm offset in the *x* axis from the transect central point of flower a, measuring humidity at 5 mm intervals. 4, the probe moves to a position 5 mm offset in the *z* axis from the transect central point. 5, the probe carried out the *z* axis transect moving vertically upward to a position 30 mm offset in the *z* axis from the transect central point of flower a, measuring humidity at 5 mm intervals. 6, the probe moves to the safe position. 7, the probe moves to a position next to the background probe and carries out a probe calibration measurement. The robot would then carry out the same steps for flowers in position b, c, and d, in these instances movements 2–5 are *trans*-located to the other positions. This whole sequence is then repeated four times. Note that the sampling order of flowers will be randomly allocated. The axis co-ordination of the robot is indicated in the image, note how *x* and *z* humidity transects align with the axis orientation of the robot. **(B)** The spatial layout of the humidity headspace sampled above the flower in our transects. The flower is viewed in cross section sideways on. All offset measurements are given in millimeters. Each sampling point is marked along the transect a dash and the distance along that transect relative to the transect central point (where *x* = 0 and *z* = 0).

The sequence order in which individual flowers were sampled on each day was selected via a randomization algorithm within the arm’s software. The robot began each sampling sequence with the focal probe to a ‘safe’ position 50 cm above the table (Figure 1A). In the robot co-ordinate system, *x* axis movements are horizontal, moving toward and away from the robot (negative and positive, respectively); *z* axis movements are up and down (positive and negative, respectively). The axes of the robot relative to the room and the table in the sampling zone are indicated in Supplementary Figure S1 (see Supplementary File 1) and Figure 3A, respectively.

Within a sampling sequence, the robot was programmed to follow a series of four steps: (1) move to the safe position, (2) move to the flower’s or control’s position, start transecting and sampling humidity along the *x* axis, and then along the *z* axis, relative to the flower (Figure 3B), (3) move back to the safe position, (4) move and sample near the background probe. This was then repeated for each flower in the predefined sequence order of the day. The sequence was repeated four times without any breaks. A schematic of the motions carried out by the robot in the sampling sequence are provided in Figure 3A. The full set of robot motions over the full sampling sequence are also demonstrated in Supplementary Video 1. Next, we describe each step in a sequence in more detail.

In order to start taking measurements whilst transecting the headspace of a flower or probe, the arm moved from the safe position toward the flower or control to a point that was offset by –30 mm in the *x* axis from that flower’s transect central point (Figure 3). This position was the first sampling point on the *x* axis transect. From here the robot moved along the *x* axis horizontally through the central point until reaching a position that was offset by 30 mm relative to the transect central point (the *x* axis transect). Measurements were taken in 5 mm intervals along this transect (thirteen sampling points in total). Humidity measurements occurred regardless of whether the probe was above the flower surface at a sampling point of not (most flowers sampled are less then 60 mm wide). This meant that, even though flowers differed in size, all humidity transects were still the same size, aiding comparisons of floral humidity between different flowers and species. Following completion of measurements of the *x* axis transect, the robot moved the focal probe to a position that was offset by 5 mm in the *z* axis from that flower’s transect central point (Figure 3). This position was the first sampling point in the *z* axis transect. From here the robot moved the probe vertically upward until reaching a position offset by 30 mm in the *z* axis relative to the transect central point (the *z* axis transect). Again, measurements were taken in 5 mm intervals along this transect (six sampling points in total). This sequence of movements within the transects, as well as the direction of movements and the orientation of *x* and *z* transects relative to the flower and robot, was the same in all transects (as demonstrated in Figure 3A and Supplementary Video 1). These paired horizontal and vertical transects through the flower’s headspace are an adaptation of the similar transects carried out by von Arx et al. (2012). At each sampling point along both transects, the arm would stop the probe and take humidity measurements. This continued for 230 s, as described below. The arm moved at 3% of nominal speed (estimated to be well below 200 mm s^–1^). A third (*y* axis) transect was deemed unnecessary because all flowers and inflorescences that we sampled showed high radial symmetry.

Following completion of both transects on a flower the robot would return to the safe position 50 cm above the table, then move the focal probe to the point near to the background probe (approximately at a distance 2.5 cm from it). Here, it would carry out a probe-control measurement for 230 s, as described below. Following completion of a probe-control measurement, the robot would move the focal probe back to the ‘safe’ position starting the sampling of the next flower or control in the predefined sequence. The movement *via* the safe position when moving to and from a probe-control measurement was to avoid disturbances of the headspaces in the flower array.

Only one set of flower arrays was collected and measured per day. The initial randomized order chosen by the robot was maintained across all four replications of the measurement sequence on a sampling day. This meant that each flower was resampled four times within time intervals of 231–308 min. Following completion of the probe control measurement on the final flower in the sequence on the last replicate sample, the robot would return to the ‘safe’ position and power down. All raw data collected are available in Supplementary File 2.

### Humidity Measurement at Sampling Points and of the Background

At each sampling point along transects (the points indicated in Figure 3) the focal probe was held stationary for 230 s. The first 30 s were a non-sampled settling time, to mitigate for any disturbance in the humidity profile, before 200 s of sampling, the measurement period, where the DHT-22 probe sampled approximately 100 relative humidity measurements. These measurements were used as the ‘uncorrected relative humidity’ values of that sampling point. Whilst these measurements were taken, the background probe simultaneously recorded the background humidity for the same 200-s measurement period.

Probes can vary slightly in their estimations of the same levels of humidity (±5% according to the manufacturer’s specifications). Thus, the probe-control measurements were necessary to calculate how much the probes differed in estimates and reduce this source of inaccuracy. Upon moving the focal probe next to the background probe, the focal probe waited for 30 s before measuring humidity for 200 s, and the background probe made simultaneous measurements. Assuming that both probes are measuring a point of the same humidity during the probe-control measurements we could later compute linear regression parameters, using the MATLAB ‘*regression*’ function (MathWorks^®^, 2012), to predict one probe’s measurements from the other when in this position. This was later used to adjust the ‘uncorrected focal relative humidity’ (*f*_*uncorrected*_) values for other points using

(1) $$f_{c\,o\,r\,r\,e\,c\,t\,e\,d}=W\cdot f_{u\,n\,c\,o\,r\,r\,e\,c\,t\,e\,d}+M$$

where *W* and *M* are, respectively, the slope and intercept parameters obtained from regressing the focal probe measurements against the background probes measurements for the time period of the probe-control measurements. This focal probe correction (Eq. 1) was calculated and applied across each replicate sample of the flowers sampled on the same day (i.e., one set of *x* and *z* axis transects on every flower sampled each day). This was done to account for any possibility that the difference between probes may change over time.

### Flower Physiological Correlates

To characterize between-species differences in flower size, a flower’s horizontal span was measured on fresh unsampled flowers against a rigid tape measure, or where not possible, estimated based on miscellaneous literature records (cited in the “Results” section). Fresh flowers or literature records, as opposed to measurements from the individual flowers sampled under the robot, were used to measure flower size as such measurement might disturb floral headspace and floral humidity production. Flower or inflorescence structure, where appropriate to each species, was classified based on species descriptions from Stace (2010) for flower structure, and the inflorescence classification system of Troll (1969) and Endress (2010).

The presence of petal stomata was surveyed in a subset (*n* = 14) of the species surveyed. Petals were removed from fresh unsampled flowers, and a mold was made of the upper petal surface using dental wax (*Elite HD+ A-silicone Impression Material*, Bada Polesine, Italy). Again, fresh flowers were used to survey for stomatal presence, as opposed to those sampled under the robot, as such sampling may damage or disturb the floral headspace and floral humidity production. A cast was made from this mold using clear nail polish, and was mounted on a microscope slide using tape (*Scotch Crystal tape*, St Paul, MN, United States). Mounted casts were surveyed from petal base to tip using a light microscope at ×100 magnification, and the presence of stomata was recorded. A species’ presence of stomata was expressed as the percentage of petals surveyed that showed the presence of any stomata.

### Data Treatment and Statistical Analysis

All analyses were conducted in *R* 3.4.1 (R Development Core Team, 2017). The differences between corrected focal humidity measurements (*f*_*corrected*_) and the simultaneous background measurements (*f*_*background*_) taken throughout the transects can be expressed as a relative humidity where

(2) $$\Delta \,R\,H=f_{c\,o\,r\,r\,e\,c\,t\,e\,d}-f_{b\,a\,c\,k\,g\,r\,o\,u\,n\,d}$$

Where Δ*RH* > 0 an increase in relative humidity is detected by the focal probe.

#### Assessment of Floral Humidity

The mean difference between the focal and background probe (Δ*RH*, Eq. 2) was calculated within each measurement period (the *c.*100 measurements made at each sampling point, on each replicate sample, on each individual flower). These averages at each measurement period, were then used for analyses of humidity structure. For each flower species (and control), a series of linear models describing different humidity structures were fitted to this *x* and *z* axis data, as described below. How well these different models described humidity structure was compared using AIC to identify the best-fitting model of *x* and *z* axis humidity structure for each species (and control). These best-fitting models were then used to estimate humidity intensity for each species (see below). The most complex model (the ‘full model’) fitted to *x* axis transect data of each species had a quadratic structure, the most complex model fitted to the *z* axis data had a logarithmic structure. These full models also allowed humidity to vary between repeated transects. All other models represented simplified versions of the full models. These quadratic and logarithmic models were chosen over more complicated curve fitting methods and functions as, in addition to their relative simplicity, these functions easily condense to other structures as models are simplified (see “Statistical Models” section below). This allows the identity of best-fitting models to indicate differences in floral humidity structure, and changes in humidity over repeat transects. Throughout all floral humidity models, flower identity was included as a random factor influencing the intercept, or amount of humidity produced.

#### Evaluation of Robot Measurements

To assess the repeatability of the measurements of the difference between focal and simultaneous background humidity, Δ*RH*, the residual variation taken at the same sampling point along our transects was calculated (Nakagawa and Schielzeth, 2010), using the *rpt* function (with 100 bootstrap model repeats) within *rptR* 0.9.21 (Stoffel et al., 2017) using each set of *c*.100 measurements taken during the measurement period at each sampling point as the replication level of interest. The repeatability of measurements within each measurement period from all transects, across all species and controls were assessed together.

The turbulence generated by the movement of the probe may cause humidity to not settle by the end of the 30-s settling time, which could cause a change in humidity measurements at the focal probe between the start of a measurement period (where remaining turbulence would be greatest), and the end (where it would be most stable). It is possible this difference may differ dependent upon the humidity at a sampling point: if little water is present in the air, disruption of that area may affect humidity estimates less than more humid air. To assess the effect of remaining turbulence, the change across a measurement period should therefore be assessed while accounting for how humid the disturbed area is. Furthermore, the directionality (positive or negative) of this change in humidity may differ depending on how the air mixes as a result of turbulence and the humidity of air being mixed in. If turbulence brings in more humid air, humidity may be raised, while the reverse might be expected if less humid air is brought in. To avoid turbulence effects being obscured by conflicting (positive or negative) changes in humidity, the non-directional change in humidity across a measurement period should be considered, rather than just the change in humidity. The presence and extent of remaining turbulence after the settling time was assessed by calculating the means of the focal humidity (*f*_*uncorrected*_) measurements taken during the first 20 s and the last 20 s at each measurement period, denoted $f_{f\,o\,c\,a\,l}^{f\,i\,r\,s\,t}$ and $f_{f\,o\,c\,a\,l}^{l\,a\,s\,t}$, respectively. The non-directional change in focal humidity between the beginning and end of each measurement period can then be calculated as

(3) $$f_{f\,o\,c\,a\,l}^{c\,h\,a\,n\,g\,e}=\sqrt{{\left(f_{f\,o\,c\,a\,l}^{f\,i\,r\,s\,t}-f_{f\,o\,c\,a\,l}^{l\,a\,s\,t}\right)}^{2}}$$

$f_{f\,o\,c\,a\,l}^{c\,h\,a\,n\,g\,e}$ is thus an indicator how much humidity differs between the start and end of a measurement period. The mean focal humidity across the whole of each measurement period (200 s, *c.* 100 measurements), $f_{f\,o\,c\,a\,l}^{m\,e\,a\,n}$, was also calculated. A linear regression model describing how the $f_{f\,o\,c\,a\,l}^{c\,h\,a\,n\,g\,e}$ value of a measurement period changes with $f_{f\,o\,c\,a\,l}^{m\,e\,a\,n}$ was fitted to this data. The extent of $f_{f\,o\,c\,a\,l}^{c\,h\,a\,n\,g\,e}$, and how this is influenced by $f_{f\,o\,c\,a\,l}^{m\,e\,a\,n}$ was then evaluated by testing the significance of this model’s parameters.

#### Statistical Models

The full model fitted to the *x* axis transect of a given species or control is

(4) $$\Delta \,R\,H_{x\,n\,t}=I_{x}+i_{x\,t}+\left(A_{x}+a_{x}\right)\,X+\left(B_{x}+b_{x}\right)\,X^{2}+\nu_{x\,n}$$

where *X* refers to the *x* axis offset of the sampling point (from –30 mm to +30 mm in steps of 5 mm) relative to the transect central point (where *X* = 0), Figure 3B. The value of *t* varies from 1 to 4, as the sampling sequence was repeated four times for each flower or control: 1 being the first run of a sampling sequence; 2, 3, and 4 being the second, third, and fourth repetitions, respectively. Δ*RH*_*xnt*_ is the mean Δ*RH* for the measurement period at sampling point *X*, on transect repeat sample *t*, on flower *n*. *A*_*x*_ and *B*_*x*_ are parameters that describe the positioning and slope of the *x* axis humidity profile in the initial transect. Parameter *I*_*x*_ is the model intercept in the initial transect. *I*_*x*_ is modified by random factor *v*_*xn*_, which represents the change in model intercept, and consequently the intensity of humidity generated, by individual flower (or control tube) *n* on the *x* axis transect. Further random factors accounting for individual variation in the shape of humidity structure were not included, in order to simplify models. As flowers may show changes in humidity produced over time across replicate transects, *A*_*x*_, *B*_*x*_, and *I*_*x*_ are also modified by parameters *a*_*x*_, *b*_*x*_, and *i*_*x*_, which represent the change in the offset, slope and intercept, respectively (relative to *A*_*x*_, *B*_*x*_ and *I*_*x*_, respectively) between first and latter replicate transects. The values of *a*_*x*_, *b*_*x*_ and *i*_*x*_ within each replicate transect are modified as follows:

(5) $$a_{x}=F\,\left(g_{2\,x}\right)+S\,\left(g_{3\,x}\right)+E\,\left(g_{4\,x}\right),$$

(6) $$b_{x}=F\,\left(c_{2\,x}\right)+S\,\left(c_{3\,x}\right)+E\,\left(c_{4\,x}\right),$$

(7) $$i_{x}=F\,\left(r_{2\,x}\right)+S\,\left(r_{3\,x}\right)+E\,\left(r_{4\,x}\right)$$

where *g*_*tx*_, *c*_*tx*_, and *r*_*tx*_ are the values of *a*_*x*_, *b*_*x*_, and *i*_*x*_ during repeat sample transect *t*, when

(8) $$F=\left\{ \begin{array}{cc} 0,t\neq 2\; & \\ 1,t=2\; & \end{array}\right.,$$

(9) $$S=\left\{ \begin{array}{cc} 0,t\neq 3\; & \\ 1,t=3\; & \end{array}\right.,$$

(10) $$E=\left\{ \begin{array}{cc} 0,t\neq 4\; & \\ 1,t=5\; & \end{array}\right..$$

Consequently *g*_*tx*_, *c*_*tx*_, and *r*_*tx*_ are the changes applied to *A*_*x*_, *B*_*x*_, and *I*_*x*_ in transect *t*. Here parameters *A*_*x*_, *B*_*x*_, *I*_*x*_, *v*_*xn*_ and all iterations of *g*_*tx*_, *c*_*tx*_, and *r*_*tx*_ are parameters to be estimated. The *x* axis model described by Eq. (4) assumes a degree of symmetry in humidity structure, this is due to the high level of radial symmetry shown by all species sampled. Although some degree of asymmetry, off-center positioning, is included in the model (primarily through the effects of parameters *A*_*x*_ and *a*_*x*_).

The full model applied to the *z* axis transect is

(11) $$\Delta \,R\,H_{z\,n\,t}=I_{z}+i_{z}+\left(B_{z}+b_{z}\right)\,\ln Z+\nu_{z\,n}$$

Here, parameter *Z* refers to the sampling point’s *z* axis offset relative to the transect central point (Figure 3B). All other parameters (*I*_*z*_, *i*_*z*_, *B*_*z*_, *b*_*z*_, and *v*_*zn*_) function in the same manner described for respective parameters in the *x* axis model. Here, parameters *I*_*z*_, *B*_*z*_, *v*_*zn*_ and all iterations of *c*_*tz*_ and *r*_*tz*_ are parameters to be estimated.

#### Model Selection Process

The full models for the *x* and *z* transects described in Eqs (4) and (11), as well as simpler versions of these models, were fitted to the *x* and *z* transect data of each species and each control group. Simpler models were achieved by removing certain parameters from the full models, done by forcing these parameters to have values of zero. Eleven *x* axis models and five *z* axis models were compared for each species. These models are summarized in Tables 2, 3 for the *x* and *z* axis models, respectively. These models were then compared using AIC to select the best models for the *x* and *z* transects of each flower species following the guidelines within Richards (2008).

**TABLE 2:** The models fitted to each species’ and control group’s *x* axis transect humidity data.

**Model**	**Omitted parameters**	**Model description**
m0	*A*_*x*_, *B*_*x*_, *r*_*tx*_, *c*_*tx*_, *g*_*tx*_	Flat linear model with no influence of replicate transects
m1	*B*_*x*_, *r*_*tx*_, *c*_*tx*_, *g*_*tx*_	Linear model with no influence of replicate transects
m2	*A*_*x*_, *r*_*tx*_, *c*_*tx*_, *g*_*tx*_	Quadratic model with no influence of replicate transects
m3	*r*_*tx*_, *c*_*tx*_, *g*_*tx*_	Quadratic model with an *x* axis offset with no influence of replicate transects
m4	*A*_*x*_, *B*_*x*_, *c*_*tx*_, *g*_*tx*_	Flat linear model with differing intercepts between replicate transects
m5	*B*_*x*_, *c*_*tx*_, *g*_*tx*_	Linear model with differing intercepts between replicate transects
m6	*A*_*x*_, *c*_*tx*_, *g*_*tx*_	Quadratic model with differing intercepts between replicate transects
m7	*c*_*tx*_, *g*_*tx*_	Quadratic model with an *x* axis offset and differing intercepts between replicate transects
m8	*B*_*x*_, *c*_*tx*_	Linear model with interacting effects of replicate transects
m9	*A*_*x*_, *g*_*tx*_	Quadratic model with interacting effects of replicate transects
m10	None	The full model: Quadratic model with an *x* axis offset and interacting effects of replicate transects

*Model name, the omitted parameters from the full model and a model description is given.*

**TABLE 3:** The models fitted to each species’ and control group *z* axis transect humidity data.

**Model**	**Omitted parameters**	**Model description**
z0	*B*_*z*_, *r*_*tz*_, *c*_*tz*_	Flat linear model with no influence of replicate transects
z1	*r*_*tz*_, *c*_*tz*_	Logarithmic model with no influence of replicate transects
z2	*B*_*z*_, *c*_*tz*_	Flat linear model with differing intercepts between replicate transects
z3	*c*_*tz*_	Logarithmic model with differing intercepts between replicate transects
z4	None	Logarithmic model with interacting effects of replicate transects

*Model name, the omitted parameters from the full model and a model description is given.*

#### Summary Value Calculation

To evaluate the intensity of humidity generated by each flower species and by controls $\Delta \,R\,H_{x}^{\max}$, the peak mean humidity difference across the *x* transect as predicted by the best-fitting humidity model, was calculated for each flower species. This $\Delta \,R\,H_{x}^{\max}$ value indicates on average the largest change in humidity generated by each species during our sampling. Calculation of $\Delta \,R\,H_{x}^{\max}$ began by calculating $X_{t}^{\max}$, the *x* axis offset (parameter *X*) value that the best-fitting model predicted to give the largest difference in humidity on transect *t*, for each species. $\Delta \,R\,H_{x}^{\max}$ and $X_{t}^{\max}$ were calculated using the parameter values estimated by the best-fitting *x* axis model of each species. If the best-fitting *x* axis model was m0 or m4 (see Table 2), a value of 0 was used as $X_{t}^{\max}$, as any value of *X* could potentially be used for $X_{t}^{\max}$. In species where the best-fitting model showed a linear relationship, $X_{t}^{\max}$ would be either 30 or −30 (depending on whether *A*_*x*_ + *a*_*x*_ came to a positive or negative value, respectively). In species where the *x* axis showed a quadratic relationship in the best-fitting model, $X_{t}^{\max}$ would be the *X* value of the vertex of the quadratic curve described by Eq. (4) (as all species favoring quadratic models showed negative values for *B*_*x*_ + *b*_*x*_). Due to this a formula for $X_{t}^{\max}$ of a species showing a quadratic best-fitting *x* axis model can be derived, as detailed in Appendix S3 (in Supplementary File 1):

(12) $$X_{t}^{\max}=-\frac{\left(A_{x}+a_{x}\right)}{2\,\left(B_{x}+b_{x}\right)}$$

$X_{t}^{\max}$ can then be calculated using the parameter values estimated by the best-fitting *x* axis model of each species.

Once $X_{t}^{\max}$ is calculated, $\Delta \,R\,H_{x}^{\max}$ of a species could then be estimated by inserting $X_{t}^{\max}$ into Eq. (4) and using the parameter estimates of the best-fitting model, with ν_*xn*_ was set to zero:

(13) $$\Delta \,R\,H_{x}^{m\,a\,x}=I_{x}+i_{x\,t}+\left(A_{x}+a_{x}\right)\,X_{t}^{m\,a\,x}+\left(B_{x}+b_{x}\right)\,X_{t}^{m\,a\,x^{2}}$$

In instances where replicate transects had an influence on Δ*RH*_*x*_, the $X_{t}^{m\,a\,x}$ and $\Delta \,R\,H_{x}^{m\,a\,x}$ would be calculated for each replicate transect and the largest $\Delta \,R\,H_{x}^{m\,a\,x}$ value used. In instances where multiple comparable models were best-fitting these summary values would be calculated with whichever of these comparable best models had the lowest AIC.

#### Comparative Influences on Floral Humidity

A phylogenetically controlled analysis was carried out to assess the extent to which floral humidity produced by species ($\Delta \,R\,H_{x}^{m\,a\,x}$) corresponds with the species’ flower span (measured in mm), floral type (taken as either a single flower or an inflorescence) and the growth conditions of flowers before sampling (whether it was grown outside or not). This analysis may identify trends that explain differences in floral humidity production between species, while taking into account the shared evolutionary history, and likely physiological similarities, of species.

Phylogenies were constructed within *R* 3.4.1 (R Development Core Team, 2017) using the megaphylogeny and *S.Phylomaker* algorithm described by Qian and Jin (2016). A number of the species studied were either absent from the megaphylogeny or only identified to the genus level, but at least congeneric sister species was present allowing a degree of accuracy in placement. The data for the horticultural cultivar *Geranium* ‘Roxanne’ was not considered in these analyses as it was not possible to place this cultivar within the genus. The default algorithm given by Qian and Jin (2016) constructs phylogenies based on the position sister species using three separate rule sets. We used a tree generated by their second scenario, where an absent species is placed randomly within its genus, but trees from the other scenarios suggested were also considered, and gave identical results.

How $\Delta \,R\,H_{x}^{m\,a\,x}$ (see Eq. 13) was affected by mean flower span, floral type, and whether plants had grown in field conditions or in a greenhouse was tested using a phylogenetically controlled generalized least squares regression (Grafen, 1989; Martins and Hansen, 1997; Symonds and Blomberg, 2014) fit using a maximum likelihood model and run within *nlme* 3.1-137 (Pinheiro et al., 2018), assuming a correlation matrix based on either Brownian Motion (BM) or an Ornstein Uhlenbeck (OU) process with an estimate of α generated with *ape* 5.1 (Paradis et al., 2004), with floral type and growth environment coded as binary dummy variables. $\Delta \,R\,H_{x}^{m\,a\,x}$ was log-transformed so that test assumptions were met (Mundry, 2014). Models with OU correlation were compared to those with BM correlation using likelihood tests and AICs. The effect sizes of the full model that best explained the data were assessed to evaluate the influence of variables (span, type, growth conditions) on species $\Delta \,R\,H_{x}^{m\,a\,x}$. This model was then compared to a corresponding null model, where $\Delta \,R\,H_{x}^{m\,a\,x}$ was fit to the grand mean, to verify the influence of these factors.

## Results

### Evaluation of Robot Measurements

The robot arm showed high repeatability over each set of approximately 100 measurements made in the same measurement period (*R* = 0.971, *SE* < 0.001, 95% CI = [0.971, 0.972], *p* < 0.001). This means that within each measurement period the majority of measurements are about the mean value. This confirms the robot’s consistency of measurements and validates our decision to use mean values of each measurement period for our analyses of humidity structure, as measurements within each period are largely similar.

Analysis of $f_{f\,o\,c\,a\,l}^{c\,h\,a\,n\,g\,e}$ values found focal humidity measurements from the start of measurement periods differed by a small amount from those taken at the end (Table 4). This suggests there may be small amounts of turbulence from robot movement remaining after the 30-s waiting time. However, this change across measurement periods was small (effect sizes in Table 4). Furthermore, the high repeatability across measurement periods suggests humidity was settled across the majority of measurement periods. Humidity measurement accuracy might be improved by a longer waiting time or further reduced robot speed, however, such changes in the protocol are likely have very slight effects on humidity measurements.

**TABLE 4:** Summary of the parameter effects of the linear regression model fitted to the mean focal humidity measurements taken during the first and last 20 s of each measurement period.

**Parameter**	**Estimate**	**Standard error**	**Confidence intervals**		***t*_21582_**	***p***
			**2.5%**	**97.5%**		
Model Intercept	0.091	0.0056	0.0796	0.1016	16.12	<0.01
$f_{f\,o\,c\,a\,l}^{m\,e\,a\,n}$ (%)	0.001	0.0001	0.0006	0.0011	7.61	<0.01

### Determining the Level of Extraneous Humidity

To detect potential sources of extraneous humidity, we conducted measurements of control samples, such as empty or water filled tubes without flowers placed inside the array (Figure 2). The best-fitting models, $X_{t}^{m\,a\,x}$, and $\Delta \,R\,H_{x}^{m\,a\,x}$ values for the controls are shown within Table 5, the parameter values for best-fitting *x* and *z* axis models and humidity structure for controls are given in Appendix S4 and AIC tables for model selection are found in Appendix S2 in Supplementary File 1. Plots of humidity structure (in the *x* and *z* axis transects) for each control are given in Supplementary File 3. The transects of the control groups confirm that the arm detects a humidity source if present, for example water filled tubes (TW and TWL controls in Table 5), but detects little difference when sources of humidity are absent, as in empty tubes (T and TL controls).

**TABLE 5:** The results of the humidity survey, summarized by $\Delta \,R\,H_{x}^{m\,a\,x}$, alongside physiological correlates.

**Rank**	**Species**	**Structure**	**Mean span mm (*n*/source)**	**Stomata (*n*)**	**Best-fitting model for**		**$X_{t}^{m\,a\,x}$**	**$\Delta \,R\,H_{x}^{m\,a\,x}$**
					***x* axis**	***z* axis**		
1	*Fuchsia* sp.	Funnel	33.7 (7)		m0_*F*_	z2_*F*_	0_(Z)_	0.05
	T control				m7_*Q*_	z1_*L*_	−5.01_3_	0.06
	TWLP control				m5_*L*_, m6_*Q*_	z2_*F*_	−30_3_	0.09
2	*Nicotiana tabacum*	Funnel	23.6 (5)		m2_*Q*_	z2_*F*_	0_(Z)_	0.10
	TL control				m5_*L*_	z2_*F*_	−30_3_	0.10
	TLP control				m4_*F*_	z2_*F*_	0_1_	0.14
3	*Vinca herbacea*	Bell	48.6 (12)	0% (5)	m7_*Q*_	z0_*F*_	−2.49_2_	0.24
4	*Allium ursinum*	Unfused	20 (a)		m2_*Q*_	z2_*F*_	0_(Z)_	0.24
5	*Nepenthes* sp.	Unfused	80 (b)		m2_*Q*_	z2_*F*_	0_(Z)_	0.26
6	*Papaver rhoeas*	Unfused	56.4 (9)	100% (5)	m3_*Q*_	z2_*F*_	−4.35_(Z)_	0.29
7	*Euphorbia milii*	Compound inflorescence	12 (c)		m6_*Q*_	z2_*F*_	0_3_	0.29
8	*Cyanus montanus*	Compound inflorescence	53.4 (11)		m7_*Q*_	z2_*F*_	−1.88_2_	0.31
9	*Abutilon* × *milleri* hort.	Funnel	30.8 (6)	0% (4)	m6_*Q*_	z2_*F*_	0_1_	0.32
10	*Campanula* sp.	Bell	20.7 (3)		m2_*Q*_	z2_*F*_	0_(Z)_	0.36
11	*Linum grandiflorum*	Unfused	38 (3)	0% (2)	m10_*Q*_	z3_*L*_	7.22_2_	0.36
12	*Geranium robertianum*	Unfused	15.4 (11)		m2_*Q*_	z2_*F*_	0_(Z)_	0.41
	TWL control				m2_*Q*_	z0_*F*_	0	0.46
13	*Tulbaghia violacea*	Funnel	29 (9)		m9_*Q*_	z2_*F*_	0_1_	0.52
14	*Papaver cambricum*	Unfused	48.3 (8)	100% (5)	m2_*Q*_	z1_*L*_	0	0.58
15	*Bellis perennis*	Compound inflorescence	18.1 (14)	0% (3)	m2_*Q*_	z3_*L*_	0_(Z)_	0.58
16	*Epilobium hirsutum*	Funnel	15.1 (7)		m2_*Q*_	z2_*F*_	0_(Z)_	0.59
17	*Trifolium pratense*	Umbel inflorescence	20.1 (9)		m9_*Q*_	z2_*F*_	0_4_	0.61
18	*Lilium* sp.	Funnel	93 (5)		m8_*L*_	z2_*F*_	−30_4_	0.65
19	*Clematis chinensis*	Unfused	50 (d)		m7_*Q*_	z2_*F*_	−2.91_1_	0.66
20	*Cistus* ‘greyswood pink’	Unfused	43.6 (11)		m2_*Q*_	z3_*L*_	0_(Z)_	0.66
21	*Cosmos bipinnatus*	Compound inflorescence	92.5 (6)		m7_*Q*_	z1_*L*_	2.99_2_	0.67
22	*Geranium* ‘Roxanne’	Unfused	42.6 (8)	0% (4)	m7_*Q*_	z3_*L*_	−1.66_3_	0.67
23	*Potentilla* sp.	Unfused	29 (12)		m7_*Q*_	z1_*L*_	1.28_1_	0.70
24	*Coreopsis* sp.	Compound inflorescence	46.8 (4)	0% (2)	m3_*Q*_	z3_*L*_	0.94_(Z)_	0.71
25	*Lavandula angustifolia*	Racemose inflorescence	18.6 (10)		m6_*Q*_	z2_*F*_	0_4_	0.72
26	*Geranium sanguineum*	Unfused	41 (12)		m9_*Q*_	z3_*L*_	0_2_	0.79
27	*Linum usitatissimum*	Unfused	16.7 (6)		m9_*Q*_	z2_*F*_	0_2_	0.80
28	*Convolvulus sabatius*	Funnel	32.8 (12)	0% (4)	m9_*Q*_	z3_*L*_	0_1_	0.87
29	*Cyanus segetum*	Compound inflorescence	36.4 (14)		m6_*Q*_	z0_*F*_	0_1_	1.10
	TW Control				m2_*Q*_	z1_*L*_	0	1.17
30	*Osteospermum* sp.	Compound inflorescence	51.9 (10)	20% (5)	m10_*Q*_	z4_*L*_	5.49_3_	1.20
31	*Rudbeckia hirta*	Compound inflorescence	56.3 (10)		m7_*Q*_	z1_*L*_	2.97_2_	1.25
32	*Scabiosa* sp.	Compound inflorescence	39.6 (5)		m3_*Q*_	z1_*L*_	1.61	1.36
33	*Lantana* sp.	Umbel inflorescence	42.3 (11)		m2_*Q*_	z1_*L*_	0	1.47
34	*Achillea millefolium*	Umbel inflorescence	33.3 (9)		m3_*Q*_	z1_*L*_	1.69	1.73
35	*Leucanthemum vulgare*	Compound inflorescence	47.2 (6)		m7_*Q*_	z4_*L*_	2.17_1_	1.79
36	*Oenothera caespitosa*	Funnel	54.2 (5)		m10_*Q*_	z1_*L*_	2.54_1_	1.79
37	*Ranunculus lingua*	Unfused	35.7 (7)		m7_*Q*_	z3_*L*_	2.61_2_	3.16
38	*Eschscholzia californica*	Unfused	48.8 (9)	80% (5)	m7_*Q*_	z4_*L*_	21.49_4_	3.24
39	*Taraxacum* agg.	Compound inflorescence	39.3 (9)	0% (4)	m9_*Q*_	z4_*L*_	0_4_	3.35
40	*Ranunculus acris*	Unfused	24 (19)	0% (3)	m9_*Q*_	z3_*L*_	0_2_	3.41
41	*Xerochrysum bracteatum*	Compound inflorescence	48.4 (9)	0% (3)	m3_*Q*_	z1_*L*_	1.41	3.67
42	*Calystegia silvatica*	Funnel	61.8 (12)		m8_*L*_	z1_*L*_	30_3_	3.71

*Species are ordered by ascending rank order of $\Delta \,R\,H_{x}^{m\,a\,x}$, with the six controls included for comparison (in the bracket after ‘Control,’ T, horticultural tube; W, water; L, tube lid present; P, putty present). Bracketed values after the mean span and stomata occurrence values are the number of flowers and petals sampled for span or stomatal presence. Where span was taken from a different source this is indicated by a letter where: a, Clapham et al. (1981); b, Cartalano and Kruetr (2010); c, Huxley et al. (1999); and d, Plants Database (2018). Subscript letters following best-fitting models indicate shape of that model: F, flat models; L, linear models; and Q, quadratic models (note quadratic models were not fitted to the z axis). Subscript values next to $X_{t}^{m\,a\,x}$ values indicate replicate effects: the number itself referring to the value of t, which replicate transect, at which $\Delta \,R\,H_{x}^{m\,a\,x}$ was found; (Z) indicates replicate effects only in the z axis model; no subscript values indicates replicate transects have no effect on humidity.*

When the horticulture tubes had no water in them we saw little change in humidity between focal and background probes, hence the low $\Delta \,R\,H_{x}^{m\,a\,x}$ values of the T and TL controls (Table 5). This suggests that very little of the observed humidity differences are the result of humidity varying within the room due to air mixing. The slight differences between the T and TL controls are likely to be due to influences on changes in airflow about the tubes due to the addition of the tube’s lid. The TLP control shows a slight increase compared to the TL control, suggesting the *blu tack* putty gives off a small amount of water as it dries out.

The addition of water to the controls increased humidity, as seen by the higher $\Delta \,R\,H_{x}^{m\,a\,x}$ values of the TW and TWL controls compared to the empty T and TL controls (Table 5). This shows that there could be potentially a small amount of humidity generated by the water filled horticulture tubes flowers were placed within. However, the addition of the lid to a water filled tube decreased the humidity generated, by limiting the escape of water vapor to the hole in the tube’s lid. This resulted in the lower $\Delta \,R\,H_{x}^{m\,a\,x}$ of the TWL control compared to the TW control (Table 5). Humidity intensity decreased between the TWL and TWLP controls (Table 5). This is likely to be because, despite the putty giving off water vapor, it also further obscures the hole in the lid of the horticultural tube, limiting escape to water vapor from the tube in the same way as the lid.

Taken together, it seems that at most humidity extraneous to the flowers from the horticulture tube will be similar to that of the TWL control ($\Delta \,R\,H_{x}^{m\,a\,x}$ = 0.46%). However, as flowers also obscure the hole in the horticultural tube lid, this extraneous humidity may be lessened in the same manner as in TWLP control. We therefore conclude that the level of extraneous humidity in our study is negligibly low.

### Floral Humidity

**FIGURE 4:**
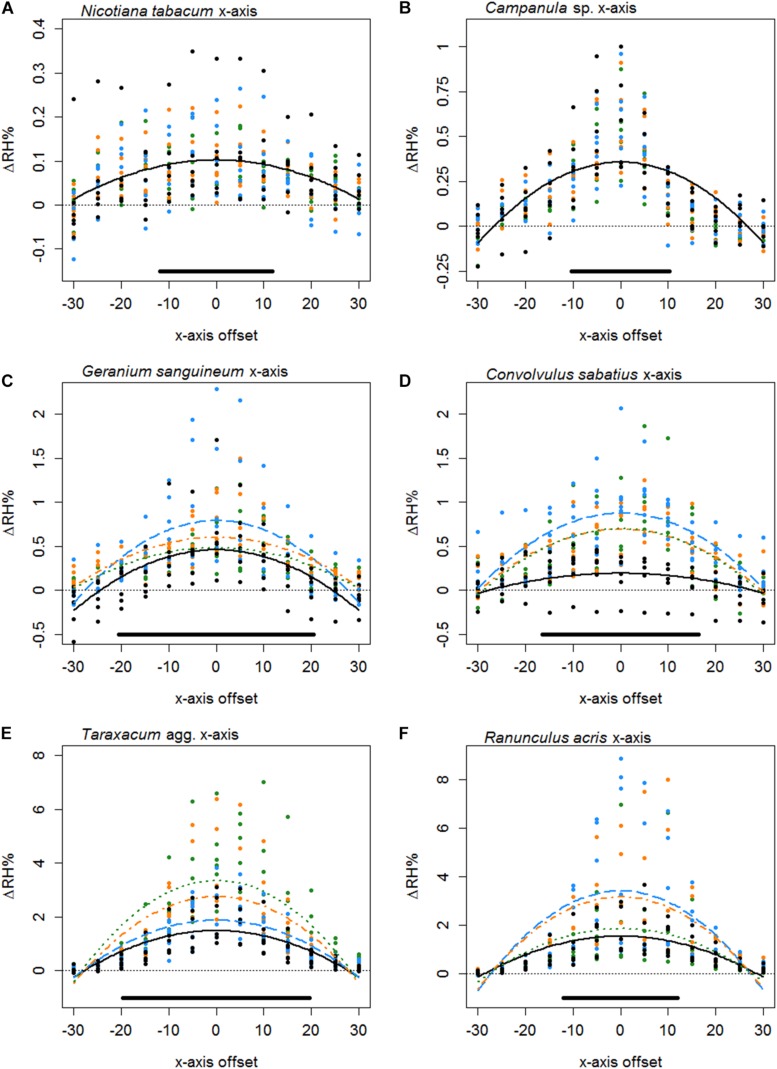
The difference in humidity relative to the backgrounds (Δ*RH*) or the *x* axis transects of *Nicotiana tabacum* (**A**), *Campanula* sp. (**B**), *Geranium sanguineum* (**C**), *Convolvulus sabatius* (**D**), *Taraxacum* agg. (**E**), and *Ranunculus acris* (**F**). Plots for other species surveyed and controls are given in Supplementary File 3. All axis offsets are relative to the transect central point and in millimeters. The thin dotted line indicates a 0% change in humidity (the background level). Bold lines indicate the mean change in humidity as predicted by the best-fitting model for that flower. Color and dashing of bold lines and points indicate the replicate transect: solid black, first transect; long-dash blue, second transect; dash-dot orange, third transect; dotted green, fourth transect. The solid bar above the *x* axis indicates the mean flower span for that species relative to the *x* axis.

The transect measurements across flower headspaces revealed major differences in the humidity levels between flower species (Table 5). Values for estimated parameters of our best-fitting models can be found in Appendix S4 and AIC tables for each species are given in Appendix S1 (both in Supplementary File 1). In some species the floral humidity detected was lower than or comparable to extraneous humidity expected from the horticultural tube (where $\Delta \,R\,H_{x}^{m\,a\,x}$ = 0.46%: indicated as ‘TWL Control’ in Table 5) and it was difficult to confirm the floral unit is the source of the humidity. However, 30 species produced floral humidity at an intensity that exceeded this expected humidity. Thirteen species produced floral humidity of a greater intensity than that detected from the TW control ($\Delta \,R\,H_{x}^{m\,a\,x}$ > 1.17%), the water filled horticulture tube unobscured by the tube’s lid or the flower, the largest $\Delta \,R\,H_{x}^{m\,a\,x}$ produced by any control. In these cases it seems very likely the flower is the humidity source. Six of these species produced notably higher floral humidity intensities ($\Delta \,R\,H_{x}^{m\,a\,x}$ > 3%) more similar to the levels moth pollinators have been reported to respond to (*Ranunculus lingua*, *Eschscholzia californica*, *Taraxacum* agg., *Ranunculus acris*, *Xerochrysum bracteatum*, and *Calystegia silvatica*). Frequently, there was increased variation in humidity about the center of individual flowers, with a positive skew, relative to the rest of the transect (Figure 4). Due to this skew, $\Delta \,R\,H_{x}^{m\,a\,x}$ may represent a more conservative estimate of the humidity produced by some species. Regardless, even when based on this, potentially conservative, estimate species clearly varied in humidity levels produced.

Typically, the humidity structure detected in the *x* axis transect showed a quadratic relationship between the *x* axis offset and Δ*RH*_*x*_. Of the 42 species studied, only three (*Fuchsia* sp., *Lilium* sp., and *Calystegia silvatica*) had non-quadratic best-fitting *x* axis models, as shown in Table 5, all other best-fitting models included parameter *B*_*x*_ in some way. The *x* axis humidity structures of six species are given in Figure 4. The point where humidity intensity was greatest, $X_{t}^{m\,a\,x}$, was the transect central point (*X* = 0) for 22 species, Table 5. In species where $X_{t}^{m\,a\,x}$ did not equal 0, $X_{t}^{m\,a\,x}$ was normally only slightly offset, with all but five species showing $X_{t}^{m\,a\,x}$ to be less than 5 mm offset from the transect central point (Table 5).

**FIGURE 5:**
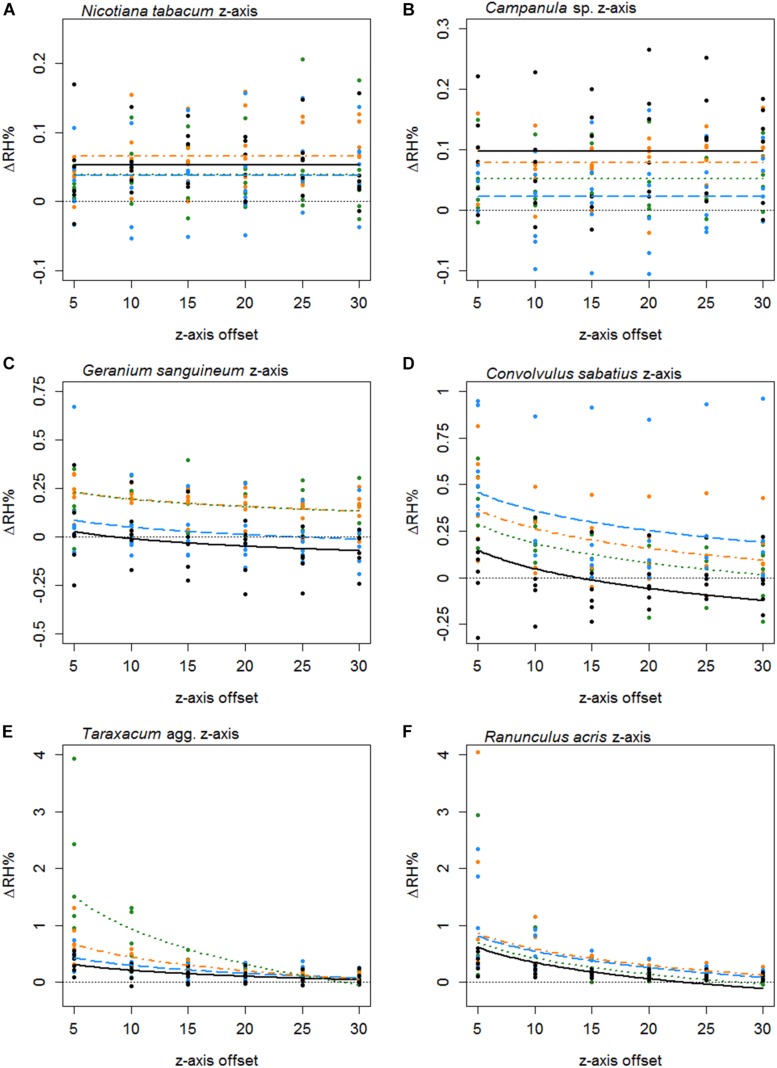
The difference in humidity relative to the backgrounds (Δ*RH*) or the *z* axis transects of *Nicotiana tabacum* (**A**), *Campanula* sp. (**B**), *Geranium sanguineum* (**C**), *Convolvulus sabatius* (**D**), *Taraxacum* agg. (**E**), and *Ranunculus acris* (**F**). Plots for other species surveyed and controls are given in Supplementary File 3. All axis offsets are relative to the transect central point and in millimeters. The thin dotted line indicates a 0% change in humidity (the background level). Bold lines indicate the mean change in humidity as predicted by the best-fitting model for that flower. Color and dashing of bold lines and points indicate the replicate transect: solid black, first transect; long-dash blue, second transect; dash-dot orange, third transect; dotted green, fourth transect.

Humidity profiles in the *z* axis were less consistent in structure. The *z* axis structures of six species are given in Figure 5. In 25 species, the best-fitting *z* axis models showed a logarithmic relationship with humidity declining with increased distance from the flower (increased *z* axis offset). In most instances humidity reaching background levels by the end of the 30 mm transect. Although 17 species favored a flat *z* axis model where humidity remained level across the transect (Table 5) as seen in Figures 5A,B.

Thirty-seven species favored models with changes in humidity with replicate transects in at least one axis, 19 of which favored changes with replicate transects in both axis. Most changes with replicate transects were changes in floral humidity intensity (non-interacting effects determined by *r*), but in 13 species (ten in *x* axis only, two in *z* axis only, one in both) showed some changes in structure of floral humidity with replicate transects (interacting effects determined by *g* and *c*). In most species the humidity decreased in the later replicate transects (Figures 4, 5), but in several species, there was an increase in the humidity signal after the first transect, as seen in *Ranunculus acris* (Figures 4F, 5F). In most species (all except seven) the largest humidity signal was detected in the first or second replicate transect (Table 5).

### Comparative Influences on Floral Humidity

The fitted full phylogenetically controlled model with OU correlation (estimated *α* = 31) explained the data better than the model with BM correlation (AIC = 119.0 vs. 173.7, respectively, ΔAIC = 54.7, $\chi_{1}^{2}$ = 56.64, *p* < 0.001). The model parameter effects are summarized in Table 6. Explanatory variables had no significant effects on $\Delta \,R\,H_{x}^{m\,a\,x}$ within the OU full model, leaving the model intercept as the only significant effect. Comparing the OU full model to a null model with an OU correlation confirmed that there was no measurable effect of the explanatory variables on humidity (AIC = 119.05 full vs. 119.50 null, ΔAIC = 0.45, $\chi_{3}^{2}$ = 6.45, *p* = 0.092).

**TABLE 6:** Phylogenetically controlled effect sizes of floral characteristics on $\Delta \,R\,H_{x}^{m\,a\,x}$.

**Parameter**	**Model value**	**Standard error**	**Back-transformed effect size (RH%)**	**Back-transformed confidence intervals**		***t*_37_**	***p***
				**2.5%**	**97.5%**		
Model intercept	–1.23	0.48	0.29	0.11	0.74	–2.60	0.01
Grown outside	0.28	0.34	0.09	0.67	2.58	0.81	0.42
Floral type	0.55	0.31	0.21	0.95	3.17	1.79	0.08
Span (mm)	0.01	0.01	*e*^0.01(*span*)^	98	1.03	1.61	0.12

*The table reports the parameter effects of the phylogenetically controlled generalized least squares regression model.*

## Discussion

This survey demonstrates that many species across a wide range of the angiosperms produce floral humidity (Table 5), supporting suggestions by earlier researchers that floral humidity is widespread (Corbet et al., 1979a, b; von Arx, 2013). We found variation in intensity and spatial distribution of humidity in the flower’s headspace. This variation in floral humidity suggests that insects could benefit from detecting such differences and using them as short-distance cues. This humidity variation between species may also reflect differing requirements or strategies to maintain floral temperature and pollen viability and may even contribute to differing species vulnerability infection by fungal pathogens. The effects floral humidity has on these various factors remains largely unexplored. However, these influences of floral humidity may be widespread due to the apparent frequency of its occurrence.

To conduct our fine-scaled measurements in a controlled environment for comparisons across species, we cut flowers from the plant. This can potentially have some impact on their hormonal physiology or cause interference with water uptake (van Doorn, 1997). However, problems with water uptake take time to develop and experiments with cut flowers show they function normally in terms of transpiration, showing normal daily cycles (Lü et al., 2011; Fanourakis et al., 2012; Huang et al., 2018). Within the timescales of our sampling (less than a day), flowers were likely to be functioning normally in terms of transpiration and water uptake for at least the earlier transects. Species whose flowers were picked from the outside showed no difference in capacity to produce humidity than those that grew indoors (Table 6). This suggests the floral humidity detected is unlikely to be an artifact of flower treatment or nectar being allowed to accumulate to unnatural levels due to lack of exposure to floral visitors.

Nectar volume will have influenced humidity, but we did not attempt to remove it, considering it to be a biologically relevant contributor to the humidity produced by a flower and part of the full floral stimulus that would be presented to a pollinator (also its removal could damage the structure and resulting humidity profile of an inflorescence). We note here that we deliberately excluded leaves from our survey, although these were considered with *O. caespitosa* in the study conducted by von Arx et al. (2012). They found that petals did not differ significantly in their vapor emission from leaves. Furthermore, it is difficult to quantify the effects of plant structure, positioning of flowers, and distance from leaves. Thus although in nature a humidity ‘background’ may be influenced by leaves and other factors in the habitat, we believe that considering flowers in isolation is sufficient for considering whether there is variation across species. Furthermore, it is useful for detection of fine-scaled patterns in the first place. Whether these are masked by particular natural backgrounds in humidity is a valid question that also needs to be studied. Here we asked whether there is variation in floral humidity production between species, in terms of humidity intensity and patterns produced, and whether the humidity generated by these flowers might be biologically meaningful to pollinating insects through presenting information about the flower.

Our control measurements detected negligible amounts of humidity extraneous to the flower, yet importantly most species (30 of 42) produced humidity of an intensity that exceeded these (i.e., compared to the control TWL in Table 5). Different species showed a wide range of floral humidity intensities (Table 5), ranging from apparently background levels (or comparable to humidity extraneous to the flower), to those producing much more (with $\Delta \,R\,H_{x}^{m\,a\,x}$ reaching as high as 3.71% in giant bindweed *Calystegia silvatica*). Varying levels of floral humidity between species may mean that humidity could provide sensory information that pollinators could not only use to determine the presence and location of rewards on a flower, but also potentially facilitate the discrimination between different flower types. The differing floral humidity may reflect different humidity demands on pollen viability, such as the presence of desiccation resistant pollen that may mitigate the need to elevate floral humidity (Nepi et al., 2001). Differences in floral humidity production may also indicate differing thermoregulation strategies of flower species or contribute to differing susceptibility to fungal pathogens between species. The intensity of floral humidity did not appear to be determined by flower size (span) or type (single flower or inflorescence) when phylogeny was taken into account (Table 6). This finding indicates that shape and other morphological and physiological traits of a species determine its capacity to produce floral humidity. Because both nectar evaporation and floral transpiration potentially contribute to floral humidity generation (von Arx et al., 2012), the most likely candidates for such traits relate to floral nectar and petal permeability.

Nectar-related traits may determine variation in floral humidity between species. Nectar volume present in the flower will determine the level of nectar evaporation (von Arx et al., 2012). Although it was not possible for us to measure nectar volume in our samples without disrupting their humidity production, it is most likely a major source of variation between species. Information about the liquid volume of nectar standing crop, and its variation between species is not available, with most published information being based on dry sugar mass (Baude et al., 2016; Hicks et al., 2016), and may not represent the possibly dynamic nature of humidity production. Nectar volume can differ between species as can patterns of nectar secretion (Biernaskie and Cartar, 2004; Carlson and Harms, 2006; Mačukanovič-Jocič et al. et al., 2004; Keasar et al., 2008) and reabsorption (Langenberger and Davis, 2002). Some flower species differ in the sugar concentration (Corbet, 2003; Brandenburg et al., 2009), types of sugars (Percival, 1961; Corbet et al., 1979b) and the amount of secondary metabolites that are secreted into the nectar (Baker, 1977; Kessler and Baldwin, 2007; Wright et al., 2013; Richardson et al., 2015). These influence the viscosity and concentration of dissolved material which influences evaporation (Corbet et al., 1979b).

As evaporation takes place at the exposed surface of liquids, factors that influence the exposure of liquid nectar to the environment will influence the extent of nectar evaporation (Corbet, 2003), subject to weather conditions (Corbet, 1990; Lawson and Rands, 2019). Thus, aspects of the flower structure (beyond the general form) can also play a part in nectar evaporation. Exposed nectaries and open floral architecture allow increased nectar evaporation (Corbet et al., 1979a; Plowright, 1987; Corbet, 2003). Deep, narrow corollas and nectaries capable of closing can limit evaporation for the same reasons (Plowright, 1987; Corbet, 2003). However, open floral architecture may not be sufficiently shielded (von Arx et al., 2012), preventing humidity from accumulating, while deep corollas create an enclosed space that may allow areas of high humidity to accumulate (Corbet et al., 1979b; von Arx et al., 2012; von Arx, 2013). It is possible that nectary exposure may have been altered in some species with normally downward facing flowers, such as *Fuchsia* and *Abutilon* × *milleri* hort., when they were reoriented for sampling, possibly explaining these species lower humidity intensities. Further studies separating the effects of these different factors in nectar production should be conducted across specifically selected ranges of closely related or polymorphic species.

Differences in petal permeability between species could also lead to variation in floral humidity production. Transpirational water loss can occur directly through the petal cuticle (Patiño and Grace, 2002; Buschhaus et al., 2015; Guo et al., 2017). Petals often have more permeable cuticles than leaves, allowing greater water loss (von Arx et al., 2012; Buschhaus et al., 2015). Variation in the rate of transpirational water loss can be due to differences in the chemical composition of the cuticle (Corbet et al., 1979b; Schreiber and Riederer, 1996; Goodwin et al., 2003; Guo et al., 2017) and cuticle thickness (Hajibagheri et al., 1983). Many flower species have floral stomata, although at a lower density to leaves (Baker, 1977; Davies et al., 2005; Hew et al., 1980; Inamdar, 1968; Shah and Gopal, 1971; von Arx et al., 2012). Stomata have a major influence on transpirational water loss from plant tissues (Baker, 1977; Jarvis and McNaughton, 1986). While floral stomata appeared to be absent among many of the highest humidity producers surveyed in the subsample of the dataset (Table 5), floral stomata vary in their gaseous exchange and transpiration activity between species. In some flowers floral stomata carry out a similar levels of transpiration to leaf stomata (Huang et al., 2018; von Arx et al., 2012), while in others floral stomata have been observed to be non-functioning (Hew et al., 1980; van Doorn, 1997). The presence, density, location, functionality and opening patterns of floral stomata are therefore likely to influence floral humidity generation.

Flower structure may influence floral humidity generation by transpiration in a similar way to influencing nectar evaporation by creating enclosed spaces and buffer zones allowing humidity to accumulate (Corbet et al., 1979a, b; Corbet, 2003). Understanding how variation in nectar, transpirational, and flower structure traits relate to floral humidity production may start to explain this variation in floral humidity signal intensity observed in the transects presented here, and we suggest that further surveys considering the presence and arrangement of petal stomata would be enlightening.

The structure of floral humidity patterns was reasonably consistent, usually showing a quadratic *x* axis and decreasing *z* axis relationship (Table 5). Such humidity structures are similar to that observed by von Arx et al. (2012). There were species that differed from this structure, however: particularly in the *z* axis where humidity was found frequently to be level across the 30 mm transect. Many of the flowers with flat humidity models in the *z* axis produced lower intensity floral humidity (Table 5). A best-fitting model with a flat structure may therefore reflect a lack of humidity differences generated by these flower species, resulting in humidity differences remaining constant but at a low intensity across the transect. The small amount of humidity produced by *Fuchsia* could similarly explain why it showed a flat humidity structure in the *x* axis. However, an atypical humidity structure was not always correlated with species that produce little humidity. *Calystegia silvatica* produced the highest measured floral humidity intensity and showed a linear *x* axis structure. *C. silvatica* was among the largest flowers sampled, with a flower span slightly exceeding the *x* axis transect width (Table 5). Consequently, only the zenith of a quadratic structure was probably sampled in the transect, potentially explaining the atypical humidity structure of *C. silvatica*.

Even when species showed a similar shaped humidity structure, they still differed in the broadness and shape of these humidity structures (differences in *B*_*x*_ and *b*_*x*_ shown in Appendix S4 in Supplementary File 1), as demonstrated in Figures 4, 5. The highest predicted value of floral humidity $X_{t}^{m\,a\,x}$ tended to be near a flower’s center, corresponding to the usual location of nectaries in radially symmetrical flowers. This supports the association between nectar and floral humidity (von Arx et al., 2012). Differences seen in humidity structure shape and the location of $X_{t}^{m\,a\,x}$ may be due variation in positioning and orientation of nectaries or of nectar-producing florets within inflorescences. Such variation may influence where vapor accumulates in the flower headspace, influencing the structure of floral humidity. Alternatively, transpiration may differ across the flower surface due to location of petal stomata and differences in cuticle thickness and composition, similarly altering humidity structure between species. The differences in flower geometry and complexity across the flower alone may explain differences in floral humidity structure. More complex or vertically arranged floral structures may have a higher surface area relative to the area of the flower headspace they contribute to, resulting in such regions of the flower headspace (all else being equal) receiving more transpiration. Changes in flower geometry and complexity may also influence how humidity enters the floral headspace by creating differences in boundary layer effects across the flower surface potentially trapping vapor or increasing its accumulation.

The transects used in this survey only sampled partial cross-sections of the whole flower headspace (Figure 3) as opposed to all the three-dimensional space above the flower. Although the full three-dimensional shape of floral humidity was not sampled, these transects still found structural differences in humidity (Table 5). More complex humidity sampling procedures may reveal further complexity and diversity in floral humidity structure across flower species. ‘More complex’ sampling procedures may include additional measurements offset in the *y* axis, relative to the transect central point (i.e., including sampling points in the lateral plain to the left and right in the robot co-ordination system, see axes indicated in Figure 3A), or include measurements offset in multiple axes (*x*, *y*, and *z*) at once. Such procedures would sample the floral headspace more completely and create more three-dimensional measurements of floral humidity structure. Our models assumed a degree symmetry in *x* axis humidity structure as all species sampled showed a high degree of radial symmetry. For the same reason we did not carry out a *y* axis transect. Our survey suggests radially symmetrical flowers generally show symmetry in their floral humidity, given $X_{t}^{m\,a\,x}$ is central and the *x* axis humidity structure is quadratic in most species (Table 5). However, $X_{t}^{m\,a\,x}$ was not always central, suggesting there may be some differences in symmetry of floral humidity structure between species. Such differences may be more clearly revealed by these complex sampling procedures, particularly those species that offset from the flower center in different directions (such as the *y* axis). Similarly, more complex sampling procedures could be applied to investigate the occurrence of floral humidity in flower species that do not show radial symmetry. It would be expected that such differences in floral shape related to floral symmetry would lead to differences in floral humidity structure compared to radially symmetrical flowers, particularly the symmetry of floral humidity.

The measurements we made of *O. caespitosa* were not too dissimilar from those reported by von Arx et al. (2012), whose humidity signals had an average peak *x* axis humidity signal of approximately 4% (our comparable $\Delta \,R\,H_{x}^{m\,a\,x}$ was 1.79%). Contrary to their observation where humidity dropped to zero over time, we did not find this in our repeated sequences. The small variation in *O. caespitosa* floral humidity between studies cannot be explained by temperature and humidity conditions, as they were similar to ours. The reduced humidity that we observed may be due to *O. caespitosa* being a nocturnal plant surveyed here in daytime. All the flowers we used were freshly cut from the plant while von Arx et al. (2012) allowed them to remain attached, though isolated them using a sealed 46 cm^3^ box. It is possible that the cutting applied here disrupted the transpirational or evaporative processes of *O. caespitosa* (van Doorn, 1997), resulting in lower humidity production (although many cut flowers function normally following cutting within the timescales of our sampling: Fanourakis et al., 2012; Huang et al., 2018; Lü et al., 2011). It could be that the robot arm used here allows for more careful control for air disturbance than the continually moving mechanically operated screws used to control probe movement in the previous study. The robot was able to move slowly with precision but also able to pause during transects, allowing waiting time for floral humidity to stabilize after disruption from probe motion before humidity measurements. This resulted in the high measurement repeatability and small turbulence effects seen here (Table 4). Alternatively, the sealed box used in von Arx et al. (2012) may have allowed humidity to accumulate to higher than normal levels during their sampling. Because the robot can move autonomously we worked in a larger room (as opposed to a sealed 46 cm^3^ box). This would allow floral transpiration and nectar evaporation to occur in a manner more similar to how it would in an open environment, perhaps allowing a more natural humidity equilibrium to be reached. Furthermore, the arm was also capable of more complex motion, allowing us to conduct continuous probe control measurements throughout the experiment, reducing variation as a result of differences between probes.

## Conclusion

Our survey of floral humidity across different plant species has revealed that floral humidity can be detected in many species. Our transects showed that floral humidity intensity and humidity structure can vary between species, although the reasons behind this variation remain unclear. In this way, our transects reveal floral humidity to show a greater level of diversity and complexity than previously known. This wider occurrence of floral humidity could enhance our knowledge of plant-pollinator signaling, as floral humidity is a known cue for at least one pollinating species (von Arx et al., 2012). Similarly, the wider occurrence of floral humidity warrants further exploration of the links between floral humidity and flower thermoregulation, pollen viability and susceptibility to disease.

Pollinator responses to between species differences in structural arrangement or patterns of floral signals have been observed in visual (Goodale et al., 2014; Hempel de Ibarra et al., 2015), olfactory (Lawson et al., 2017, 2018), electrostatic (Clarke et al., 2013) and thermal (Harrap et al., 2017, 2019) flower signals. It would be of interest to investigate how much further different species vary in humidity structure, as well as whether pollinators that use floral humidity, such as hawkmoths *Hyles lineata* (von Arx et al., 2012), respond to the variations in humidity contrasts but also structure independent of its intensity in the flower’s headspace.

## Data Availability Statement

All data generated or analyzed during this study are included in Supplementary File 2.

## Author Contributions

MH contributed to study conceptualization, robot transect design, robot operation, floral humidity data curation and analysis, and writing (wrote original manuscript, review and editing). NH contributed to study conceptualization, funding acquisition, and writing (review and editing). HK carried out robot programming and initial operation. HW contributed to study conceptualization, funding acquisition, and writing (review and editing). SR contributed to study conceptualization, funding acquisition, carried out phylogenetic analyses, and writing (drafting, review, and editing).

## Conflict of Interest

The authors declare that the research was conducted in the absence of any commercial or financial relationships that could be construed as a potential conflict of interest.

## Supplementary Material

SUPPLEMENTARY FILE 1 Includes the following appendices. (Appendix S1) AIC tables and sampling dates of flower species floral humidity analyses. (Appendix S2) AIC tables and sampling dates of control tube humidity analyses. (Appendix S3) Derivation of Eq. (12). (Appendix S4) The parameter values of the best-fitting humidity structure models. Also includes, (Figure S1) A scale floorplan of the lab where humidity signal sampling took place.

SUPPLEMENTARY FILE 2 A zipped file containing floral humidity data collected by the robot arm transects as described in the main text. A word document within the zipped files explains the data formatting in detail. Datafiles themselves are in CSV format.

SUPPLEMENTARY FILE 3 A zipped file containing floral humidity structures for the flower species and controls sampled within the study as described in the main text. A word document within the zipped files explains the data set in detail.

SUPPLEMENTARY VIDEO 1 A video demonstrating the full sequence of probe motion made by the robot within the full humidity transect sampling procedure. In this video, the arm is moving at a greater speed then it would during actual sampling and not pausing to take measurements, consequentially it completes the full movement cycle quickly. Here, the robot moves at 10% nominal speed, during sampling the robot move would move at 3% nominal speed. Here, the arm does not pause for measurements, instead coming to a complete stop where it would take measurements before resuming movement. The individual flowers shown in the video are to demonstrate the location of transects and measurements but were not used in sampling. Supplementary image 1 provides a guide for identifying objects in the image. Note the video has had audio removed.

SUPPLEMENTARY IMAGE 1 A labeled frame of Supplementary Video 1 which provides a guide for identifying objects in the video. In addition to the robot, the locations of the focal probe and background probe are indicated, as are the locations of their respective *Arduino UNO* microcomputers. However, the background probe microcomputer is obscured by the wooden mount supporting the background probe. Flower positions are indicated by letters ‘a,’ ‘b,’ ‘c,’ and ‘d’ as in Figure 3A, indicating the randomly selected sampling order chosen by the robot in this sequence.
