# Supplementary material for: Floral Humidity in Flowering Plants: A Preliminary Survey
Source: Front Plant Sci. 2020 Mar 6;11:249. doi: 10.3389/fpls.2020.00249 (PMC7068853; doi:10.3389/fpls.2020.00249)

**Floral humidity in flowering plants - Harrap et al.**

**SUPPLEMENTARY FILE 1**

**Appendix S1: AIC tables and sampling dates of flower species floral humidity analyses**

For each individual of each species the date and time at which the first x axis transect replicate began is given (YYYY-MM-DD-hh-mm-ss). In each AIC table, AIC and degrees of freedom ‘df’ are given. Difference in ΔAIC, here calculated as AIC of model with the lowest AIC minus that of the current model, is also provided. Within each AIC table, shaded and in bold are the best fitting models as per the guidelines given in Richards (2008).

| *Abutilon* x *milleri* hort*.* | | | | | | | | |
| --- | --- | --- | --- | --- | --- | --- | --- | --- |
| X axis model | df | AIC | ΔAIC |  | Z axis model | df | AIC | ΔAIC |
| **m6** | **7** | **-134.30** | **0.00** |  | **z2** | **6** | **-39.16** | **0.00** |
| m9 | 10 | -132.48 | -1.83 |  | z3 | 7 | -37.37 | -1.79 |
| m7 | 8 | -132.33 | -1.97 |  | z4 | 10 | -31.86 | -7.30 |
| m10 | 14 | -127.38 | -6.93 |  | z0 | 3 | -30.41 | -8.75 |
| m2 | 4 | -109.86 | -24.45 |  | z1 | 4 | -28.60 | -10.56 |
| m3 | 5 | -107.89 | -26.42 |  |  | |  | |
| m4 | 6 | -93.36 | -40.94 |  | Sampling dates: | | 2017-10-11-12-14-25 | |
| m5 | 7 | -91.39 | -42.92 |  |  | | 2017-10-12-14-11-55 | |
| m8 | 10 | -87.84 | -46.46 |  |  | | 2017-10-17-12-26-17 | |
| m0 | 3 | -72.73 | -61.57 |  |  | | 2017-10-25-12-03-34 | |
| m1 | 4 | -70.76 | -63.55 |  |  | | 2018-06-21-14-48-05 | |
|  |  |  |  |  |  | | 2018-07-04-15-11-38 | |
|  | | | | | | | | |

| *Achillea millefolium* | | | |  |  | | | |
| --- | --- | --- | --- | --- | --- | --- | --- | --- |
| X axis model | df | AIC | ΔAIC |  | Z axis model | df | AIC | ΔAIC |
| m7 | 8 | 560.46 | 0.00 |  | z3 | 7 | -109.95 | 0.00 |
| **m3** | **5** | **564.58** | **-4.12** |  | z4 | 10 | -106.58 | -3.37 |
| m10 | 14 | 565.05 | -4.59 |  | **z1** | **4** | **-104.12** | **-5.84** |
| m6 | 7 | 576.52 | -16.06 |  | z2 | 6 | -73.85 | -36.10 |
| m9 | 10 | 576.99 | -16.53 |  | z0 | 3 | -70.78 | -39.17 |
| m2 | 4 | 580.07 | -19.61 |  |  | |  |  |
| m1 | 4 | 831.78 | -271.32 |  | Sampling dates | | 2018-07-19-10-28-34 | |
| m5 | 7 | 833.62 | -273.16 |  |  | | 2018-07-19-11-46-03 | |
| m0 | 3 | 837.15 | -276.70 |  |  | | 2018-07-20-10-03-55 | |
| m8 | 10 | 839.00 | -278.54 |  |  | | 2018-07-20-12-38-48 | |
| m4 | 6 | 839.09 | -278.64 |  |  |  | 2018-07-23-14-00-20 | |
|  |  |  |  |  |  |  | 2018-07-23-15-17-50 | |
|  | | | | | | | | |
| *Allium ursinum* | | | |  |  | | | |
| X axis model | df | AIC | ΔAIC |  | Z axis model | df | AIC | ΔAIC |
| m3 | 5 | -98.78 | 0.00 |  | z3 | 7 | -93.15 | 0.00 |
| m4 | 6 | -96.81 | -1.97 |  | **z2** | **6** | **-93.04** | **-0.11** |
| m8 | 10 | -96.34 | -2.44 |  | z4 | 10 | -88.08 | -5.07 |
| **m2** | **4** | **-93.67** | **-5.11** |  | z0 | 3 | -42.94 | -50.21 |
| m1 | 4 | -38.05 | -60.73 |  | z1 | 4 | -42.11 | -51.04 |
| m0 | 3 | -34.77 | -64.01 |  |  |  |  |  |
| m5 | 7 | 102.18 | -200.96 |  | Sampling dates | | 2018-05-31-12-45-26 | |
| m9 | 10 | 179.73 | -278.51 |  |  |  | 2018-06-01-14-41-55 | |
| m10 | 14 | 183.55 | -282.33 |  |  |  | 2018-06-04-10-53-20 | |
| m6 | 7 | 185.13 | -283.91 |  |  |  | 2018-06-05-13-02-36 | |
| m7 | 8 | 194.66 | -293.44 |  |  |  |  | |
|  |  |  |  |  |  |  |  | |

|  |
| --- |

| *Bellis perennis* | | | |  |  | | | |
| --- | --- | --- | --- | --- | --- | --- | --- | --- |
| X axis model | df | AIC | ΔAIC |  | Z axis model | df | AIC | ΔAIC |
| **m2** | **4** | **-157.19** | **0.00** |  | **z3** | **7** | **-448.07** | **0.00** |
| m3 | 5 | -155.38 | -1.81 |  | z4 | 10 | -445.11 | -2.96 |
| m9 | 10 | -154.87 | -2.33 |  | z2 | 6 | -425.75 | -22.32 |
| m6 | 7 | -154.59 | -2.61 |  | z1 | 4 | -372.01 | -76.05 |
| m7 | 8 | -152.78 | -4.42 |  | z0 | 3 | -360.07 | -88.00 |
| m10 | 14 | -147.95 | -9.25 |  |  |  |  |  |
| m0 | 3 | 136.20 | -293.40 |  | Sampling dates | | 2018-05-03-10-02-36 | |
| m1 | 4 | 138.13 | -295.32 |  |  | | 2018-05-03-12-37-33 | |
| m4 | 6 | 140.91 | -298.11 |  |  |  | 2018-05-03-13-54-58 | |
| m5 | 7 | 142.84 | -300.04 |  |  |  | 2018-05-09-10-28-54 | |
| m8 | 10 | 148.51 | -305.71 |  |  |  | 2018-05-09-11-46-21 | |
|  |  |  |  |  |  |  | 2018-05-21-10-13-25 | |
|  | | | | | | | | |
| *Calystegia silvatica* | | | |  |  | | | |
| X axis model | df | AIC | ΔAIC |  | Z axis model | df | AIC | ΔAIC |
| m10 | 14 | 1105.23 | 0.00 |  | **z1** | **4** | **495.56** | **0.00** |
| **m8** | **10** | **1105.77** | **-0.55** |  | z3 | 7 | 500.63 | -5.07 |
| m7 | 8 | 1116.62 | -11.39 |  | z4 | 10 | 503.99 | -8.43 |
| m5 | 7 | 1119.70 | -14.47 |  | z0 | 3 | 535.30 | -39.74 |
| m3 | 5 | 1126.50 | -21.27 |  | z2 | 6 | 540.61 | -45.05 |
| m1 | 4 | 1129.33 | -24.10 |  |  |  |  |  |
| m6 | 7 | 1195.07 | -89.84 |  | Sampling dates | | 2018-06-25-11-11-10 | |
| m4 | 6 | 1196.99 | -91.76 |  |  |  | 2018-06-28-11-29-34 | |
| m9 | 10 | 1198.83 | -93.61 |  |  |  | 2018-06-29-13-34-47 | |
| m2 | 4 | 1201.35 | -96.13 |  |  |  | 2018-07-02-13-42-12 | |
| m0 | 3 | 1203.12 | -97.89 |  |  |  | 2018-07-03-15-40-32 | |
|  |  |  |  |  |  |  | 2018-07-06-10-28-25 | |
|  | | | | | | | | |

| *Campanula* sp. | | | | |  |  | | | | |
| --- | --- | --- | --- | --- | --- | --- | --- | --- | --- | --- |
| X axis model | df | AIC | ΔAIC | |  | Z axis model | df | AIC | | ΔAIC |
| m3 | 5 | -267.34 | 0.00 | |  | **z2** | **6** | **-371.83** | | **0.00** |
| m7 | 8 | -267.30 | -0.04 | |  | z3 | 7 | -371.73 | | -0.10 |
| **m2** | **4** | **-262.39** | **-4.96** | |  | z4 | 10 | -365.82 | | -6.01 |
| m6 | 7 | -262.21 | -5.13 | |  | z0 | 3 | -351.18 | | -20.65 |
| m9 | 10 | -256.64 | -10.70 | |  | z1 | 4 | -350.74 | | -21.09 |
| m10 | 14 | -255.87 | -11.47 | |  |  |  |  | |  |
| m1 | 4 | -47.02 | -220.32 | |  | Sampling dates | | 2018-08-01-10-22-14 | | |
| m0 | 3 | -45.64 | -221.71 | |  |  |  | 2018-08-01-12-57-07 | | |
| m5 | 7 | -43.89 | -223.45 | |  |  |  | 2018-08-01-11-39-41 | | |
| m4 | 6 | -42.47 | -224.87 | |  |  | | 2018-08-02-11-05-06 | | |
| m8 | 10 | -37.95 | -229.39 | |  |  |  | 2018-08-02-12-22-31 | | |
|  |  |  |  | |  |  |  | 2018-08-03-15-08-04 | | |
|  | | | | | | | | | | |
| *Cyanus montanus* | | | | |  |  | | | | |
| X axis model | df | AIC | | ΔAIC |  | Z axis model | df | AIC | ΔAIC | |
| **m7** | **8** | **-342.32** | | **0.00** |  | **z2** | **6** | **-202.33** | **0.00** | |
| m10 | 14 | -337.73 | | -4.59 |  | z3 | 7 | -201.05 | -1.28 | |
| m9 | 10 | -333.98 | | -8.34 |  | z4 | 10 | -195.66 | -6.67 | |
| m6 | 7 | -333.14 | | -9.19 |  | z0 | 3 | -189.42 | -12.91 | |
| m3 | 5 | -314.36 | | -27.96 |  | z1 | 4 | -188.06 | -14.27 | |
| m2 | 4 | -306.33 | | -35.99 |  |  |  |  |  | |
| m5 | 7 | -177.40 | | -164.92 |  | Sampling dates | | 2018-06-25-15-03-29 | | |
| m4 | 6 | -172.86 | | -169.46 |  |  |  | 2018-06-28-10-12-11 | | |
| m8 | 10 | -171.57 | | -170.75 |  |  |  | 2018-06-29-10-59-52 | | |
| m1 | 4 | -163.26 | | -179.06 |  |  |  | 2018-07-02-14-59-35 | | |
| m0 | 3 | -159.14 | | -183.18 |  |  |  | 2018-07-03-14-23-09 | | |
|  |  |  | |  |  |  |  | 2018-07-06-11-45-50 | | |
|  | | | | | | | | | | |

| *Cyanus segetum* | | | |  |  | | | |
| --- | --- | --- | --- | --- | --- | --- | --- | --- |
| X axis model | df | AIC | ΔAIC |  | Z axis model | df | AIC | ΔAIC |
| **m6** | **7** | **587.29** | **0.00** |  | **z0** | **3** | **5.29** | **0.00** |
| m9 | 10 | 587.69 | -0.40 |  | z1 | 4 | 5.78 | -0.50 |
| m7 | 8 | 589.16 | -1.87 |  | z2 | 6 | 8.77 | -3.48 |
| m10 | 14 | 594.92 | -7.63 |  | z3 | 7 | 9.24 | -3.95 |
| m2 | 4 | 595.90 | -8.61 |  | z4 | 10 | 14.26 | -8.97 |
| m3 | 5 | 597.77 | -10.48 |  |  |  |  |  |
| m4 | 6 | 676.59 | -89.30 |  | Sampling dates | | 2018-05-31-15-20-22 | |
| m5 | 7 | 678.50 | -91.21 |  |  |  | 2018-06-01-10-49-30 | |
| m0 | 3 | 681.50 | -94.21 |  |  |  | 2018-06-04-12-10-45 | |
| m1 | 4 | 683.40 | -96.12 |  |  |  | 2018-06-05-09-10-11 | |
| m8 | 10 | 684.03 | -96.74 |  |  |  | 2018-06-05-10-27-38 | |
|  |  |  |  |  |  |  | 2018-06-18-14-28-24 | |
|  | | | | | | | | |
| *Cistus* 'greyswood pink' | | | |  |  | | | |
| X axis model | df | AIC | ΔAIC |  | Z axis model | df | AIC | ΔAIC |
| m3 | 5 | 172.88 | 0.00 |  | **z3** | **7** | **-257.35** | **0.00** |
| m7 | 8 | 173.30 | -0.42 |  | z4 | 10 | -256.01 | -1.34 |
| **m2** | **4** | **175.22** | **-2.34** |  | z1 | 4 | -237.68 | -19.67 |
| m6 | 7 | 175.71 | -2.84 |  | z2 | 6 | -230.01 | -27.34 |
| m9 | 10 | 179.56 | -6.68 |  | z0 | 3 | -214.90 | -42.45 |
| m10 | 14 | 182.49 | -9.61 |  |  |  |  |  |
| m1 | 4 | 331.19 | -158.31 |  | Sampling dates | | 2018-05-11-11-50-45 | |
| m0 | 3 | 331.77 | -158.89 |  |  | | 2018-05-11-14-25-43 | |
| m5 | 7 | 333.90 | -161.02 |  |  |  | 2018-05-14-14-17-28 | |
| m4 | 6 | 334.50 | -161.63 |  |  |  | 2018-05-14-13-00-01 | |
| m8 | 10 | 339.54 | -166.66 |  |  |  | 2018-05-15-10-26-31 | |
|  |  |  |  |  |  |  | 2018-05-15-13-01-23 | |
|  | | | | | | | | |

| *Clematis chinensis* | | | |  |  | | | |
| --- | --- | --- | --- | --- | --- | --- | --- | --- |
| X axis model | df | AIC | ΔAIC |  | Z axis model | df | AIC | ΔAIC |
| m10 | 14 | 100.74 | 0.00 |  | **z2** | **6** | **113.79** | **0.00** |
| **m7** | **8** | **100.98** | **-0.24** |  | z3 | 7 | 114.92 | -1.13 |
| m6 | 7 | 112.24 | -11.50 |  | z4 | 10 | 120.68 | -6.90 |
| m9 | 10 | 112.34 | -11.61 |  | z0 | 3 | 129.12 | -15.33 |
| m3 | 5 | 124.24 | -23.51 |  | z1 | 4 | 130.43 | -16.64 |
| m2 | 4 | 133.78 | -33.05 |  |  |  |  |  |
| m5 | 7 | 187.58 | -86.84 |  | Sampling dates | | 2017-10-11-14-49-10 | |
| m8 | 10 | 189.87 | -89.13 |  |  |  | 2017-10-12-15-29-18 | |
| m4 | 6 | 194.27 | -93.53 |  |  |  | 2017-10-17-15-01-02 | |
| m1 | 4 | 201.01 | -100.27 |  |  |  | 2017-10-25-14-38-21 | |
| m0 | 3 | 206.92 | -106.18 |  |  |  |  | |
|  |  |  |  |  |  |  |  | |
|  | | | | | | | | |
| *Convolvulus sabatius* | | | |  |  | | | |
| X axis model | df | AIC | ΔAIC |  | Z axis model | df | AIC | ΔAIC |
| **m9** | **10** | **83.92** | **0.00** |  | z4 | 10 | -32.22 | 0.00 |
| m10 | 14 | 86.46 | -2.53 |  | **z3** | **7** | **-29.94** | **-2.27** |
| m6 | 7 | 108.10 | -24.17 |  | z2 | 6 | -5.68 | -26.53 |
| m7 | 8 | 110.10 | -26.17 |  | z1 | 4 | 2.97 | -35.18 |
| m2 | 4 | 193.23 | -109.30 |  | z0 | 3 | 21.22 | -53.44 |
| m3 | 5 | 195.23 | -111.30 |  |  |  |  |  |
| m4 | 6 | 257.84 | -173.91 |  | Sampling dates | | 2017-10-11-13-31-47 | |
| m5 | 7 | 259.84 | -175.91 |  |  | | 2017-10-12-12-54-32 | |
| m8 | 10 | 262.83 | -178.91 |  |  |  | 2017-10-17-13-43-38 | |
| m0 | 3 | 310.49 | -226.57 |  |  |  | 2017-10-25-15-55-46 | |
| m1 | 4 | 312.49 | -228.57 |  |  |  | 2018-06-21-10-55-44 | |
|  |  |  |  |  |  |  | 2018-07-04-11-19-20 | |
|  | | | | | | | | |

| *Coreopsis* sp*.* | | | |  |  | | | |
| --- | --- | --- | --- | --- | --- | --- | --- | --- |
| X axis model | df | AIC | ΔAIC |  | Z axis model | df | AIC | ΔAIC |
| m7 | 8 | -140.37 | 0.00 |  | **z3** | **7** | **-78.13** | **0.00** |
| m10 | 14 | -137.87 | -2.51 |  | z4 | 10 | -72.56 | -5.57 |
| m9 | 10 | -137.00 | -3.37 |  | z2 | 6 | -71.68 | -6.44 |
| m6 | 7 | -135.84 | -4.53 |  | z1 | 4 | -58.49 | -19.64 |
| **m3** | **5** | **-135.80** | **-4.58** |  | z0 | 3 | -53.44 | -24.69 |
| m2 | 4 | -131.49 | -8.89 |  |  |  |  |  |
| m1 | 4 | 158.99 | -299.37 |  | Sampling dates | | 2017-09-18-11-41-06 | |
| m0 | 3 | 159.40 | -299.77 |  |  | | 2017-09-19-12-03-28 | |
| m5 | 7 | 161.02 | -301.40 |  |  |  | 2017-09-20-11-53-45 | |
| m4 | 6 | 161.46 | -301.84 |  |  |  | 2017-09-26-13-45-40 | |
| m8 | 10 | 166.24 | -306.61 |  |  |  | 2017-09-27-10-37-20 | |
|  |  |  |  |  |  |  | 2017-11-22-12-32-03 | |
|  | | | | | | | | |
| *Cosmos bipinnatus* | | | |  |  | | | |
| X axis model | df | AIC | ΔAIC |  | Z axis model | df | AIC | ΔAIC |
| **m7** | **8** | **189.04** | **0.00** |  | z3 | 7 | -131.70 | 0.00 |
| m3 | 5 | 196.26 | -7.21 |  | **z1** | **4** | **-129.25** | **-2.46** |
| m6 | 7 | 197.61 | -8.57 |  | z4 | 10 | -126.66 | -5.05 |
| m10 | 14 | 199.86 | -10.82 |  | z2 | 6 | -116.37 | -15.34 |
| m9 | 10 | 202.74 | -13.70 |  | z0 | 3 | -114.88 | -16.82 |
| m2 | 4 | 204.39 | -15.34 |  |  |  |  |  |
| m5 | 7 | 260.35 | -71.30 |  | Sampling dates | | 2017-07-18-12-32-34 | |
| m1 | 4 | 264.79 | -75.75 |  |  |  | 2017-07-19-10-13-07 | |
| m8 | 10 | 266.12 | -77.08 |  |  |  | 2017-07-20-16-35-40 | |
| m4 | 6 | 266.69 | -77.65 |  |  |  | 2017-07-24-13-25-34 | |
| m0 | 3 | 270.86 | -81.82 |  |  |  | 2017-07-25-15-34-23 | |
|  |  |  |  |  |  |  | 2017-08-09-13-28-00 | |
|  | | | | | | | | |

| *Epilobium hirsutum* | | | |  |  | | | |
| --- | --- | --- | --- | --- | --- | --- | --- | --- |
| X axis model | df | AIC | ΔAIC |  | Z axis model | df | AIC | ΔAIC |
| m3 | 5 | 228.15 | 0.00 |  | **z2** | **6** | **-55.78** | **0.00** |
| **m2** | **4** | **228.45** | **-0.30** |  | z3 | 7 | -55.50 | -0.28 |
| m7 | 8 | 232.70 | -4.55 |  | z4 | 10 | -49.67 | -6.12 |
| m6 | 7 | 233.00 | -4.85 |  | z0 | 3 | -42.31 | -13.47 |
| m9 | 10 | 234.97 | -6.82 |  | z1 | 4 | -41.80 | -13.98 |
| m10 | 14 | 239.85 | -11.70 |  |  |  |  |  |
| m0 | 3 | 365.60 | -137.45 |  | Sampling dates | | 2018-07-09-15-44-44 | |
| m1 | 4 | 366.14 | -137.99 |  |  |  | 2018-07-10-09-58-00 | |
| m4 | 6 | 370.68 | -142.53 |  |  |  | 2018-07-10-12-32-52 | |
| m5 | 7 | 371.22 | -143.07 |  |  |  | 2018-07-13-10-08-58 | |
| m8 | 10 | 376.74 | -148.59 |  |  |  | 2018-07-13-11-26-23 | |
|  |  |  |  |  |  |  | 2018-07-16-12-50-14 | |
|  | | | | | | | | |
| *Eschscholzia californica* | | | |  |  | | | |
| X axis model | df | AIC | ΔAIC |  | Z axis model | df | AIC | ΔAIC |
| **m7** | **8** | **1394.98** | **0.00** |  | **z4** | **10** | **513.73** | **0.00** |
| m10 | 14 | 1395.98 | -1.00 |  | z3 | 7 | 524.82 | -11.09 |
| m8 | 10 | 1400.17 | -5.19 |  | z1 | 4 | 539.47 | -25.73 |
| m3 | 5 | 1402.16 | -7.18 |  | z2 | 6 | 577.74 | -64.01 |
| m5 | 7 | 1404.65 | -9.67 |  | z0 | 3 | 585.94 | -72.21 |
| m1 | 4 | 1411.35 | -16.37 |  |  |  |  |  |
| m6 | 7 | 1463.97 | -68.99 |  | Sampling dates | | 2018-05-31-11-27-59 | |
| m2 | 4 | 1468.47 | -73.49 |  |  |  | 2018-06-01-12-06-57 | |
| m9 | 10 | 1469.89 | -74.91 |  |  |  | 2018-06-04-13-28-12 | |
| m4 | 6 | 1471.26 | -76.29 |  |  |  | 2018-06-15-09-58-53 | |
| m0 | 3 | 1475.45 | -80.47 |  |  | | 2018-06-15-12-33-51 | |
|  |  |  |  |  |  |  | 2018-06-18-10-36-01 | |
|  | | | | | | | | |

| *Euphorbia millii* | | | |  |  | | | |
| --- | --- | --- | --- | --- | --- | --- | --- | --- |
| X axis model | df | AIC | ΔAIC |  | Z axis model | df | AIC | ΔAIC |
| **m6** | **7** | **60.38** | **0.00** |  | **z2** | **6** | **33.13** | **0.00** |
| m7 | 8 | 62.29 | -1.90 |  | z3a | 7 | 35.09 | -1.96 |
| m9 | 10 | 66.24 | -5.86 |  | z4a | 10 | 40.74 | -7.62 |
| m10 | 14 | 72.82 | -12.44 |  | z0 | 3 | 41.70 | -8.57 |
| m2 | 4 | 80.56 | -20.18 |  | z1a | 4 | 43.66 | -10.54 |
| m4 | 6 | 81.63 | -21.25 |  |  |  |  |  |
| m3 | 5 | 82.47 | -22.09 |  | Sampling dates | | 2017-11-22-13-49-26 | |
| m5 | 7 | 83.54 | -23.16 |  |  | | 2017-11-22-16-24-17 | |
| m8 | 10 | 88.31 | -27.93 |  |  |  | 2017-11-23-10-59-28 | |
| m0 | 3 | 99.97 | -39.58 |  |  |  | 2017-11-23-13-34-18 | |
| m1 | 4 | 101.88 | -41.50 |  |  |  | 2017-11-27-12-31-06 | |
|  |  |  |  |  |  |  | 2017-11-27-13-48-27 | |
|  | | | | | | | | |
| *Fuchsia* sp. | | | |  |  | | | |
| X axis model | df | AIC | ΔAIC |  | Z axis model | df | AIC | ΔAIC |
| m2 | 4 | -52.52 | 0.00 |  | z3 | 7 | -156.31 | 0.00 |
| **m0** | **3** | **-50.88** | **-1.64** |  | **z2** | **6** | **-153.66** | **-2.65** |
| m3 | 5 | -50.60 | -1.93 |  | z4 | 10 | -152.58 | -3.73 |
| m1 | 4 | -48.96 | -3.57 |  | z1 | 4 | -111.30 | -45.00 |
| m10 | 14 | 197.82 | -250.35 |  | z0 | 3 | -110.07 | -46.24 |
| m8 | 10 | 198.27 | -250.79 |  |  |  |  |  |
| m5 | 7 | 202.92 | -255.44 |  | Sampling dates | | 2018-07-10-13-50-21 | |
| m9 | 10 | 204.31 | -256.84 |  |  |  | 2018-07-16-11-32-50 | |
| m4 | 6 | 204.79 | -257.32 |  |  |  | 2018-07-16-14-07-41 | |
| m7 | 8 | 207.08 | -259.61 |  |  |  | 2018-07-17-10-28-02 | |
| m6 | 7 | 208.96 | -261.48 |  |  | | 2018-07-17-11-45-27 | |
|  |  |  |  |  |  | | 2018-07-17-13-02-53 | |
|  | | | | | | | | |

| *Geranium* ‘Roxanne’ | | | |  |  | | | |
| --- | --- | --- | --- | --- | --- | --- | --- | --- |
| X axis model | df | AIC | ΔAIC |  | Z axis model | df | AIC | ΔAIC |
| **m7** | **8** | **-108.97** | **0.00** |  | z4 | 10 | -235.40 | 0.00 |
| m10 | 14 | -100.40 | -8.57 |  | **z3** | **7** | **-232.76** | **-2.64** |
| m6 | 7 | -99.10 | -9.87 |  | z1 | 4 | -210.06 | -25.34 |
| m9 | 10 | -94.55 | -14.41 |  | z2 | 6 | -199.12 | -36.28 |
| m3 | 5 | -79.43 | -29.54 |  | z0 | 3 | -182.45 | -52.96 |
| m2 | 4 | -70.84 | -38.13 |  |  |  |  |  |
| m5 | 7 | 98.86 | -207.83 |  | Sampling dates | | 2017-09-18-14-15-54 | |
| m4 | 6 | 102.90 | -211.86 |  |  |  | 2017-09-19-10-46-02 | |
| m8 | 10 | 103.90 | -212.87 |  |  |  | 2017-09-26-15-03-03 | |
| m1 | 4 | 111.28 | -220.24 |  |  |  | 2017-09-27-13-12-08 | |
| m0 | 3 | 114.96 | -223.93 |  |  |  | 2017-09-28-12-13-11 | |
|  |  |  |  |  |  |  | 2018-05-16-10-52-19 | |
|  | | | | | | | | |
| *Geranium robertianum* | | | |  |  | | | |
| X axis model | df | AIC | ΔAIC |  | Z axis model | df | AIC | ΔAIC |
| **m2** | **4** | **286.70** | **0.00** |  | **z2** | **6** | **-207.72** | **0.00** |
| m3 | 5 | 288.69 | -1.99 |  | z3 | 7 | -206.08 | -1.64 |
| m9 | 10 | 290.32 | -3.62 |  | z4 | 10 | -202.65 | -5.07 |
| m6 | 7 | 292.53 | -5.83 |  | z0 | 3 | -195.47 | -12.25 |
| m7 | 8 | 294.52 | -7.82 |  | z1 | 4 | -193.79 | -13.93 |
| m10 | 14 | 296.59 | -9.89 |  |  |  |  |  |
| m0 | 3 | 354.70 | -68.00 |  | Sampling dates | | 2018-06-25-13-46-00 | |
| m1 | 4 | 356.69 | -69.99 |  |  |  | 2018-06-28-14-04-32 | |
| m4 | 6 | 360.57 | -73.87 |  |  |  | 2018-06-29-14-52-12 | |
| m5 | 7 | 362.56 | -75.86 |  |  |  | 2018-07-02-11-07-14 | |
| m8 | 10 | 367.22 | -80.52 |  |  |  | 2018-07-03-11-48-13 | |
|  |  |  |  |  |  |  | 2018-07-06-13-03-17 | |
|  | | | | | | | | |

| *Geranium sanguineum* | | | |  |  | | | |
| --- | --- | --- | --- | --- | --- | --- | --- | --- |
| X axis model | df | AIC | ΔAIC |  | Z axis model | df | AIC | ΔAIC |
| **m9** | **10** | **69.36** | **0.00** |  | **z3** | **7** | **-187.40** | **0.00** |
| m10 | 14 | 74.68 | -5.31 |  | z4 | 10 | -184.64 | -2.77 |
| m6 | 7 | 80.61 | -11.24 |  | z2 | 6 | -178.00 | -9.41 |
| m7 | 8 | 82.45 | -13.08 |  | z1 | 4 | -126.40 | -61.00 |
| m2 | 4 | 109.45 | -40.08 |  | z0 | 3 | -121.27 | -66.13 |
| m3 | 5 | 111.31 | -41.94 |  |  |  |  |  |
| m4 | 6 | 254.58 | -185.22 |  | Sampling dates | | 2018-06-28-12-47-03 | |
| m5 | 7 | 256.49 | -187.13 |  |  |  | 2018-06-29-12-17-19 | |
| m8 | 10 | 261.16 | -191.79 |  |  | | 2018-07-02-12-24-43 | |
| m0 | 3 | 268.67 | -199.31 |  |  |  | 2018-07-03-13-05-40 | |
| m1 | 4 | 270.59 | -201.22 |  |  |  | 2018-07-06-14-20-47 | |
|  |  |  |  |  |  |  | 2018-07-09-11-52-24 | |
|  | | | | | | | | |
| *Lantana* sp. | | | |  |  | | | |
| X axis model | df | AIC | ΔAIC |  | Z axis model | df | AIC | ΔAIC |
| **m2** | **4** | **475.45** | **0.00** |  | z3 | 7 | -115.16 | 0.00 |
| m3 | 5 | 477.15 | -1.69 |  | **z1** | **4** | **-113.42** | **-1.74** |
| m6 | 7 | 479.41 | -3.95 |  | z4 | 10 | -110.60 | -4.56 |
| m7 | 8 | 481.10 | -5.64 |  | z0 | 3 | -80.24 | -34.92 |
| m9 | 10 | 483.35 | -7.90 |  | z2 | 6 | -80.20 | -34.96 |
| m10 | 14 | 490.95 | -15.49 |  |  |  |  |  |
| m0 | 3 | 730.76 | -255.31 |  | Sampling dates | | 2017-07-18-16-24-44 | |
| m1 | 4 | 732.63 | -257.17 |  |  |  | 2017-07-19-14-05-17 | |
| m4 | 6 | 735.88 | -260.42 |  |  |  | 2017-07-20-14-00-54 | |
| m5 | 7 | 737.75 | -262.29 |  |  |  | 2017-07-24-14-42-55 | |
| m8 | 10 | 743.71 | -268.25 |  |  |  | 2017-07-25-11-40-20 | |
|  |  |  |  |  |  |  | 2017-08-09-12-10-35 | |
|  | | | | | | | | |

| *Lavandula angustifolia* | | | |  |  | | | |
| --- | --- | --- | --- | --- | --- | --- | --- | --- |
| X axis model | df | AIC | ΔAIC |  | Z axis model | df | AIC | ΔAIC |
| m7 | 8 | 34.35 | 0.00 |  | z3 | 7 | -73.62 | 0.00 |
| **m6** | **7** | **38.65** | **-4.30** |  | **z2** | **6** | **-70.93** | **-2.69** |
| m10 | 14 | 40.51 | -6.16 |  | z4 | 10 | -68.52 | -5.10 |
| m9 | 10 | 42.99 | -8.64 |  | z1 | 4 | -42.00 | -31.62 |
| m3 | 5 | 70.06 | -35.71 |  | z0 | 3 | -40.42 | -33.20 |
| m2 | 4 | 73.56 | -39.21 |  |  |  |  |  |
| m5 | 7 | 216.61 | -182.26 |  | Sampling dates | | 2018-07-27-11-31-18 | |
| m4 | 6 | 218.08 | -183.73 |  |  | | 2018-07-27-15-23-41 | |
| m8 | 10 | 220.36 | -186.01 |  |  |  | 2018-07-30-10-40-46 | |
| m1 | 4 | 234.15 | -199.80 |  |  |  | 2018-07-30-13-15-40 | |
| m0 | 3 | 235.36 | -201.01 |  |  |  | 2018-07-31-10-21-43 | |
|  |  |  |  |  |  |  | 2018-07-31-11-39-07 | |
|  | | | | | | | | |
| *Leucanthemum vulgare* | | | |  |  | | | |
| X axis model | df | AIC | ΔAIC |  | Z axis model | df | AIC | ΔAIC |
| m10 | 14 | 402.76 | 0.00 |  | **z4** | **10** | **-120.65** | **0.00** |
| **m7** | **8** | **408.59** | **-5.84** |  | z3 | 7 | -112.30 | -8.36 |
| m9 | 10 | 420.41 | -17.65 |  | z2 | 6 | -80.97 | -39.68 |
| m3 | 5 | 423.98 | -21.23 |  | z1 | 4 | -18.36 | -102.29 |
| m6 | 7 | 428.29 | -25.54 |  | z0 | 3 | -5.20 | -115.46 |
| m2 | 4 | 442.01 | -39.25 |  |  |  |  |  |
| m5 | 7 | 618.49 | -215.73 |  | Sampling dates | | 2018-07-27-12-48-43 | |
| m1 | 4 | 622.03 | -219.27 |  |  |  | 2018-07-27-14-06-12 | |
| m8 | 10 | 623.39 | -220.63 |  |  |  | 2018-07-30-11-58-13 | |
| m4 | 6 | 626.17 | -223.41 |  |  | | 2018-07-30-14-33-05 | |
| m0 | 3 | 629.36 | -226.60 |  |  |  | 2018-07-31-12-56-32 | |
|  |  |  |  |  |  |  |  | |
|  | | | | | | | | |

| *Lilium* sp. | | | |  |  | | | |
| --- | --- | --- | --- | --- | --- | --- | --- | --- |
| X axis model | df | AIC | ΔAIC |  | Z axis model | df | AIC | ΔAIC |
| m10 | 14 | 429.76 | 0.00 |  | z3 | 7 | -211.08 | 0.00 |
| **m8** | **10** | **431.30** | **-1.53** |  | **z2** | **6** | **-210.26** | **-0.81** |
| m9 | 10 | 440.01 | -10.25 |  | z4 | 10 | -207.27 | -3.81 |
| m4 | 6 | 441.00 | -11.24 |  | z1 | 4 | -187.57 | -23.51 |
| m0 | 3 | 441.92 | -12.16 |  | z0 | 3 | -187.30 | -23.78 |
| m5 | 7 | 442.80 | -13.03 |  |  |  |  |  |
| m6 | 7 | 443.00 | -13.23 |  | Sampling dates | | 2017-11-22-15-06-50 | |
| m1 | 4 | 443.72 | -13.96 |  |  |  | 2017-11-23-12-16-53 | |
| m2 | 4 | 443.92 | -14.15 |  |  |  | 2017-11-23-14-51-41 | |
| m7 | 8 | 444.79 | -15.03 |  |  |  | 2017-11-27-11-13-41 | |
| m3 | 5 | 445.72 | -15.95 |  |  | | 2018-04-09-13-13-01 | |
|  |  |  |  |  |  |  | 2018-04-13-11-42-49 | |
|  | | | | | | | | |
| *Linum grandiflorum* | | | |  |  | | | |
| X axis model | df | AIC | ΔAIC |  | Z axis model | df | AIC | ΔAIC |
| **m10** | **14** | **-222.80** | **0.00** |  | z4 | 10 | -239.47 | 0.00 |
| m7 | 8 | -208.48 | -14.32 |  | **z3** | **7** | **-238.41** | **-1.06** |
| m6 | 7 | -206.13 | -16.68 |  | z2 | 6 | -230.82 | -8.65 |
| m9 | 10 | -201.15 | -21.66 |  | z1 | 4 | -182.46 | -57.01 |
| m3 | 5 | -159.53 | -63.27 |  | z0 | 3 | -178.26 | -61.21 |
| m2 | 4 | -157.89 | -64.92 |  |  |  |  |  |
| m8 | 10 | -140.56 | -82.25 |  | Sampling dates | | 2017-09-18-10-23-43 | |
| m5 | 7 | -127.53 | -95.27 |  |  |  | 2017-09-19-13-20-49 | |
| m4 | 6 | -126.20 | -96.60 |  |  |  | 2017-09-20-10-36-22 | |
| m1 | 4 | -90.76 | -132.04 |  |  |  | 2017-09-26-12-28-17 | |
| m0 | 3 | -89.87 | -132.94 |  |  |  | 2017-09-27-14-29-31 | |
|  |  |  |  |  |  |  | 2017-09-28-13-30-35 | |
|  | | | | | | | | |

| *Linum usitatissimum* | | | |  |  | | | |
| --- | --- | --- | --- | --- | --- | --- | --- | --- |
| X axis model | df | AIC | ΔAIC |  | Z axis model | df | AIC | ΔAIC |
| m10 | 14 | 146.18 | 0.00 |  | z3 | 7 | -161.84 | 0.00 |
| **m9** | **10** | **147.48** | **-1.31** |  | z4 | 10 | -161.44 | -0.40 |
| m6 | 7 | 170.30 | -24.12 |  | **z2** | **6** | **-160.41** | **-1.43** |
| m7 | 8 | 171.55 | -25.38 |  | z1 | 4 | -137.78 | -24.06 |
| m2 | 4 | 189.73 | -43.55 |  | z0 | 3 | -137.01 | -24.82 |
| m3 | 5 | 191.04 | -44.87 |  |  |  |  |  |
| m4 | 6 | 340.82 | -194.64 |  | Sampling dates | | 2018-06-21-13-30-34 | |
| m5 | 7 | 342.40 | -196.22 |  |  |  | 2018-06-26-10-35-17 | |
| m8 | 10 | 344.03 | -197.86 |  |  |  | 2018-06-26-11-52-44 | |
| m0 | 3 | 349.55 | -203.37 |  |  |  | 2018-06-26-13-10-11 | |
| m1 | 4 | 351.14 | -204.97 |  |  | | 2018-06-26-14-27-36 | |
|  |  |  |  |  |  |  | 2018-07-04-12-36-47 | |
|  | | | | | | | | |
| *Nepenthes* sp. | | | |  |  | | | |
| X axis model | df | AIC | ΔAIC |  | Z axis model | df | AIC | ΔAIC |
| m6 | 7 | -640.32 | 0.00 |  | **z2** | **6** | **-398.69** | **0.00** |
| m7 | 8 | -638.61 | -1.71 |  | z3 | 7 | -397.04 | -1.65 |
| m9 | 10 | -637.05 | -3.28 |  | z4 | 10 | -395.02 | -3.67 |
| **m2** | **4** | **-636.65** | **-3.67** |  | z0 | 3 | -389.03 | -9.66 |
| m10 | 14 | -636.25 | -4.08 |  | z1 | 4 | -387.34 | -11.35 |
| m3 | 5 | -634.94 | -5.39 |  |  |  |  |  |
| m0 | 3 | -360.19 | -280.13 |  | Sampling dates | | 2018-05-04-10-39-51 | |
| m1 | 4 | -358.31 | -282.02 |  |  | | 2018-05-04-11-57-21 | |
| m4 | 6 | -358.05 | -282.28 |  |  |  | 2018-05-04-13-14-50 | |
| m5 | 7 | -356.16 | -284.16 |  |  |  | 2018-05-04-14-32-17 | |
| m8 | 10 | -352.85 | -287.48 |  |  |  | 2018-05-10-11-38-47 | |
|  |  |  |  |  |  |  | 2018-05-10-14-13-44 | |
|  | | | | | | | | |

| *Nicotiana tabacum* | | | |  |  | | | |
| --- | --- | --- | --- | --- | --- | --- | --- | --- |
| X axis model | df | AIC | ΔAIC |  | Z axis model | df | AIC | ΔAIC |
| **m2** | **4** | **-940.22** | **0.00** |  | z3 | 7 | -550.60 | 0.00 |
| m3 | 5 | -938.23 | -1.99 |  | z4 | 10 | -547.78 | -2.82 |
| m6 | 7 | -938.03 | -2.20 |  | **z2** | **6** | **-545.19** | **-5.42** |
| m7 | 8 | -936.03 | -4.19 |  | z1 | 4 | -538.71 | -11.89 |
| m9 | 10 | -934.91 | -5.31 |  | z0 | 3 | -534.18 | -16.43 |
| m10 | 14 | -928.70 | -11.52 |  |  |  |  |  |
| m0 | 3 | -847.95 | -92.27 |  | Sampling dates | | 2017-11-29-11-24-12 | |
| m1 | 4 | -845.96 | -94.26 |  |  |  | 2017-11-29-12-41-33 | |
| m4 | 6 | -844.75 | -95.48 |  |  | | 2017-11-29-13-58-56 | |
| m5 | 7 | -842.75 | -97.47 |  |  | | 2017-11-29-15-16-23 | |
| m8 | 10 | -838.04 | -102.18 |  |  |  | 2018-04-09-11-55-32 | |
|  |  |  |  |  |  |  | 2018-04-09-15-47-53 | |
|  | | | | | | | | |
| *Oenothera caespitosa* | | | |  |  | | | |
| X axis model | df | AIC | ΔAIC |  | Z axis model | df | AIC | ΔAIC |
| **m10** | **14** | **452.49** | **0.00** |  | **z1** | **4** | **14.05** | **0.00** |
| m7 | 8 | 461.04 | -8.55 |  | z3 | 7 | 16.56 | -2.52 |
| m9 | 10 | 485.11 | -32.62 |  | z4 | 10 | 21.60 | -7.56 |
| m3 | 5 | 491.56 | -39.07 |  | z0 | 3 | 54.55 | -40.50 |
| m6 | 7 | 497.05 | -44.56 |  | z2 | 6 | 58.00 | -43.95 |
| m2 | 4 | 523.53 | -71.04 |  |  |  |  |  |
| m5 | 7 | 617.95 | -165.46 |  | Sampling dates | | 2018-08-02-13-39-59 | |
| m8 | 10 | 623.86 | -171.37 |  |  | | 2018-08-02-14-57-28 | |
| m1 | 4 | 634.20 | -181.71 |  |  |  | 2018-08-03-11-15-43 | |
| m4 | 6 | 639.13 | -186.64 |  |  |  | 2018-08-03-12-33-08 | |
| m0 | 3 | 653.81 | -201.32 |  |  |  | 2018-08-03-13-50-37 | |
|  |  |  |  |  |  |  | 2018-08-06-11-38-32 | |
|  | | | | | | | | |

| *Osteospermum* sp. | | | | |  |  | | | | |
| --- | --- | --- | --- | --- | --- | --- | --- | --- | --- | --- |
| X axis model | df | AIC | | ΔAIC |  | Z axis model | df | AIC | ΔAIC | |
| **m10** | **14** | **329.77** | | **0.00** |  | **z4** | **10** | **-37.56** | **0.00** | |
| m7 | 8 | 345.02 | | -15.25 |  | z3 | 7 | -4.89 | -32.67 | |
| m9 | 10 | 375.93 | | -46.16 |  | z1 | 4 | 26.37 | -63.92 | |
| m6 | 7 | 378.77 | | -49.00 |  | z2 | 6 | 76.34 | -113.90 | |
| m3 | 5 | 388.25 | | -58.48 |  | z0 | 3 | 92.20 | -129.75 | |
| m2 | 4 | 416.95 | | -87.17 |  |  |  |  |  | |
| m8 | 10 | 478.53 | | -148.76 |  | Sampling dates | | 2017-07-18-13-49-59 | | |
| m5 | 7 | 483.01 | | -153.23 |  |  |  | 2017-07-19-11-30-31 | | |
| m4 | 6 | 504.11 | | -174.34 |  |  |  | 2017-07-20-15-18-15 | | |
| m1 | 4 | 509.07 | | -179.30 |  |  |  | 2017-07-24-12-08-11 | | |
| m0 | 3 | 527.96 | | -198.18 |  |  |  | 2017-07-25-14-16-22 | | |
|  |  |  | |  |  |  |  | 2017-08-09-14-45-23 | | |
|  | | | | | | | | | | |
| *Papaver cambricum* | | | | |  |  | | | | |
| X axis model | df | AIC | ΔAIC | |  | Z axis model | df | AIC | | ΔAIC |
| m7 | 8 | 768.48 | 0.00 | |  | **z1** | **4** | **-144.20** | | **0.00** |
| m3 | 5 | 768.88 | -0.39 | |  | z3 | 7 | -139.67 | | -4.53 |
| m6 | 7 | 772.41 | -3.93 | |  | z4 | 10 | -137.02 | | -7.17 |
| **m2** | **4** | **772.68** | **-4.20** | |  | z0 | 3 | -135.33 | | -8.86 |
| m9 | 10 | 773.47 | -4.99 | |  | z2 | 6 | -130.69 | | -13.50 |
| m10 | 14 | 773.62 | -5.14 | |  |  |  |  | |  |
| m5 | 7 | 784.35 | -15.87 | |  | Sampling dates | | 2018-05-09-13-03-46 | | |
| m1 | 4 | 784.38 | -15.90 | |  |  |  | 2018-05-09-14-21-15 | | |
| m0 | 3 | 787.87 | -19.38 | |  |  | | 2018-05-10-12-56-15 | | |
| m4 | 6 | 787.94 | -19.46 | |  |  |  | 2018-05-10-15-31-09 | | |
| m8 | 10 | 788.66 | -20.17 | |  |  |  | 2018-05-11-10-33-18 | | |
|  |  |  |  | |  |  |  | 2018-05-11-13-08-14 | | |
|  | | | | | | | | | | |

| *Papaver rhoeas* | | | |  |  | | | |
| --- | --- | --- | --- | --- | --- | --- | --- | --- |
| X axis model | df | AIC | ΔAIC |  | Z axis model | df | AIC | ΔAIC |
| m5 | 7 | -99.49 | 0.00 |  | z3 | 7 | -164.02 | 0.00 |
| **m3** | **5** | **-98.00** | **-1.49** |  | z4 | 10 | -159.72 | -4.31 |
| m8 | 10 | -97.86 | -1.64 |  | **z2** | **6** | **-158.94** | **-5.09** |
| m4 | 6 | -82.76 | -16.73 |  | z1 | 4 | -142.71 | -21.31 |
| m2 | 4 | -81.59 | -17.90 |  | z0 | 3 | -138.87 | -25.15 |
| m1 | 4 | -37.87 | -61.62 |  |  |  |  |  |
| m0 | 3 | -24.76 | -74.73 |  | Sampling dates | | 2018-06-15-13-51-16 | |
| m6 | 7 | 155.07 | -254.56 |  |  |  | 2018-06-18-13-10-59 | |
| m9 | 10 | 168.27 | -267.76 |  |  | | 2018-06-19-11-27-20 | |
| m7 | 8 | 177.15 | -276.65 |  |  | | 2018-06-19-12-44-49 | |
| m10 | 14 | 192.06 | -291.55 |  |  |  | 2018-06-19-14-02-18 | |
|  |  |  |  |  |  |  | 2018-06-19-15-19-43 | |
|  | | | | | | | | |
| *Potentilla* sp. | | | |  |  | | | |
| X axis model | df | AIC | ΔAIC |  | Z axis model | df | AIC | ΔAIC |
| **m7** | **8** | **-207.04** | **0.00** |  | **z1** | **4** | **-329.74** | **0.00** |
| m10 | 14 | -204.01 | -3.03 |  | z3 | 7 | -326.48 | -3.26 |
| m3 | 5 | -197.37 | -9.66 |  | z4 | 10 | -321.83 | -7.91 |
| m9 | 10 | -195.64 | -11.40 |  | z0 | 3 | -317.09 | -12.65 |
| m6 | 7 | -193.91 | -13.13 |  | z2 | 6 | -313.55 | -16.19 |
| m2 | 4 | -184.98 | -22.06 |  |  |  |  |  |
| m1 | 4 | 155.86 | -362.90 |  | Sampling dates | | 2018-05-14-10-25-04 | |
| m5 | 7 | 157.04 | -364.08 |  |  |  | 2018-05-14-11-42-32 | |
| m0 | 3 | 158.44 | -365.48 |  |  |  | 2018-05-15-11-43-56 | |
| m4 | 6 | 159.69 | -366.73 |  |  |  | 2018-05-15-14-18-52 | |
| m8 | 10 | 162.79 | -369.83 |  |  | | 2018-05-16-12-09-48 | |
|  |  |  |  |  |  |  | 2018-05-16-13-27-15 | |
|  | | | | | | | | |

| *Ranunculus* *acris* | | | |  |  | | | |
| --- | --- | --- | --- | --- | --- | --- | --- | --- |
| X axis model | df | AIC | ΔAIC |  | Z axis model | df | AIC | ΔAIC |
| **m9** | **10** | **1128.43** | **0.00** |  | z4 | 10 | 157.87 | 0.00 |
| m10 | 14 | 1130.99 | -2.56 |  | **z3** | **7** | **163.43** | **-5.55** |
| m7 | 8 | 1139.72 | -11.29 |  | z1 | 4 | 165.57 | -7.70 |
| m6 | 7 | 1142.25 | -13.82 |  | z0 | 3 | 209.32 | -51.45 |
| m3 | 5 | 1158.13 | -29.70 |  | z2 | 6 | 209.52 | -51.65 |
| m2 | 4 | 1160.32 | -31.88 |  |  |  |  |  |
| m5 | 7 | 1265.50 | -137.06 |  | Sampling dates | | 2018-04-30-10-20-20 | |
| m4 | 6 | 1266.49 | -138.05 |  |  |  | 2018-04-30-11-37-45 | |
| m8 | 10 | 1271.12 | -142.69 |  |  |  | 2018-04-30-12-55-14 | |
| m1 | 4 | 1275.79 | -147.36 |  |  | | 2018-04-30-14-12-42 | |
| m0 | 3 | 1276.63 | -148.19 |  |  |  | 2018-05-01-10-05-37 | |
|  |  |  |  |  |  |  | 2018-05-03-11-20-04 | |
|  | | | | | | | | |
| *Ranunculus lingua* | | | |  |  | | | |
| X axis model | df | AIC | ΔAIC |  | Z axis model | df | AIC | ΔAIC |
| m10 | 14 | 915.79 | 0.00 |  | z1 | 4 | 13.05 | 0.00 |
| **m7** | **8** | **921.71** | **-5.91** |  | z4 | 10 | 16.28 | -3.23 |
| m3 | 5 | 925.22 | -9.43 |  | **z3** | **7** | **17.87** | **-4.83** |
| m9 | 10 | 946.93 | -31.14 |  | z0 | 3 | 103.37 | -90.32 |
| m6 | 7 | 951.34 | -35.55 |  | z2 | 6 | 108.77 | -95.72 |
| m2 | 4 | 953.93 | -38.14 |  |  |  |  |  |
| m1 | 4 | 1145.70 | -229.91 |  | Sampling dates | | 2018-07-09-13-09-53 | |
| m5 | 7 | 1147.14 | -231.35 |  |  |  | 2018-07-09-14-27-20 | |
| m8 | 10 | 1150.25 | -234.46 |  |  | | 2018-07-10-11-15-27 | |
| m0 | 3 | 1158.93 | -243.14 |  |  |  | 2018-07-13-12-43-50 | |
| m4 | 6 | 1160.59 | -244.80 |  |  |  | 2018-07-13-14-01-21 | |
|  |  |  |  |  |  |  | 2018-07-16-10-15-21 | |
|  | | | | | | | | |

| *Rudbeckia hirta* | | | |  |  | | | |
| --- | --- | --- | --- | --- | --- | --- | --- | --- |
| X axis model | df | AIC | ΔAIC |  | Z axis model | df | AIC | ΔAIC |
| **m7** | **8** | **655.28** | **0.00** |  | z3 | 7 | -51.52 | 0.00 |
| m10 | 14 | 663.03 | -7.75 |  | z4 | 10 | -49.12 | -2.39 |
| m6 | 7 | 664.14 | -8.86 |  | **z1** | **4** | **-46.23** | **-5.29** |
| m3 | 5 | 665.04 | -9.75 |  | z2 | 6 | -30.94 | -20.58 |
| m9 | 10 | 668.20 | -12.92 |  | z0 | 3 | -27.30 | -24.22 |
| m2 | 4 | 673.36 | -18.07 |  |  |  |  |  |
| m5 | 7 | 729.08 | -73.79 |  | Sampling dates | | 2017-09-18-12-58-29 | |
| m8 | 10 | 733.34 | -78.06 |  |  |  | 2017-09-19-14-38-14 | |
| m1 | 4 | 735.44 | -80.16 |  |  |  | 2017-09-20-13-11-08 | |
| m4 | 6 | 735.59 | -80.30 |  |  |  | 2017-09-26-11-10-54 | |
| m0 | 3 | 741.62 | -86.34 |  |  |  | 2017-09-27-11-54-43 | |
|  |  |  |  |  |  |  | 2017-09-28-10-55-46 | |
|  | | | | | | | | |
| *Scabiosa* sp. | | | |  |  | | | |
| X axis model | df | AIC | ΔAIC |  | Z axis model | df | AIC | ΔAIC |
| **m3** | **5** | **251.20** | **0.00** |  | z3 | 7 | -75.78 | 0.00 |
| m7 | 8 | 255.72 | -4.52 |  | **z1** | **4** | **-70.03** | **-5.76** |
| m10 | 14 | 264.58 | -13.39 |  | z4 | 10 | -69.80 | -5.98 |
| m2 | 4 | 270.29 | -19.09 |  | z2 | 6 | -26.77 | -49.01 |
| m6 | 7 | 274.91 | -23.71 |  | z0 | 3 | -24.54 | -51.24 |
| m9 | 10 | 279.69 | -28.49 |  |  |  |  |  |
| m1 | 4 | 572.77 | -321.57 |  | Sampling dates | | 2017-07-18-15-07-20 | |
| m5 | 7 | 578.26 | -327.06 |  |  |  | 2017-07-19-12-47-54 | |
| m0 | 3 | 578.26 | -327.07 |  |  |  | 2017-07-20-12-43-29 | |
| m8 | 10 | 583.63 | -332.43 |  |  |  | 2017-07-24-16-00-20 | |
| m4 | 6 | 583.76 | -332.57 |  |  |  | 2017-07-25-12-58-21 | |
|  |  |  |  |  |  |  | 2017-08-09-10-53-12 | |
|  | | | | | | | | |

| *Taraxacum* agg. | | | |  |  | | | |
| --- | --- | --- | --- | --- | --- | --- | --- | --- |
| X axis model | df | AIC | ΔAIC |  | Z axis model | df | AIC | ΔAIC |
| m10 | 14 | 803.67 | 0.00 |  | **z4** | **10** | **68.66** | **0.00** |
| **m9** | **10** | **808.22** | **-4.55** |  | z3 | 7 | 109.50 | -40.83 |
| m7 | 8 | 829.61 | -25.94 |  | z1 | 4 | 133.79 | -65.13 |
| m6 | 7 | 835.27 | -31.60 |  | z2 | 6 | 166.51 | -97.85 |
| m3 | 5 | 887.53 | -83.86 |  | z0 | 3 | 181.00 | -112.34 |
| m2 | 4 | 891.76 | -88.09 |  |  |  |  |  |
| m5 | 7 | 1064.85 | -261.18 |  | Sampling dates | | 2018-04-26-10-58-07 | |
| m4 | 6 | 1066.40 | -262.73 |  |  | | 2018-04-26-12-15-36 | |
| m8 | 10 | 1069.21 | -265.54 |  |  |  | 2018-04-26-14-50-30 | |
| m1 | 4 | 1089.96 | -286.29 |  |  |  | 2018-05-01-11-23-06 | |
| m0 | 3 | 1091.16 | -287.49 |  |  |  | 2018-05-01-12-40-30 | |
|  |  |  |  |  |  |  | 2018-05-01-13-57-55 | |
|  | | | | | | | | |
| *Trifolium pratense* | | | |  |  | | | |
| X axis model | df | AIC | ΔAIC |  | Z axis model | df | AIC | ΔAIC |
| **m9** | **10** | **-227.50** | **0.00** |  | z3 | 7 | -212.80 | 0.00 |
| m10 | 14 | -221.32 | -6.18 |  | **z2** | **6** | **-212.25** | **-0.55** |
| m6 | 7 | -220.33 | -7.17 |  | z4 | 10 | -207.13 | -5.67 |
| m7 | 8 | -219.52 | -7.98 |  | z0 | 3 | -177.36 | -35.44 |
| m2 | 4 | -139.87 | -87.63 |  | z1 | 4 | -177.26 | -35.55 |
| m3 | 5 | -138.77 | -88.73 |  |  |  |  |  |
| m4 | 6 | 33.85 | -261.35 |  | Sampling dates | | 2018-07-19-13-03-30 | |
| m5 | 7 | 35.33 | -262.83 |  |  | | 2018-07-19-14-20-55 | |
| m8 | 10 | 41.09 | -268.59 |  |  |  | 2018-07-20-11-21-23 | |
| m0 | 3 | 68.86 | -296.37 |  |  |  | 2018-07-20-13-56-15 | |
| m1 | 4 | 70.40 | -297.91 |  |  |  | 2018-07-23-11-25-28 | |
|  |  |  |  |  |  |  | 2018-07-23-12-42-51 | |
|  | | | | | | | | |

| *Tulbaghia violacea* | | | | | |  | |  | | | | | | |
| --- | --- | --- | --- | --- | --- | --- | --- | --- | --- | --- | --- | --- | --- | --- |
| X axis model | df | | AIC | ΔAIC | |  | | Z axis model | | df | | AIC | | ΔAIC |
| **m9** | **10** | | **-42.88** | **0.00** | |  | | **z2** | | **6** | | **-153.87** | | **0.00** |
| m10 | 14 | | -36.69 | -6.19 | |  | | z3 | | 7 | | -153.03 | | -0.84 |
| m6 | 7 | | -35.69 | -7.19 | |  | | z4 | | 10 | | -149.23 | | -4.64 |
| m7 | 8 | | -34.31 | -8.58 | |  | | z0 | | 3 | | -130.12 | | -23.74 |
| m2 | 4 | | 2.47 | -45.35 | |  | | z1 | | 4 | | -129.07 | | -24.81 |
| m3 | 5 | | 3.94 | -46.82 | |  | |  | |  | |  | |  |
| m4 | 6 | | 42.15 | -85.03 | |  | | Sampling dates | | | | 2017-10-11-16-06-35 | | |
| m5 | 7 | | 43.67 | -86.56 | |  | |  | | | | 2017-10-12-11-37-09 | | |
| m8 | 10 | | 48.81 | -91.69 | |  | |  | |  | | 2017-10-17-11-08-54 | | |
| m0 | 3 | | 70.72 | -113.60 | |  | |  | |  | | 2017-10-25-13-20-58 | | |
| m1 | 4 | | 72.30 | -115.18 | |  | |  | |  | | 2018-06-21-12-13-09 | | |
|  |  | |  |  | |  | |  | |  | | 2018-07-04-13-54-13 | | |
|  | | | | | | | | | | | | | | |
| *Vinca herbacea* | | | | | | |  | |  | | | | | |
| X axis model | df | AIC | | | ΔAIC | |  | | Z axis model | | df | | AIC | ΔAIC |
| **m7** | **8** | **-841.56** | | | **0.00** | |  | | z4 | | 10 | | -486.79 | 0.00 |
| m10 | 14 | -838.34 | | | -3.22 | |  | | z2 | | 6 | | -486.39 | -0.40 |
| m3 | 5 | -818.74 | | | -22.82 | |  | | z3 | | 7 | | -484.58 | -2.21 |
| m6 | 7 | -810.45 | | | -31.11 | |  | | **z0** | | **3** | | **-482.43** | **-4.35** |
| m9 | 10 | -809.09 | | | -32.47 | |  | | z1 | | 4 | | -480.62 | -6.17 |
| m2 | 4 | -790.46 | | | -51.10 | |  | |  | |  | |  |  |
| m5 | 7 | -592.50 | | | -249.06 | |  | | Sampling dates | | | | 2018-04-13-13-00-18 | |
| m8 | 10 | -588.05 | | | -253.51 | |  | |  | |  | | 2018-04-13-14-17-41 | |
| m1 | 4 | -585.48 | | | -256.09 | |  | |  | |  | | 2018-04-13-15-35-08 | |
| m4 | 6 | -579.48 | | | -262.08 | |  | |  | | | | 2018-04-25-12-39-36 | |
| m0 | 3 | -573.07 | | | -268.50 | |  | |  | |  | | 2018-04-25-13-57-03 | |
|  |  |  | | |  | |  | |  | |  | | 2018-04-26-13-33-05 | |
|  | | | | | | | | | | | | | | |

| *Xerochrysum bracteatum* | | | |  |  | | | |
| --- | --- | --- | --- | --- | --- | --- | --- | --- |
| X axis model | df | AIC | ΔAIC |  | Z axis model | df | AIC | ΔAIC |
| **m3** | **5** | **1117.21** | **0.00** |  | **z1** | **4** | **4.94** | **0.00** |
| m7 | 8 | 1120.26 | -3.05 |  | z3 | 7 | 6.10 | -1.16 |
| m2 | 4 | 1124.41 | -7.20 |  | z4 | 10 | 10.65 | -5.72 |
| m6 | 7 | 1127.54 | -10.33 |  | z0 | 3 | 90.70 | -85.76 |
| m10 | 14 | 1128.29 | -11.08 |  | z2 | 6 | 94.16 | -89.22 |
| m9 | 10 | 1129.75 | -12.54 |  |  |  |  |  |
| m1 | 4 | 1335.68 | -218.46 |  | Sampling dates | | 2018-05-31-14-02-55 | |
| m0 | 3 | 1338.18 | -220.97 |  |  |  | 2018-06-01-13-24-26 | |
| m5 | 7 | 1340.24 | -223.03 |  |  |  | 2018-06-04-14-45-41 | |
| m4 | 6 | 1342.77 | -225.56 |  |  |  | 2018-06-05-11-45-07 | |
| m8 | 10 | 1346.21 | -229.00 |  |  |  | 2018-06-15-11-16-24 | |
|  |  |  |  |  |  |  | 2018-06-18-11-53-32 | |

**Appendix S2: AIC tables and sampling dates of control tube humidity analyses**

For each individual control the date and time at which the first x axis transect replicate began is given (YYYY-MM-DD-hh-mm-ss). In each AIC table, AIC and degrees of freedom ‘df’ are given. Difference in ΔAIC, here calculated as AIC of model with the lowest AIC minus that of the current model, is also provided. Within each AIC table, shaded and in bold are the best fitting models as per the guidelines given in Richards (2008).

|  | | | | | | | | |
| --- | --- | --- | --- | --- | --- | --- | --- | --- |
| Control TLP (empty tube with lid and putty) | | | |  |  | | | |
| X axis model | df | AIC | ΔAIC |  | Z axis model | df | AIC | ΔAIC |
| m7 | 8 | -450.55 | 0.00 |  | **z2** | **6** | **-192.59** | **0.00** |
| m5 | 7 | -449.11 | -1.44 |  | z3 | 7 | -191.89 | -0.69 |
| m6 | 7 | -448.99 | -1.56 |  | z4 | 10 | -186.75 | -5.84 |
| **m4** | **6** | **-447.59** | **-2.96** |  | z0 | 3 | -169.15 | -23.43 |
| m8 | 10 | -447.32 | -3.23 |  | z1 | 4 | -168.21 | -24.38 |
| m9 | 10 | -443.31 | -7.24 |  |  |  |  |  |
| m10 | 14 | -443.14 | -7.42 |  | Sampling dates | | 2017-08-16-15-38-03 | |
| m3 | 5 | -354.51 | -96.04 |  |  |  | 2018-04-23-15-01-12 | |
| m1 | 4 | -354.04 | -96.51 |  |  |  | 2018-04-23-11-08-51 | |
| m2 | 4 | -353.95 | -96.60 |  |  | | 2018-04-24-11-09-10 | |
| m0 | 3 | -353.51 | -97.05 |  |  |  | 2018-04-24-13-44-02 | |
|  |  |  |  |  |  |  | 2018-04-25-15-14-30 | |
|  | | | | | | | | |

| Control TL (empty tube with lid) | | | |  |  | | | |
| --- | --- | --- | --- | --- | --- | --- | --- | --- |
| X axis model | df | AIC | ΔAIC |  | Z axis model | df | AIC | ΔAIC |
| m7 | 8 | -927.86 | 0.00 |  | z3 | 7 | -380.63 | 0.00 |
| **m5** | **7** | **-922.57** | **-5.30** |  | z4 | 10 | -380.41 | -0.22 |
| m6 | 7 | -920.07 | -7.79 |  | **z2** | **6** | **-377.64** | **-2.99** |
| m8 | 10 | -917.40 | -10.46 |  | z1 | 4 | -373.31 | -7.33 |
| m10 | 14 | -917.18 | -10.69 |  | z0 | 3 | -370.71 | -9.93 |
| m4 | 6 | -914.97 | -12.89 |  |  |  |  |  |
| m9 | 10 | -914.53 | -13.34 |  | Sampling dates | | 2017-08-10-15-18-02 | |
| m3 | 5 | -861.53 | -66.33 |  |  |  | 2017-08-16-14-20-40 | |
| m1 | 4 | -857.56 | -70.30 |  |  |  | 2018-04-05-13-27-41 | |
| m2 | 4 | -855.51 | -72.35 |  |  |  | 2018-04-09-14-30-30 | |
| m0 | 3 | -851.68 | -76.18 |  |  |  | 2018-04-10-11-06-45 | |
|  |  |  |  |  |  |  | 2018-04-11-13-11-22 | |
|  |  |  |  |  |  |  | 2018-04-12-11-45-17 | |
|  | | | | | | | | |
| Control T (empty open tube) | | | |  |  | | | |
| X axis model | df | AIC | ΔAIC |  | Z axis model | df | AIC | ΔAIC |
| **m7** | **8** | **-966.68** | **0.00** |  | z3 | 7 | -480.40 | 0.00 |
| m10 | 14 | -965.88 | -0.80 |  | **z1** | **4** | **-479.29** | **-1.12** |
| m6 | 7 | -959.90 | -6.78 |  | z4 | 10 | -476.64 | -3.76 |
| m9 | 10 | -955.15 | -11.53 |  | z2 | 6 | -474.86 | -5.54 |
| m3 | 5 | -951.44 | -15.24 |  | z0 | 3 | -474.12 | -6.29 |
| m8 | 10 | -948.35 | -18.33 |  |  |  |  |  |
| m2 | 4 | -945.24 | -21.44 |  | Sampling dates | | 2017-08-10-14-00-37 | |
| m5 | 7 | -945.21 | -21.47 |  |  |  | 2018-04-04-15-20-27 | |
| m4 | 6 | -939.07 | -27.61 |  |  |  | 2018-04-05-12-10-17 | |
| m1 | 4 | -931.50 | -35.18 |  |  |  | 2018-04-10-14-59-09 | |
| m0 | 3 | -925.85 | -40.83 |  |  |  | 2018-04-11-10-36-30 | |
|  |  |  |  |  |  |  | 2018-04-12-10-27-54 | |

|  |
| --- |

| Control TWLP (tube with water, lid and putty) | | | | | | |  | |  | | | | | | |
| --- | --- | --- | --- | --- | --- | --- | --- | --- | --- | --- | --- | --- | --- | --- | --- |
| X axis model | df | AIC | | | ΔAIC | |  | | Z axis model | | df | | AIC | | ΔAIC |
| m7 | 8 | -541.52 | | | 0.00 | |  | | z3 | | 7 | | -206.10 | | 0.00 |
| **m5** | **7** | **-538.67** | | | **-2.84** | |  | | **z2** | | **6** | | **-204.41** | | **-1.69** |
| m8 | 10 | -536.90 | | | -4.62 | |  | | z4 | | 10 | | -200.47 | | -5.62 |
| **m6** | **7** | **-535.99** | | | **-5.52** | |  | | z1 | | 4 | | -187.86 | | -18.24 |
| m10 | 14 | -534.00 | | | -7.52 | |  | | z0 | | 3 | | -186.75 | | -19.34 |
| m4 | 6 | -533.27 | | | -8.25 | |  | |  | |  | |  | |  |
| m9 | 10 | -530.17 | | | -11.34 | |  | | Sampling dates | | | | 2017-08-16-13-03-15 | | |
| m3 | 5 | -490.54 | | | -50.97 | |  | |  | |  | | 2018-04-23-13-43-47 | | |
| m1 | 4 | -488.52 | | | -53.00 | |  | |  | | | | 2018-04-23-12-26-20 | | |
| m2 | 4 | -486.28 | | | -55.23 | |  | |  | |  | | 2018-04-24-12-26-35 | | |
| m0 | 3 | -484.34 | | | -57.18 | |  | |  | |  | | 2018-04-24-15-01-31 | | |
|  |  |  | | |  | |  | |  | |  | | 2018-04-25-11-22-09 | | |
|  | | | | | | | | | | | | | | | |
| Control TWL (tube with water and lid) | | | | | |  | |  | | | | | | | |
| X axis model | df | | AIC | ΔAIC | |  | | Z axis model | | df | | AIC | | ΔAIC | |
| m3 | 5 | | -97.88 | 0.00 | |  | | **z0** | | **3** | | **-377.22** | | **0.00** | |
| m7 | 8 | | -95.56 | -2.32 | |  | | z2 | | 6 | | -376.97 | | -0.24 | |
| **m2** | **4** | | **-92.55** | **-5.32** | |  | | z1 | | 4 | | -376.14 | | -1.08 | |
| m6 | 7 | | -90.15 | -7.73 | |  | | z3 | | 7 | | -375.93 | | -1.29 | |
| m10 | 14 | | -87.02 | -10.86 | |  | | z4 | | 10 | | -370.82 | | -6.40 | |
| m9 | 10 | | -85.75 | -12.13 | |  | |  | |  | |  | |  | |
| m1 | 4 | | 96.22 | -194.10 | |  | | Sampling dates | | | | 2017-08-10-17-52-48 | | | |
| m0 | 3 | | 98.10 | -195.98 | |  | |  | | | | 2018-04-04-12-45-36 | | | |
| m5 | 7 | | 100.29 | -198.16 | |  | |  | |  | | 2018-04-05-10-52-48 | | | |
| m4 | 6 | | 102.19 | -200.07 | |  | |  | |  | | 2018-04-10-13-41-41 | | | |
| m8 | 10 | | 105.34 | -203.21 | |  | |  | |  | | 2018-04-11-14-28-51 | | | |
|  |  | |  |  | |  | |  | |  | | 2018-04-12-13-02-45 | | | |
|  | | | | | | | | | | | | | | | |

| Control TW (open tube with water) | | | |  |  | | | |
| --- | --- | --- | --- | --- | --- | --- | --- | --- |
| X axis model | df | AIC | ΔAIC |  | Z axis model | df | AIC | ΔAIC |
| m3 | 5 | 484.15 | 0.00 |  | **z1** | **4** | **-282.72** | **0.00** |
| **m2** | **4** | **485.09** | **-0.94** |  | z3 | 7 | -280.48 | -2.24 |
| m7 | 8 | 488.66 | -4.51 |  | z4 | 10 | -276.03 | -6.69 |
| m6 | 7 | 489.61 | -5.46 |  | z0 | 3 | -204.04 | -78.68 |
| m9 | 10 | 494.89 | -10.74 |  | z2 | 6 | -200.12 | -82.60 |
| m10 | 14 | 499.74 | -15.59 |  |  |  |  |  |
| m0 | 3 | 680.47 | -196.32 |  | Sampling dates | | 2017-08-10-16-35-25 | |
| m1 | 4 | 680.93 | -196.78 |  |  | | 2018-04-04-11-28-05 | |
| m4 | 6 | 685.70 | -201.54 |  |  |  | 2018-04-05-14-45-08 | |
| m5 | 7 | 686.16 | -202.00 |  |  |  | 2018-04-10-12-24-12 | |
| m8 | 10 | 692.06 | -207.90 |  |  |  | 2018-04-11-11-53-57 | |
|  |  |  |  |  |  |  | 2018-04-12-14-20-16 | |

**Appendix S3: Derivation of Equation 12**

In quadratic curves the vertex is also the plane of symmetry. This means that Equation 4 can be reorganised to

$\Delta{RH}_{xnt}=I_{x}+i_{x}+v_{xn}+X\left( \left( A_{x}+a_{x} \right)+\left( \left( B_{x}+b_{x} \right)X \right) \right)$ (A1)

From equation A1 two values of $X$ spaced equally either side of the vertex, two points that mirror each other in the curves’ symmetry, can be identified, where $\Delta{RH}_{xnt}=I_{x}+i_{x}+v_{xn}$. These are when

$X=0$, (A2)

and

$X=-\frac{\left( A_{x}+a_{x} \right)}{\left( B_{x}+b_{x} \right)}$. (A3)

As the curve is symmetrical, the x coordinates of the vertex for the curve described by equations 4 is halfway between these points, this gives equation 12. Consequently, $X_{t}^{max}$ of a species showing a quadratic x axis relationship can be calculated using Equation 12, and the parameter values estimated by the best fitting model for each species’ *x* axis model. Equation 12 is an adaption of the standard formula for the *x* axis coordinates of a quadratic vertex, with the corresponding parameters form the model described in equation 4 substituted in. This equation can also be derived by calculus (instead of the solution based on graphical properties presented here) to give the same answer.

**Appendix S4: The parameter values of the best fitting humidity structure models**

The parameter values of the best fitting models of both x and z axis models from our analysis of humidity structure of the 42 flower species and 6 controls sampled. All values are given in scientific format ($g\cdot Ex=g\cdot{10}^{x}$).

|  | *Abutilon* x *milleri* hort*.* | *Achillea millefolium* | *Allium ursinum* | *Bellis perennis* | *Calystegia silvatica* | *Campanula* sp. | *Cistus '*greyswood pink' | *Clematis chinensis* | *Convolvulus sabatius* | *Coreopsis* sp. | *Cyanus segetum* | *Cyanus montanus* | *Cosmos bipinnatus* | *Epilobium hirsutum* |
| --- | --- | --- | --- | --- | --- | --- | --- | --- | --- | --- | --- | --- | --- | --- |
|  |  |  |  |  |  |  |  |  |  |  |  |  |  |  |
| $I_{x}$ | 3.17  E-01 | 1.72 E+00 | 2.42  E-01 | 5.82 E-01 | 9.74 E-01 | 3.57 E-01 | 6.61 E-01 | 6.54 E-01 | 1.91 E-01 | 7.05 E-01 | 1.10 E+00 | 2.02 E-01 | 6.31 E-01 | 5.87 E-01 |
| $A_{x}$ |  | 7.34 E-03 |  |  | 1.25 E-02 |  |  | -4.01 E-03 |  | 1.44 E-03 |  | -1.37 E-03 | 3.05 E-03 |  |
| $B_{x}$ | -2.32 E-04 | -2.17 E-03 | -3.56  E-04 | -7.41 E-04 |  | -5.01 E-04 | -8.10 E-04 | -6.88 E-04 | -2.49 E-04 | -7.68 E-04 | -1.12 E-03 | -3.65 E-04 | -5.10 E-04 | -8.09 E-04 |
| $r_{2x}$ | -1.15 E-01 |  |  |  | 2.63 E-01 |  |  | -9.48 E-02 | 6.83 E-01 |  | -3.56 E-01 | 1.12 E-01 | 3.19 E-02 |  |
| $r_{3x}$ | -3.93 E-02 |  |  |  | 8.60 E-01 |  |  | -3.12 E-02 | 5.02 E-01 |  | -2.48 E-01 | 1.11 E-01 | -6.32 E-02 |  |
| $r_{4x}$ | -1.52 E-01 |  |  |  | 4.95 E-01 |  |  | -2.92 E-01 | 5.07 E-01 |  | -1.69 E-01 | 7.68 E-02 | -1.35 E-01 |  |
| $g_{2x}$ |  |  |  |  | 2.34 E-02 |  |  |  |  |  |  |  |  |  |
| $g_{3x}$ |  |  |  |  | 5.01 E-02 |  |  |  |  |  |  |  |  |  |
| $g_{4x}$ |  |  |  |  | 3.71 E-02 |  |  |  |  |  |  |  |  |  |
| $c_{2x}$ |  |  |  |  |  |  |  |  | -6.99 E-04 |  |  |  |  |  |
| $c_{3x}$ |  |  |  |  |  |  |  |  | -5.38 E-04 |  |  |  |  |  |
| $c_{4x}$ |  |  |  |  |  |  |  |  | -5.89 E-04 |  |  |  |  |  |
|  |  |  |  |  |  |  |  |  |  |  |  |  |  |  |
|  |  |  |  |  |  |  |  |  |  |  |  |  |  |  |
| $I_{z}$ | 1.41  E-01 | 6.53 E-01 | -1.48  E-01 | 1.09 E-01 | 4.44 E+00 | 9.76 E-02 | 3.60 E-01 | 4.99 E-02 | 4.43 E-01 | 2.20 E-01 | 1.89 E-01 | -1.77 E-02 | 4.14 E-01 | 1.88 E-02 |
| $B_{z}$ |  | -1.50 E-01 |  | -3.65 E-02 | -1.32 E+00 |  | -7.92 E-02 |  | -1.65 E-01 | -7.56 E-02 |  |  | -9.03 E-02 |  |
| $r_{2z}$ | -5.91 E-02 |  | 2.46  E-01 | 1.01 E-01 |  | -7.46 E-02 | 4.82 E-02 | 4.47 E-02 | 3.11 E-01 | 1.90 E-01 |  | 5.46 E-02 |  | -4.67 E-02 |
| $r_{3z}$ | 4.26 E-02 |  | 3.40  E-01 | 1.01 E-01 |  | -1.85 E-02 | -6.41 E-02 | 1.07 E-01 | 2.15 E-01 | 1.72 E-01 |  | 1.05 E-01 |  | 5.44 E-02 |
| $r_{4z}$ | -1.32 E-01 |  | 2.24  E-01 | 7.99 E-02 |  | -4.55 E-02 | -2.61 E-02 | -4.02 E-01 | 1.37 E-01 | 1.54 E-01 |  | 1.03 E-01 |  | 1.43 E-01 |
| $c_{2z}$ |  |  |  |  |  |  |  |  |  |  |  |  |  |  |
| $c_{3z}$ |  |  |  |  |  |  |  |  |  |  |  |  |  |  |
| $c_{4z}$ |  |  |  |  |  |  |  |  |  |  |  |  |  |  |

|  | *Eschscholzia californica* | *Euphorbia milii* | *Fuchsia* sp. | *Geranium ʹ*Roxanne’ | *Geranium robertianum* | *Geranium sanguineum* | *Lantana* sp. | *Lavandula angustifolia* | *Leucanthemum vulgare* | *Lilium* sp. | *Linum grandiflorum* | *Linum usitatissimum* | *Nepenthes* sp. |
| --- | --- | --- | --- | --- | --- | --- | --- | --- | --- | --- | --- | --- | --- |
|  |  |  |  |  |  |  |  |  |  |  |  |  |  |
| $I_{x}$ | 1.50 E+00 | 1.00 E-01 | 4.50 E-02 | 5.06 E-01 | 4.13 E-01 | 4.59 E-01 | 1.47 E+00 | 4.66 E-01 | 1.78 E+00 | 1.61 E-01 | 2.49 E-01 | 7.66 E-01 | 2.61 E-01 |
| $A_{x}$ | 5.80 E-02 |  |  | -2.02 E-03 |  |  |  |  | 7.86 E-03 | 4.78 E-03 | -2.13 E-03 |  |  |
| $B_{x}$ | -1.35 E-03 | -2.33 E-04 |  | -6.07 E-04 | -5.97 E-04 | -7.67 E-04 | -1.81 E-03 | -7.07 E-04 | -1.81 E-03 |  | -3.24 E-04 | -1.24 E-03 | -3.26 E-04 |
| $r_{2x}$ | 1.05 E+00 | 4.17 E-02 |  | 1.21 E-01 |  | 3.33 E-01 |  | 9.55 E-02 | -3.96 E-01 | -1.49 E-02 | 9.47 E-02 | 3.16 E-02 |  |
| $r_{3x}$ | 8.39 E-01 | 1.90 E-01 |  | 1.64 E-01 |  | 1.42 E-01 |  | 1.11 E-01 | -2.77 E-01 | 6.87 E-02 | 3.47 E-02 | -2.79 E-01 |  |
| $r_{4x}$ | 1.11 E+00 | 1.46 E-01 |  | 2.83 E-02 |  | 2.45 E-02 |  | 2.58 E-01 | -1.67 E-01 | 1.65 E-01 | -1.26 E-01 | -3.75 E-01 |  |
| $g_{2x}$ |  |  |  |  |  |  |  |  |  | -3.31 E-03 | 6.80 E-03 |  |  |
| $g_{3x}$ |  |  |  |  |  |  |  |  |  | -2.96 E-03 | 3.67 E-03 |  |  |
| $g_{4x}$ |  |  |  |  |  |  |  |  |  | -1.54 E-02 | 2.24 E-03 |  |  |
| $c_{2x}$ |  |  |  |  |  | -2.71 E-04 |  |  |  |  | 7.87 E-07 | 2.50 E-04 |  |
| $c_{3x}$ |  |  |  |  |  | 1.39 E-04 |  |  |  |  | 4.30 E-05 | 6.20 E-04 |  |
| $c_{4x}$ |  |  |  |  |  | 2.52 E-04 |  |  |  |  | 7.31 E-05 | 7.27 E-04 |  |
|  |  |  |  |  |  |  |  |  |  |  |  |  |  |
|  |  |  |  |  |  |  |  |  |  |  |  |  |  |
| $I_{z}$ | 1.54 E+00 | -8.29 E-02 | 3.39 E-02 | 2.86 E-01 | 7.61 E-03 | 1.33 E-01 | 6.20 E-01 | 4.79 E-02 | 8.95 E-01 | 1.85 E-01 | 1.12 E-01 | -3.48 E-02 | 6.59 E-02 |
| $B_{z}$ | -4.64 E-01 |  |  | -9.52 E-02 |  | -6.01 E-02 | -1.46 E-01 |  | -2.34 E-01 |  | -4.63 E-02 |  |  |
| $r_{2z}$ | 4.23 E+00 | 1.27 E-01 | -4.60 E-02 | 8.16 E-02 | 1.02 E-01 | 5.94 E-02 |  | 3.50 E-02 | -3.54 E-01 | -9.52 E-02 | 1.75 E-01 | 8.78 E-02 | 3.31 E-02 |
| $r_{3z}$ | 4.65 E+00 | 2.33 E-01 | 6.45 E-02 | 1.25 E-01 | 6.26 E-02 | 2.05 E-01 |  | 1.63 E-01 | -4.71 E-01 | 1.99 E-02 | 9.12 E-02 | 1.44 E-01 | -1.08 E-02 |
| $r_{4z}$ | 7.85 E+00 | 8.87 E-02 | 1.85 E-01 | 3.85 E-02 | 9.53 E-02 | 2.04 E-01 |  | 2.33 E-01 | -3.01 E-01 | -8.76 E-02 | 3.90 E-03 | 1.51 E-01 | -1.65 E-02 |
| $c_{2z}$ | -1.19 E+00 |  |  |  |  |  |  |  | 2.54 E-02 |  |  |  |  |
| $c_{3z}$ | -1.32 E+00 |  |  |  |  |  |  |  | 1.97 E-01 |  |  |  |  |
| $c_{4z}$ | -2.29 E+00 |  |  |  |  |  |  |  | 1.53 E-01 |  |  |  |  |

|  | *Nicotiana tabacum* | *Oenothera caespitosa* | *Osteospermum* sp. | *Papaver cambricum* | *Papaver rhoeas* | *Potentilla* sp. | *Ranunculus acris* | *Ranunculus lingua* | *Rudbeckia hirta* | *Scabiosa* sp. | *Taraxacum* agg*.* | *Trifolium pratense* | *Tulbaghia violacea* |
| --- | --- | --- | --- | --- | --- | --- | --- | --- | --- | --- | --- | --- | --- |
|  |  |  |  |  |  |  |  |  |  |  |  |  |  |
| $I_{x}$ | 1.02 E-01 | 1.78 E+00 | 6.24 E-01 | 5.79 E-01 | 2.80 E-01 | 6.94 E-01 | 1.53 E+00 | 3.09 E+00 | 1.08 E+00 | 1.36 E+00 | 1.49 E+00 | 6.11 E-01 | 5.24 E-01 |
| $A_{x}$ |  | 9.17 E-03 | 1.11 E-03 |  | -2.62 E-03 | 1.98 E-03 |  | 1.76 E-02 | 6.50 E-03 | 4.90 E-03 |  |  |  |
| $B_{x}$ | -9.82 E-05 | -1.81 E-03 | -6.50 E-04 | -6.15 E-04 | -3.01 E-04 | -8.05 E-04 | -1.88 E-03 | -3.37 E-03 | -1.09 E-03 | -1.52 E-03 | -1.92 E-03 | -6.32 E-04 | -6.04 E-04 |
| $r_{2x}$ |  | -3.36 E-01 | 5.49 E-01 |  |  | -2.60 E-02 | 1.88 E+00 | 4.86 E-02 | 1.61 E-01 |  | 3.90 E-01 | 1.48 E-02 | -3.39 E-01 |
| $r_{3x}$ |  | -5.21 E-01 | 5.41 E-01 |  |  | -9.94 E-02 | 1.62 E+00 | -3.26 E-01 | -1.40 E-01 |  | 1.27 E+00 | -2.33 E-01 | -1.06 E-01 |
| $r_{4x}$ |  | -8.45 E-01 | 1.31 E-01 |  |  | -6.37 E-02 | 3.11 E-01 | -3.31 E-01 | -2.27 E-01 |  | 1.86 E+00 | -2.59 E-01 | -1.66 E-01 |
| $g_{2x}$ |  | -7.91 E-04 | 1.01 E-02 |  |  |  |  |  |  |  |  |  |  |
| $g_{3x}$ |  | 4.87 E-04 | 1.20 E-02 |  |  |  |  |  |  |  |  |  |  |
| $g_{4x}$ |  | 6.68 E-04 | 3.31 E-03 |  |  |  |  |  |  |  |  |  |  |
| $c_{2x}$ |  | 4.47 E-04 | -5.33 E-04 |  |  |  | -2.71 E-03 |  |  |  | -4.51 E-04 | -1.38 E-04 | 3.40 E-04 |
| $c_{3x}$ |  | 6.31 E-04 | -5.45 E-04 |  |  |  | -2.33 E-03 |  |  |  | -1.66 E-03 | 1.07 E-04 | 1.93 E-04 |
| $c_{4x}$ |  | 1.09 E-03 | -2.16 E-04 |  |  |  | -5.41 E-04 |  |  |  | -2.24 E-03 | 1.42 E-04 | 3.57 E-04 |
|  |  |  |  |  |  |  |  |  |  |  |  |  |  |
|  |  |  |  |  |  |  |  |  |  |  |  |  |  |
| $I_{z}$ | 5.25 E-02 | 1.14 E+00 | 4.40 E-01 | 3.33 E-01 | 2.31 E-01 | 2.30 E-01 | 1.41 E+00 | 1.58 E+00 | 5.69 E-01 | 7.65 E-01 | 5.97 E-01 | 1.91 E-01 | 2.33 E-01 |
| $B_{z}$ |  | -2.53 E-01 | -1.21 E-01 | -6.91 E-02 |  | -4.34 E-02 | -4.44 E-01 | -4.08 E-01 | -1.34 E-01 | -2.01 E-01 | -1.64 E-01 |  |  |
| $r_{2z}$ | -1.55 E-02 |  | 1.51 E+00 |  | -1.45 E-01 |  | 1.97 E-01 | -4.29 E-02 |  |  | 2.16 E-01 | -7.95 E-02 | -1.62 E-01 |
| $r_{3z}$ | 1.29 E-02 |  | 1.43 E+00 |  | -1.29 E-01 |  | 2.37 E-01 | -5.73 E-02 |  |  | 7.37 E-01 | -1.27 E-01 | -8.42 E-02 |
| $r_{4z}$ | -1.38 E-02 |  | 3.07 E-01 |  | -1.22 E-01 |  | 7.83 E-02 | -2.49 E-02 |  |  | 2.62 E+00 | -1.69 E-01 | -2.78 E-02 |
| $c_{2z}$ |  |  | -4.44 E-01 |  |  |  |  |  |  |  | -5.39 E-02 |  |  |
| $c_{3z}$ |  |  | -4.20 E-01 |  |  |  |  |  |  |  | -2.11 E-01 |  |  |
| $c_{4z}$ |  |  | -9.16 E-02 |  |  |  |  |  |  |  | -7.91 E-01 |  |  |

|  | *Vinca herbacea* | *Xerochrysum bracteatum* |  | Control TLP | Control TL | Control T | Control TWLP | Control TWL | Control TW |
| --- | --- | --- | --- | --- | --- | --- | --- | --- | --- |
|  |  |  |  |  |  |  |  |  |  |
| $I_{x}$ | 2.28 E-01 | 3.66 E+00 |  | 1.37 E-01 | 7.96 E-02 | 2.72 E-02 | 3.82 E-02 | 4.57 E-01 | 1.17  E+00 |
| $A_{x}$ | -1.07 E-03 | 1.27 E-02 |  |  | -5.68 E-04 | -4.42 E-04 | -8.09 E-04 |  |  |
| $B_{x}$ | -2.15 E-04 | -4.51 E-03 |  |  |  | -4.41 E-05 |  | -5.98 E-04 | -1.53  E-03 |
| $r_{2x}$ | 1.11 E-02 |  |  | -1.81 E-01 | -7.16 E-02 | -4.55 E-04 | -5.18 E-02 |  |  |
| $r_{3x}$ | -2.95 E-02 |  |  | -8.72 E-02 | -4.39 E-02 | 3.14 E-02 | 2.30 E-02 |  |  |
| $r_{4x}$ | -3.20 E-02 |  |  | -1.56 E-01 | -7.59 E-02 | 1.38 E-02 | -8.80 E-02 |  |  |
| $g_{2x}$ |  |  |  |  |  |  |  |  |  |
| $g_{3x}$ |  |  |  |  |  |  |  |  |  |
| $g_{4x}$ |  |  |  |  |  |  |  |  |  |
| $c_{2x}$ |  |  |  |  |  |  |  |  |  |
| $c_{3x}$ |  |  |  |  |  |  |  |  |  |
| $c_{4x}$ |  |  |  |  |  |  |  |  |  |
|  |  |  |  |  |  |  |  |  |  |
|  |  |  |  |  |  |  |  |  |  |
| $I_{z}$ | 6.28 E-02 | 1.46 E+00 |  | 9.38 E-02 | 7.14 E-02 | -1.30 E-02 | 9.06 E-02 | 6.05 E-02 | 0.53  E+00 |
| $B_{z}$ |  | -3.74 E-01 |  |  |  | 1.73 E-02 |  |  | -0.13  E+00 |
| $r_{2z}$ |  |  |  | -1.46 E-01 | -4.51 E-02 |  | -4.64 E-02 |  |  |
| $r_{3z}$ |  |  |  | -3.59 E-02 | -4.26 E-03 |  | -3.98 E-02 |  |  |
| $r_{4z}$ |  |  |  | -8.78 E-02 | -4.10 E-02 |  | -1.31 E-01 |  |  |
| $c_{2z}$ |  |  |  |  |  |  |  |  |  |
| $c_{3z}$ |  |  |  |  |  |  |  |  |  |
| $c_{4z}$ |  |  |  |  |  |  |  |  |  |

**Fig. S1:** A scale floorplan of the lab where humidity signal sampling took place, adapted from building and arm installation blueprints. The location of elements related to humidity signal sampling are indicated. The ‘door’ and ‘robot’ sides of the room as well as the robot’s coordination of *x* and *y* axis are indicated, image is looking down the *z* axis.


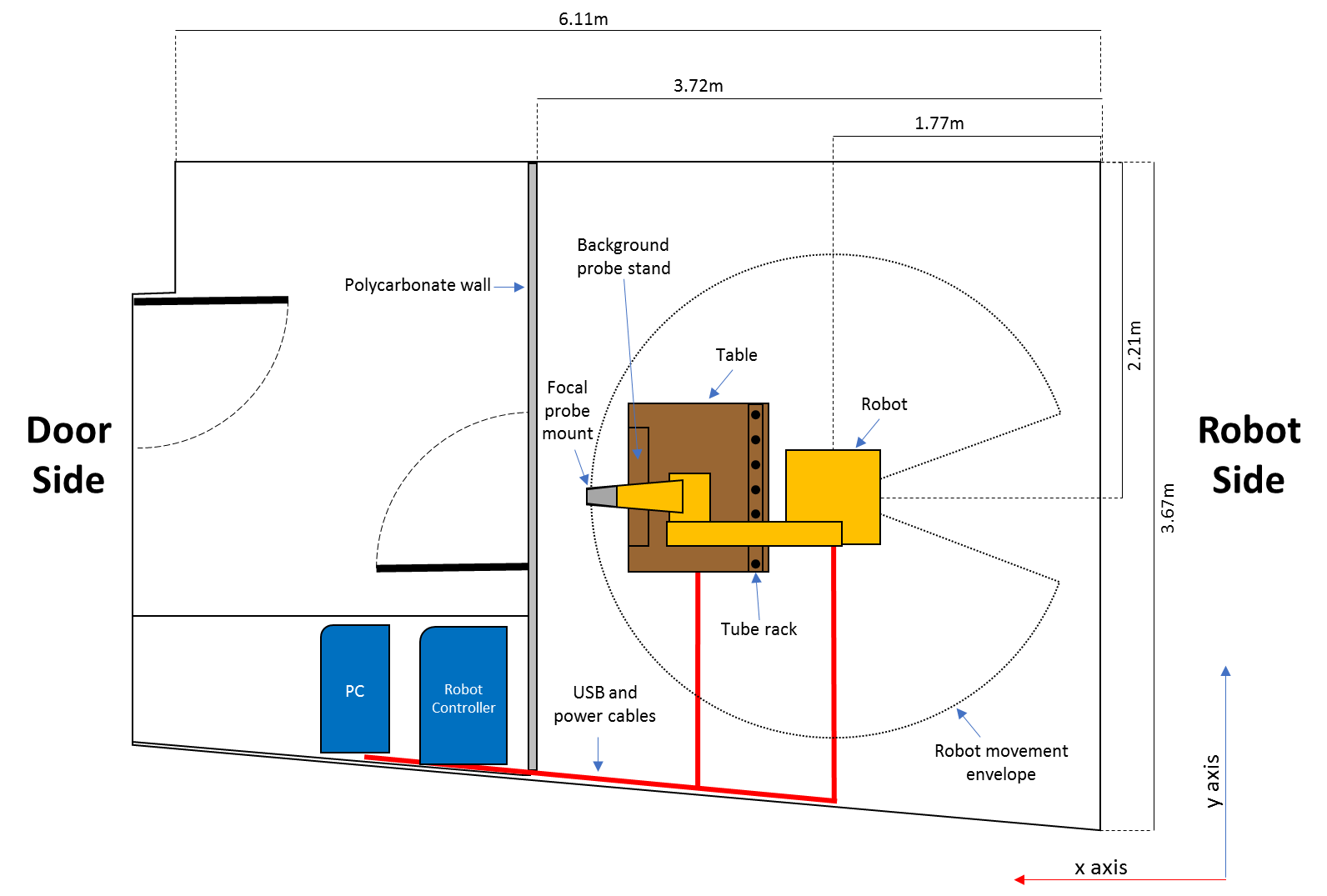

Supplement: SUPPLEMENTARY FILE 1 — Includes the following appendices. (Appendix S1) AIC tables and sampling dates of flower species floral humidity analyses. (Appendix S2) AIC tables and sampling dates of control tube humidity analyses. (Appendix S3) Derivation of Eq. (12). (Appendix S4) The parameter values of the best-fitting humidity structure models. Also includes, (Figure S1) A scale floorplan of the lab where humidity signal sampling took place. [file Data_Sheet_1.docx]
