# Supplementary material for: Floral Humidity in Flowering Plants: A Preliminary Survey
Source: Front Plant Sci. 2020 Mar 6;11:249. doi: 10.3389/fpls.2020.00249 (PMC7068853; doi:10.3389/fpls.2020.00249)
Supplement: SUPPLEMENTARY FILE 2 — A zipped file containing floral humidity data collected by the robot arm transects as described in the main text. A word document within the zipped files explains the data formatting in detail. Datafiles themselves are in CSV format. [file Data_Sheet_2.zip › Floral humidity data/Floral Humidity Data guide.docx]

**Floral humidity data guide**

Attached is the floral humidity data collected by the robot arm transects as described in the main text. For each flower species and control two csv files are provided. The ‘Raw’ datafile, indicated by ‘[*species/control name*]Raw’ contains all individual humidity measurements taken throughout sampling of all transects pertaining to each species or control (i.e. all the individual measurements made in each measurement period). The ‘Means’ datafile, indicated by ‘[*species/control name*]Means’ contains the mean averages of each measurement period pertaining to each species or control. These mean values are used in assessments of floral humidity as described in the main text and are the data plotted in the floral humidity graphs.

**Guide to Column headings: ‘Raw’ datafile**

| [unlabeled] | - | A counter of humidity measurement datapoints within the file. |
| --- | --- | --- |
| X. | - | Humidity measurement number, aids robot arm operation. This number is applied by the humidity probes and counts up with each measurement. This value is reset periodically. |
| Species | - | The species or control being measured. |
| Individual | - | The individual flower identification number within each few sampling days – This value resets across each few days of sampling, but is used to generate the totIndividual values that identify individual flowers across all humidity sampling days. |
| totIndividual | - | The individual flower identification number across all humidity sampling - $n$ within model equations. |
| date | - | The time and date of the measurement, formatted as *yyyy-mm-dd-hh-mm-ss.* |
| sample.. | - | The replicate transect number of the measurement point. 0 = first transect, 1 = second transect, 2 = third transect, 3 = forth transect. |
| Sample.order | - | The number of the flower individual within the randomly selected sequence in which flowers were sampled. 0 = first flower in sampling order, 1 = second flower in sampling order, 2 = third flower in sampling order, 3 = forth flower in sampling order. |
| x.or.z.transect | - | The indicator of the transect being carried out– x or z |
| xoffset | - | The x axis offset of the current measurement. |
| zoffset | - | The z axis offset of the current measurement. |
| Background.Humidity | - | The background humidity measurement. |
| Background.Temperature | - | The background temperature measurement - The temperature measurement taken by the background probe. |
| Focal.Humidity | - | The uncorrected focal humidity measurement |
| Focal.Temperature | - | The temperature measurement taken by the focal probe. |
| Corrected.Focal.humidity | - | The corrected focal humidity measurement. |
| Change.in.RH. | - | The change in humidity between focal and background probes ($\Delta RH$) |
| block | - | The current measurement period number – this value counts up across all sampling. |
| BE | - | An indicator of whether the measurements where taken within the first or last 10 seconds of the measurement period. ‘First10’ indicates the measurement was taken in the first 10 seconds of the measurement period. ‘Last10’ indicates the measurement was taken within the last 10 seconds of the measurement period. |
| Tag | - | An indicator of measurements that take place within the first or last 10 seconds of the measurement period. Relevant points are indicated by ‘Here’ |
| N | - | The number of the measurement within each measurement period. |

**Guide to Column headings: ‘Means’ datafile**

| [unlabeled] | - | A counter of datapoints within the file. |
| --- | --- | --- |
| Block | - | The measurement period |
| Sp. | - | The species or control being measured. |
| xORz | - | Indicator as to transect being carried out – x or z |
| Individual | - | The individual flower identification number within each few sampling days – This value resets across each few days of sampling, but is used to generate the totIndividual values that identify individual flowers across all humidity sampling days. |
| totIndividual | - | The individual flower identification number across all humidity sampling - $n$ within model equations. |
| sample.. | - | The replicate transect number of the measurement point. 0 = first transect, 1 = second transect, 2 = third transect, 3 = forth transect. |
| Sample.order | - | The number of the flower individual within the randomly selected sequence in which flowers were sampled. 0 = first flower in sampling order, 1 = second flower in sampling order, 2 = third flower in sampling order, 3 = forth flower in sampling order. |
| xoffset | - | The x axis offset of the current measurement period. |
| zoffset | - | The z axis offset of the current measurement period. |
| x2 | - | The x axis offset squared (used in analyses). |
| z2 | - | The z axis offset squared (ultimately not used in any analyses). |
| Background.Humidity | - | The mean background humidity measurement of the measurement period. |
| Background.Temperature | - | The mean background temperature measurement of the measurement period. |
| Focal.Humidity | - | The mean uncorrected focal humidity measurement of the measurement period. |
| Focal.Temperature | - | The mean temperature measurement taken by the focal probe of the measurement period. |
| Change.in.RH. | - | The mean change in humidity between focal and background probes ($\Delta RH$) of the measurement period. |
