# Supplementary material for: Floral Humidity in Flowering Plants: A Preliminary Survey
Source: Front Plant Sci. 2020 Mar 6;11:249. doi: 10.3389/fpls.2020.00249 (PMC7068853; doi:10.3389/fpls.2020.00249)
Supplement: SUPPLEMENTARY FILE 3 — A zipped file containing floral humidity structures for the flower species and controls sampled within the study as described in the main text. A word document within the zipped files explains the data set in detail. [file Data_Sheet_3.zip › Floral humidity graphs/Floral humidity graph key.docx]

**Floral Humidity graphs key**

Contained within the attached files are graphs presenting floral humidity structures for the flower species and controls sampled within the study as described in the main text. Flowers and controls are separated in two files. For each species, and each control, two graphs are provided. These graphs each present the difference in humidity relative to the background ($\Delta RH)$ across the x and z axis transects. All axis offsets are relative to the transect central point and in millimetres. The thin dotted line indicates a 0% change in humidity (the background level). Bold lines indicate the mean change in humidity as predicted by the best fitting model for that flower or control. Colour and dashing of bold lines and points indicate the replicate transect: solid black, first transect; long-dash blue, second transect; dash-dot orange, third transect; dotted green, forth transect. In the x axis transect graphs the solid bar indicates the mean flower span for that species relative to the x axis. In control x axis graphs this solid bar indicates the 23mm span of the horticultural tube.
