# Supplementary figures and images for: Floral Humidity in Flowering Plants: A Preliminary Survey
Source: Front Plant Sci. 2020 Mar 6;11:249. doi: 10.3389/fpls.2020.00249 (PMC7068853; doi:10.3389/fpls.2020.00249)

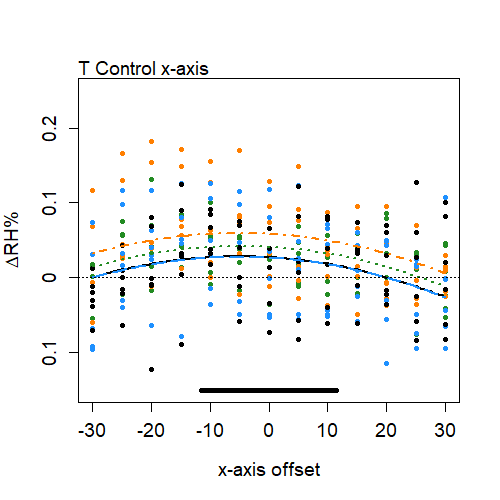

Supplement: SUPPLEMENTARY FILE 3 — A zipped file containing floral humidity structures for the flower species and controls sampled within the study as described in the main text. A word document within the zipped files explains the data set in detail. [file Data_Sheet_3.zip › Floral humidity graphs/Controls/T X axis.png]

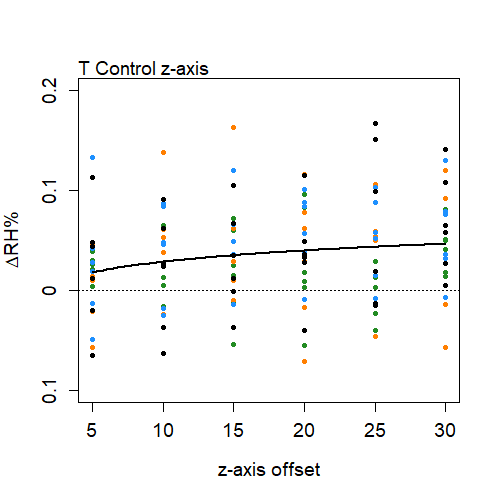

Supplement: SUPPLEMENTARY FILE 3 — A zipped file containing floral humidity structures for the flower species and controls sampled within the study as described in the main text. A word document within the zipped files explains the data set in detail. [file Data_Sheet_3.zip › Floral humidity graphs/Controls/T Z axis.png]

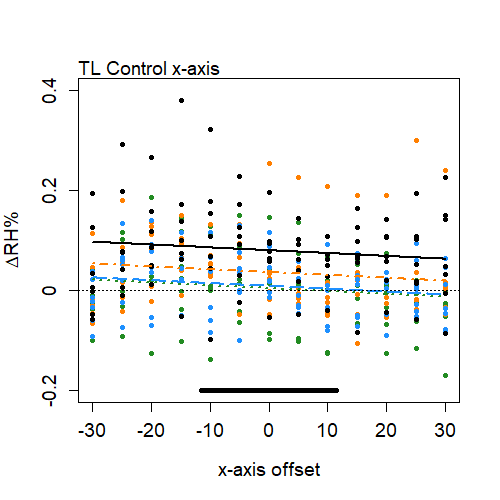

Supplement: SUPPLEMENTARY FILE 3 — A zipped file containing floral humidity structures for the flower species and controls sampled within the study as described in the main text. A word document within the zipped files explains the data set in detail. [file Data_Sheet_3.zip › Floral humidity graphs/Controls/TL X axis.png]

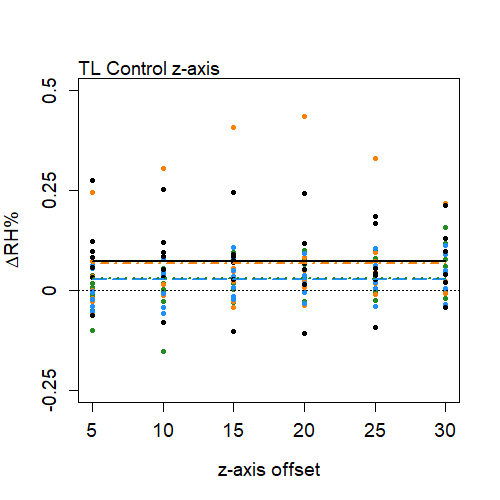

Supplement: SUPPLEMENTARY FILE 3 — A zipped file containing floral humidity structures for the flower species and controls sampled within the study as described in the main text. A word document within the zipped files explains the data set in detail. [file Data_Sheet_3.zip › Floral humidity graphs/Controls/TL Z axis.png]

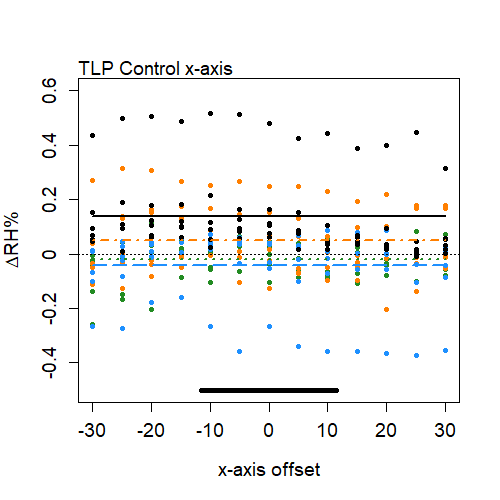

Supplement: SUPPLEMENTARY FILE 3 — A zipped file containing floral humidity structures for the flower species and controls sampled within the study as described in the main text. A word document within the zipped files explains the data set in detail. [file Data_Sheet_3.zip › Floral humidity graphs/Controls/TLP X axis.png]

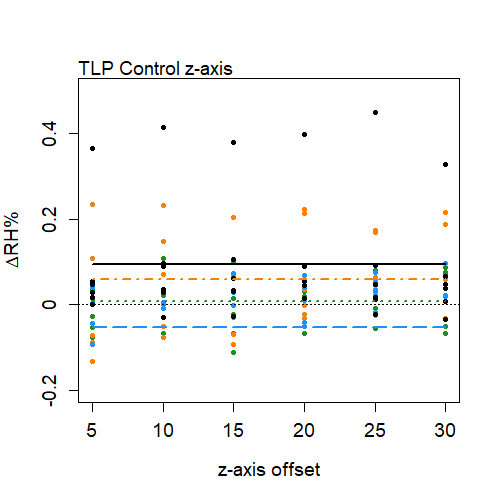

Supplement: SUPPLEMENTARY FILE 3 — A zipped file containing floral humidity structures for the flower species and controls sampled within the study as described in the main text. A word document within the zipped files explains the data set in detail. [file Data_Sheet_3.zip › Floral humidity graphs/Controls/TLP Z axis.png]

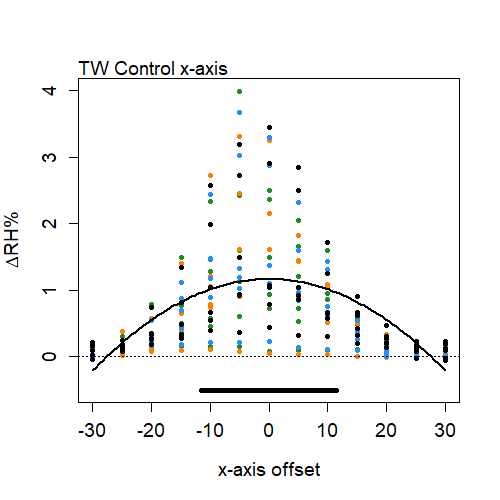

Supplement: SUPPLEMENTARY FILE 3 — A zipped file containing floral humidity structures for the flower species and controls sampled within the study as described in the main text. A word document within the zipped files explains the data set in detail. [file Data_Sheet_3.zip › Floral humidity graphs/Controls/TW X axis.png]

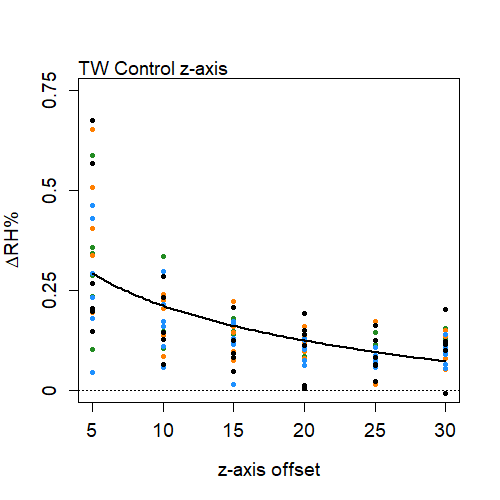

Supplement: SUPPLEMENTARY FILE 3 — A zipped file containing floral humidity structures for the flower species and controls sampled within the study as described in the main text. A word document within the zipped files explains the data set in detail. [file Data_Sheet_3.zip › Floral humidity graphs/Controls/TW Z axis.png]

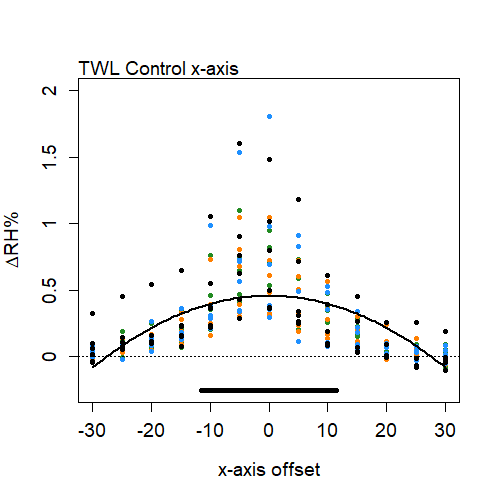

Supplement: SUPPLEMENTARY FILE 3 — A zipped file containing floral humidity structures for the flower species and controls sampled within the study as described in the main text. A word document within the zipped files explains the data set in detail. [file Data_Sheet_3.zip › Floral humidity graphs/Controls/TWL X axis.png]

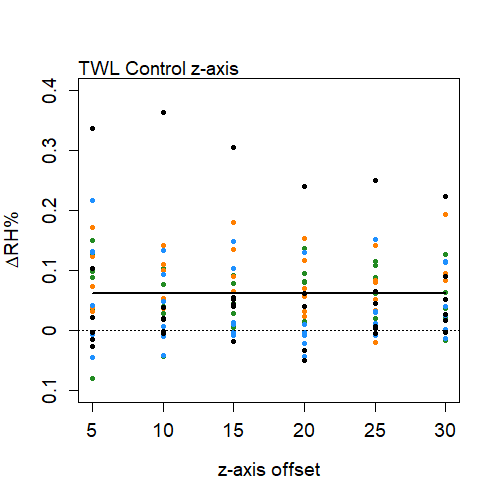

Supplement: SUPPLEMENTARY FILE 3 — A zipped file containing floral humidity structures for the flower species and controls sampled within the study as described in the main text. A word document within the zipped files explains the data set in detail. [file Data_Sheet_3.zip › Floral humidity graphs/Controls/TWL Z axis.png]

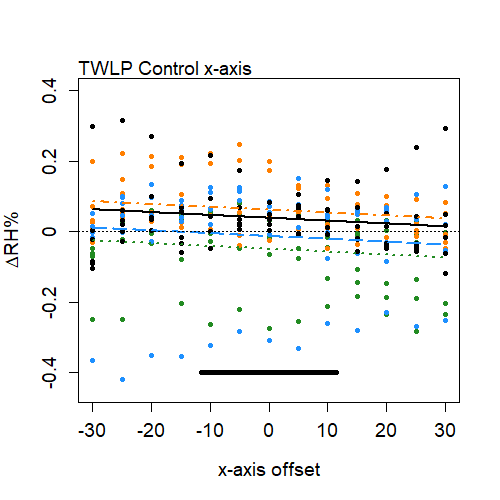

Supplement: SUPPLEMENTARY FILE 3 — A zipped file containing floral humidity structures for the flower species and controls sampled within the study as described in the main text. A word document within the zipped files explains the data set in detail. [file Data_Sheet_3.zip › Floral humidity graphs/Controls/TWLP X axis.png]

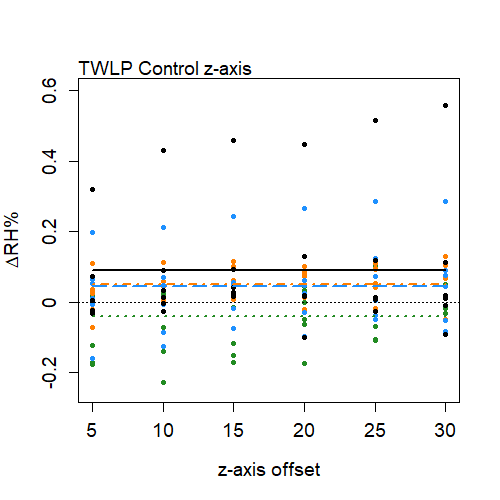

Supplement: SUPPLEMENTARY FILE 3 — A zipped file containing floral humidity structures for the flower species and controls sampled within the study as described in the main text. A word document within the zipped files explains the data set in detail. [file Data_Sheet_3.zip › Floral humidity graphs/Controls/TWLP Z axis.png]

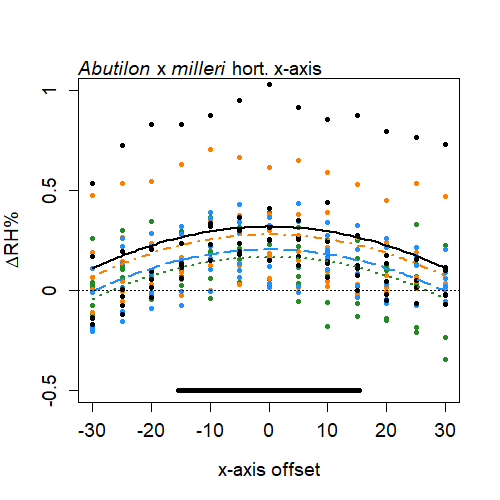

Supplement: SUPPLEMENTARY FILE 3 — A zipped file containing floral humidity structures for the flower species and controls sampled within the study as described in the main text. A word document within the zipped files explains the data set in detail. [file Data_Sheet_3.zip › Floral humidity graphs/Flowers/Abutilon x milleri hort. X axis.png]

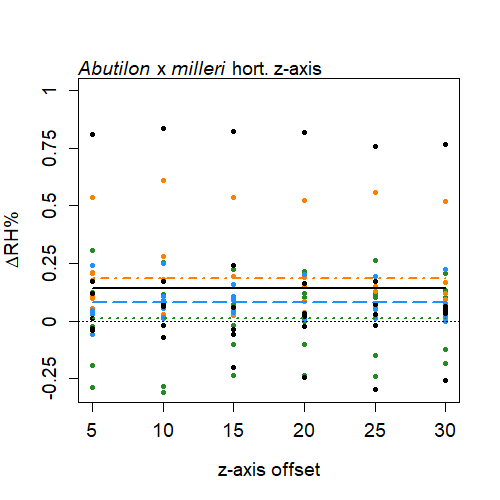

Supplement: SUPPLEMENTARY FILE 3 — A zipped file containing floral humidity structures for the flower species and controls sampled within the study as described in the main text. A word document within the zipped files explains the data set in detail. [file Data_Sheet_3.zip › Floral humidity graphs/Flowers/Abutilon x milleri hort. Z axis.png]

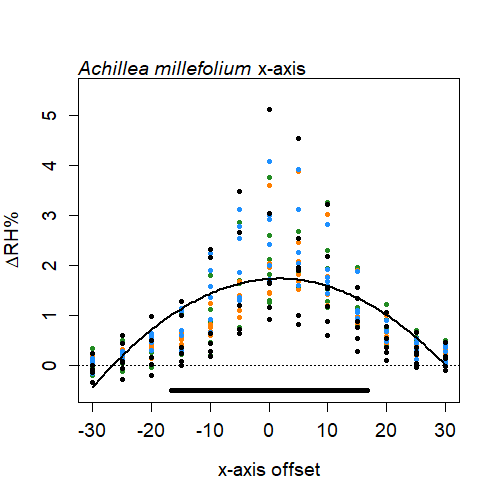

Supplement: SUPPLEMENTARY FILE 3 — A zipped file containing floral humidity structures for the flower species and controls sampled within the study as described in the main text. A word document within the zipped files explains the data set in detail. [file Data_Sheet_3.zip › Floral humidity graphs/Flowers/Achillea millefolium X axis.png]

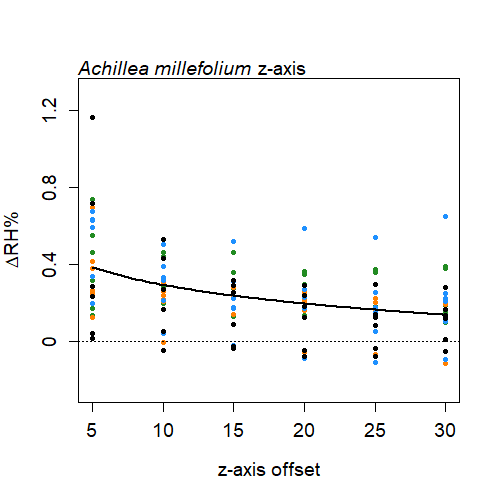

Supplement: SUPPLEMENTARY FILE 3 — A zipped file containing floral humidity structures for the flower species and controls sampled within the study as described in the main text. A word document within the zipped files explains the data set in detail. [file Data_Sheet_3.zip › Floral humidity graphs/Flowers/Achillea millefolium Z axis.png]

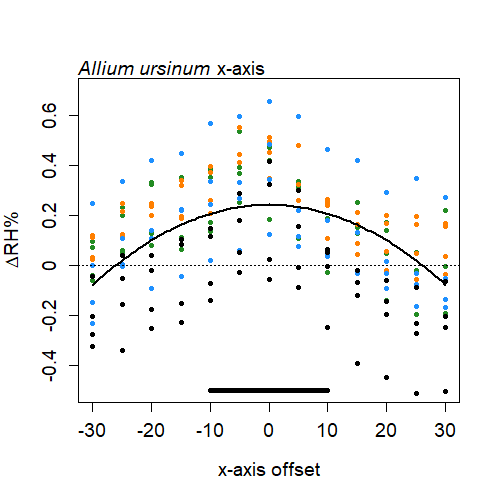

Supplement: SUPPLEMENTARY FILE 3 — A zipped file containing floral humidity structures for the flower species and controls sampled within the study as described in the main text. A word document within the zipped files explains the data set in detail. [file Data_Sheet_3.zip › Floral humidity graphs/Flowers/Allium ursinum X axis.png]

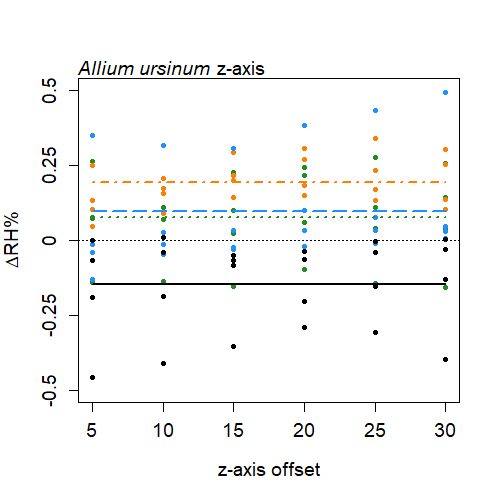

Supplement: SUPPLEMENTARY FILE 3 — A zipped file containing floral humidity structures for the flower species and controls sampled within the study as described in the main text. A word document within the zipped files explains the data set in detail. [file Data_Sheet_3.zip › Floral humidity graphs/Flowers/Allium ursinum Z axis.png]

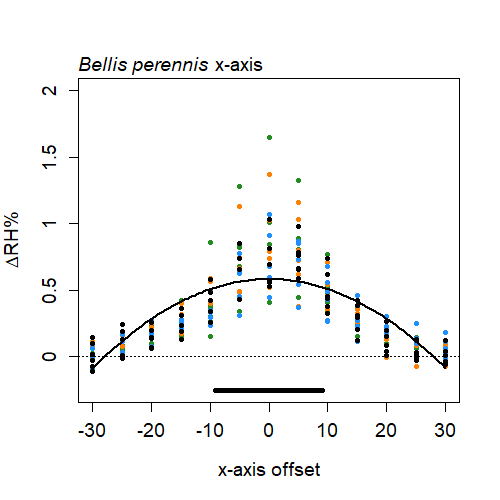

Supplement: SUPPLEMENTARY FILE 3 — A zipped file containing floral humidity structures for the flower species and controls sampled within the study as described in the main text. A word document within the zipped files explains the data set in detail. [file Data_Sheet_3.zip › Floral humidity graphs/Flowers/Bellis perennis X axis.png]

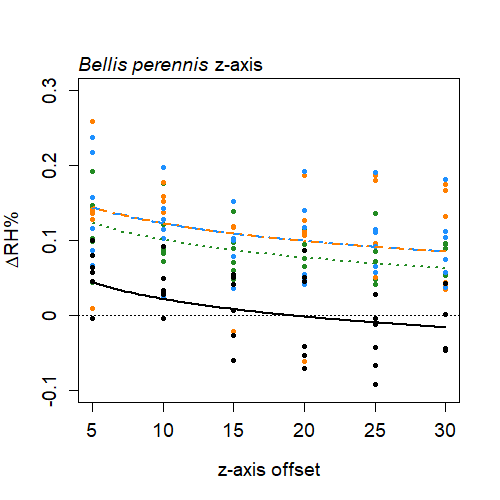

Supplement: SUPPLEMENTARY FILE 3 — A zipped file containing floral humidity structures for the flower species and controls sampled within the study as described in the main text. A word document within the zipped files explains the data set in detail. [file Data_Sheet_3.zip › Floral humidity graphs/Flowers/Bellis perennis Z axis.png]

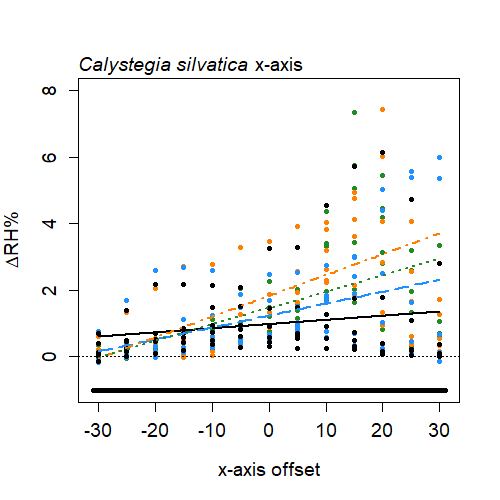

Supplement: SUPPLEMENTARY FILE 3 — A zipped file containing floral humidity structures for the flower species and controls sampled within the study as described in the main text. A word document within the zipped files explains the data set in detail. [file Data_Sheet_3.zip › Floral humidity graphs/Flowers/Calystegia silvatica X axis.png]

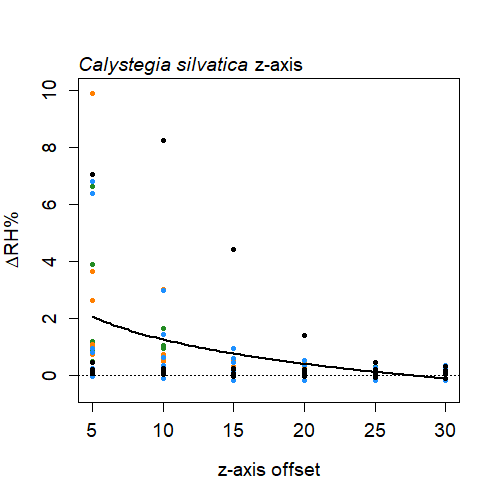

Supplement: SUPPLEMENTARY FILE 3 — A zipped file containing floral humidity structures for the flower species and controls sampled within the study as described in the main text. A word document within the zipped files explains the data set in detail. [file Data_Sheet_3.zip › Floral humidity graphs/Flowers/Calystegia silvatica Z axis.png]

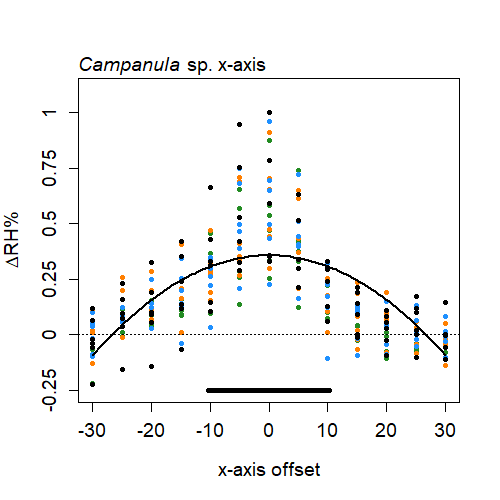

Supplement: SUPPLEMENTARY FILE 3 — A zipped file containing floral humidity structures for the flower species and controls sampled within the study as described in the main text. A word document within the zipped files explains the data set in detail. [file Data_Sheet_3.zip › Floral humidity graphs/Flowers/Campanula X axis.png]

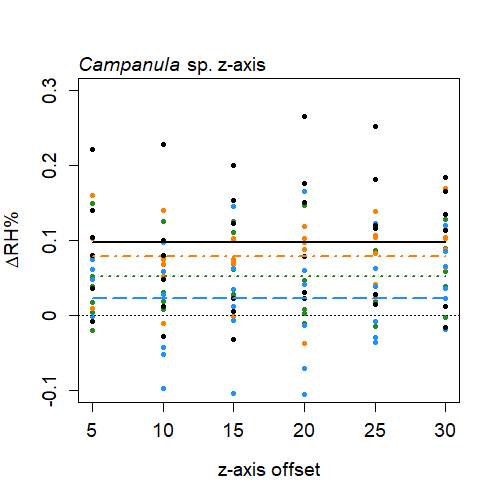

Supplement: SUPPLEMENTARY FILE 3 — A zipped file containing floral humidity structures for the flower species and controls sampled within the study as described in the main text. A word document within the zipped files explains the data set in detail. [file Data_Sheet_3.zip › Floral humidity graphs/Flowers/Campanula Z axis.png]

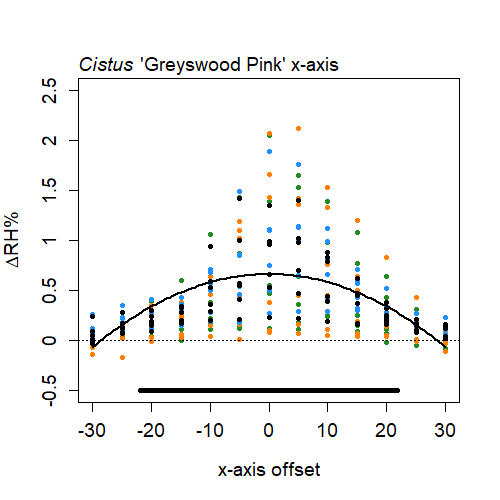

Supplement: SUPPLEMENTARY FILE 3 — A zipped file containing floral humidity structures for the flower species and controls sampled within the study as described in the main text. A word document within the zipped files explains the data set in detail. [file Data_Sheet_3.zip › Floral humidity graphs/Flowers/Cistus 'Greyswood Pink' X axis.png]

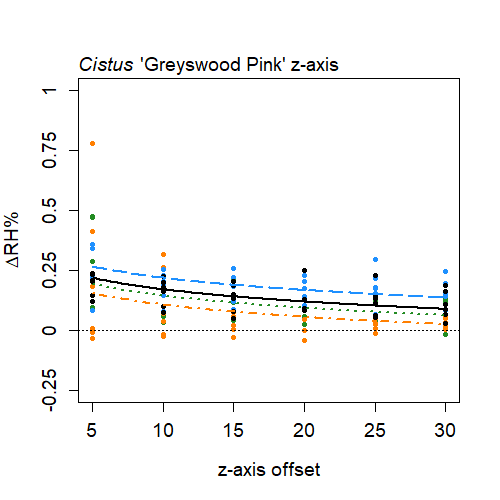

Supplement: SUPPLEMENTARY FILE 3 — A zipped file containing floral humidity structures for the flower species and controls sampled within the study as described in the main text. A word document within the zipped files explains the data set in detail. [file Data_Sheet_3.zip › Floral humidity graphs/Flowers/Cistus 'Greyswood Pink' Z axis.png]

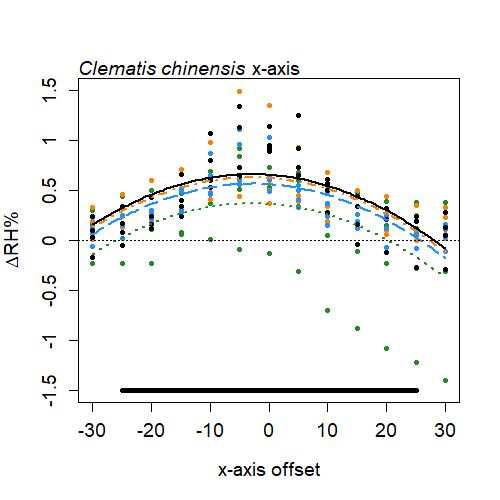

Supplement: SUPPLEMENTARY FILE 3 — A zipped file containing floral humidity structures for the flower species and controls sampled within the study as described in the main text. A word document within the zipped files explains the data set in detail. [file Data_Sheet_3.zip › Floral humidity graphs/Flowers/Clematis chinensis X axis.png]

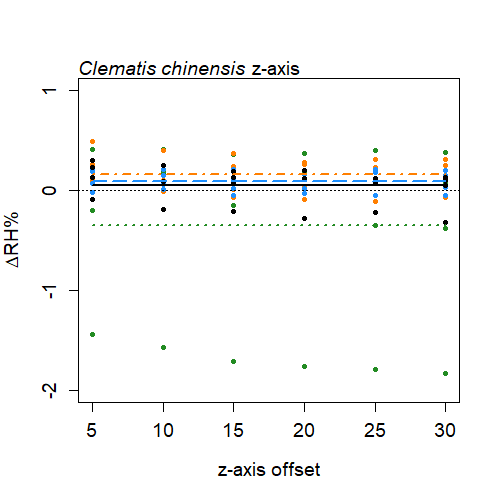

Supplement: SUPPLEMENTARY FILE 3 — A zipped file containing floral humidity structures for the flower species and controls sampled within the study as described in the main text. A word document within the zipped files explains the data set in detail. [file Data_Sheet_3.zip › Floral humidity graphs/Flowers/Clematis chinensis Z axis.png]

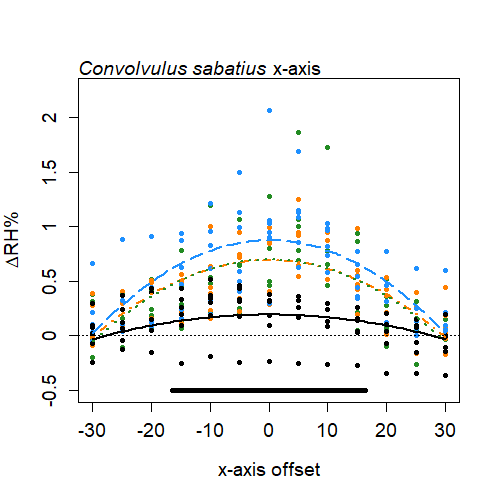

Supplement: SUPPLEMENTARY FILE 3 — A zipped file containing floral humidity structures for the flower species and controls sampled within the study as described in the main text. A word document within the zipped files explains the data set in detail. [file Data_Sheet_3.zip › Floral humidity graphs/Flowers/Convolvulus sabatius X axis.png]

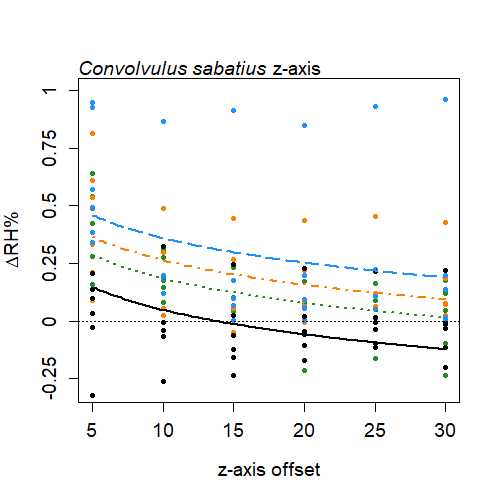

Supplement: SUPPLEMENTARY FILE 3 — A zipped file containing floral humidity structures for the flower species and controls sampled within the study as described in the main text. A word document within the zipped files explains the data set in detail. [file Data_Sheet_3.zip › Floral humidity graphs/Flowers/Convolvulus sabatius Z axis.png]

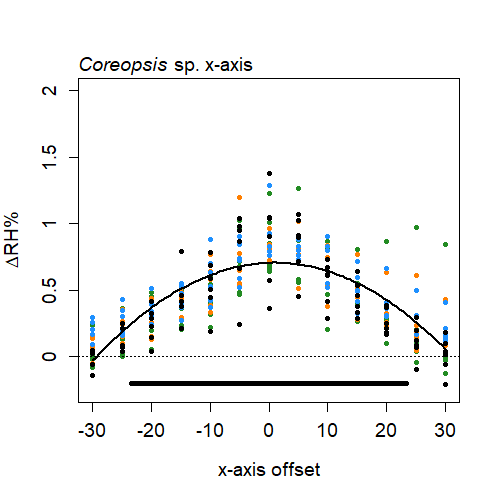

Supplement: SUPPLEMENTARY FILE 3 — A zipped file containing floral humidity structures for the flower species and controls sampled within the study as described in the main text. A word document within the zipped files explains the data set in detail. [file Data_Sheet_3.zip › Floral humidity graphs/Flowers/Coreopsis sp X axis.png]

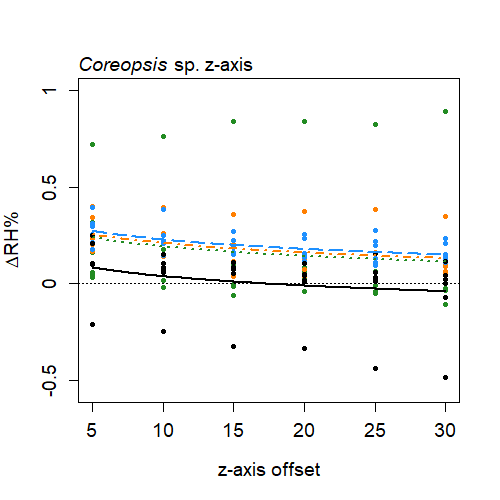

Supplement: SUPPLEMENTARY FILE 3 — A zipped file containing floral humidity structures for the flower species and controls sampled within the study as described in the main text. A word document within the zipped files explains the data set in detail. [file Data_Sheet_3.zip › Floral humidity graphs/Flowers/Coreopsis Z axis.png]

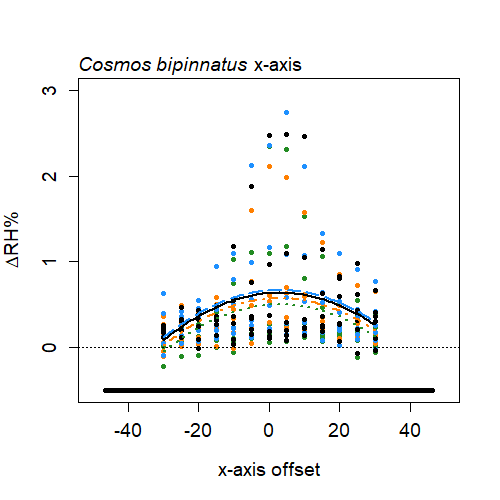

Supplement: SUPPLEMENTARY FILE 3 — A zipped file containing floral humidity structures for the flower species and controls sampled within the study as described in the main text. A word document within the zipped files explains the data set in detail. [file Data_Sheet_3.zip › Floral humidity graphs/Flowers/Cosmos bipinnatus X axis.png]

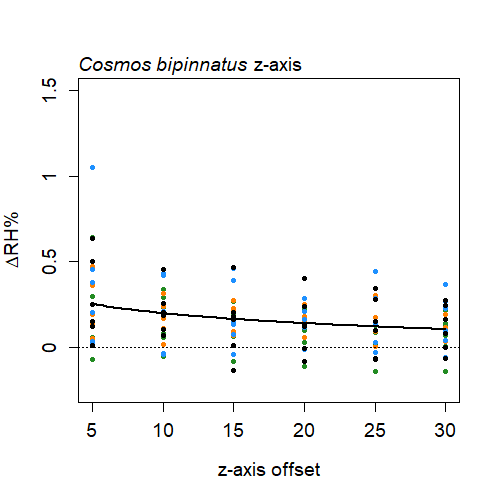

Supplement: SUPPLEMENTARY FILE 3 — A zipped file containing floral humidity structures for the flower species and controls sampled within the study as described in the main text. A word document within the zipped files explains the data set in detail. [file Data_Sheet_3.zip › Floral humidity graphs/Flowers/Cosmos bipinnatus Z axis.png]

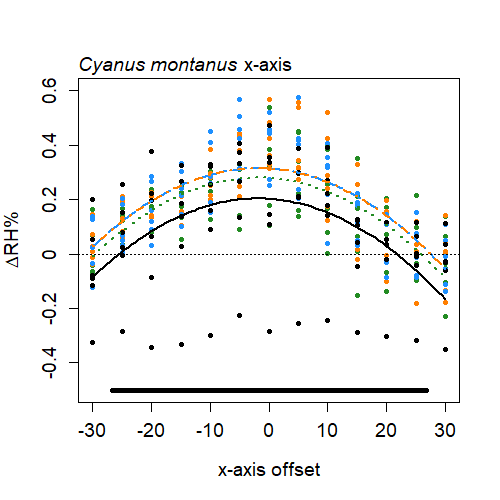

Supplement: SUPPLEMENTARY FILE 3 — A zipped file containing floral humidity structures for the flower species and controls sampled within the study as described in the main text. A word document within the zipped files explains the data set in detail. [file Data_Sheet_3.zip › Floral humidity graphs/Flowers/Cyanus montanus X axis.png]

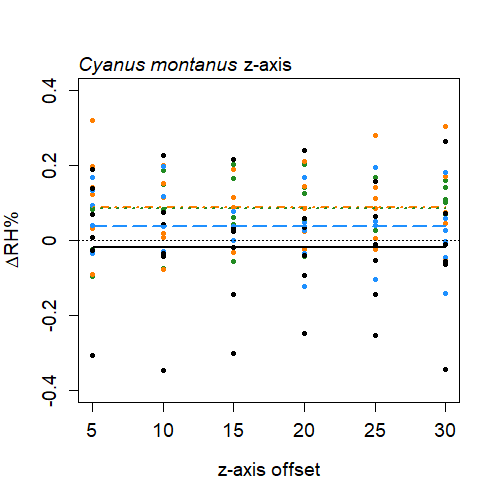

Supplement: SUPPLEMENTARY FILE 3 — A zipped file containing floral humidity structures for the flower species and controls sampled within the study as described in the main text. A word document within the zipped files explains the data set in detail. [file Data_Sheet_3.zip › Floral humidity graphs/Flowers/Cyanus montanus Z axis.png]

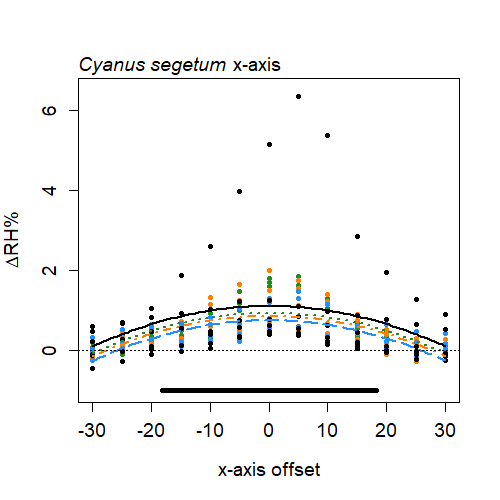

Supplement: SUPPLEMENTARY FILE 3 — A zipped file containing floral humidity structures for the flower species and controls sampled within the study as described in the main text. A word document within the zipped files explains the data set in detail. [file Data_Sheet_3.zip › Floral humidity graphs/Flowers/Cyanus segetum X axis.png]

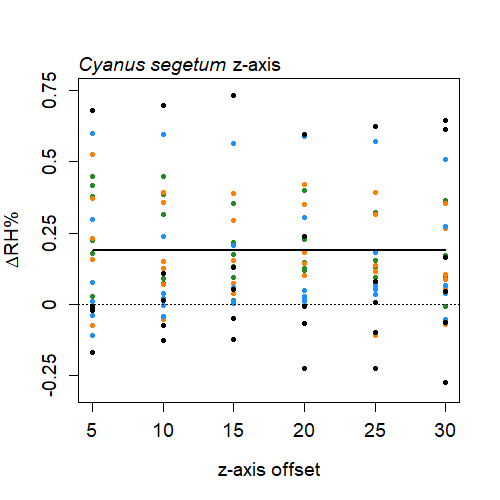

Supplement: SUPPLEMENTARY FILE 3 — A zipped file containing floral humidity structures for the flower species and controls sampled within the study as described in the main text. A word document within the zipped files explains the data set in detail. [file Data_Sheet_3.zip › Floral humidity graphs/Flowers/Cyanus segetum Z axis.png]

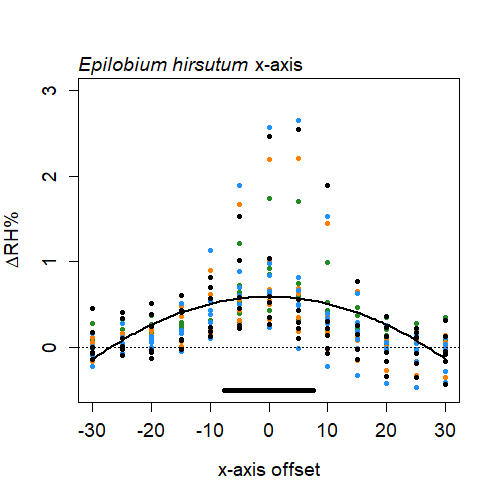

Supplement: SUPPLEMENTARY FILE 3 — A zipped file containing floral humidity structures for the flower species and controls sampled within the study as described in the main text. A word document within the zipped files explains the data set in detail. [file Data_Sheet_3.zip › Floral humidity graphs/Flowers/Epilobium hirsutum X axis.png]

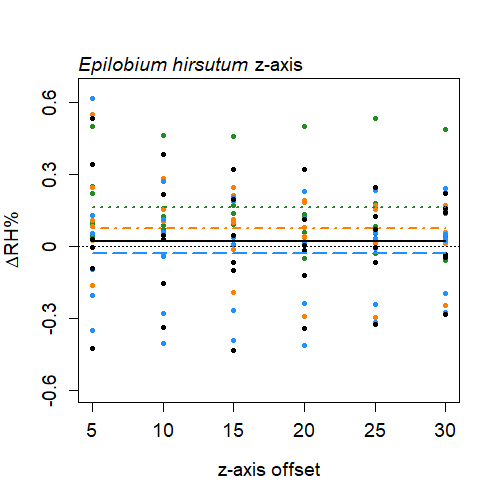

Supplement: SUPPLEMENTARY FILE 3 — A zipped file containing floral humidity structures for the flower species and controls sampled within the study as described in the main text. A word document within the zipped files explains the data set in detail. [file Data_Sheet_3.zip › Floral humidity graphs/Flowers/Epilobium hirsutum Z axis.png]

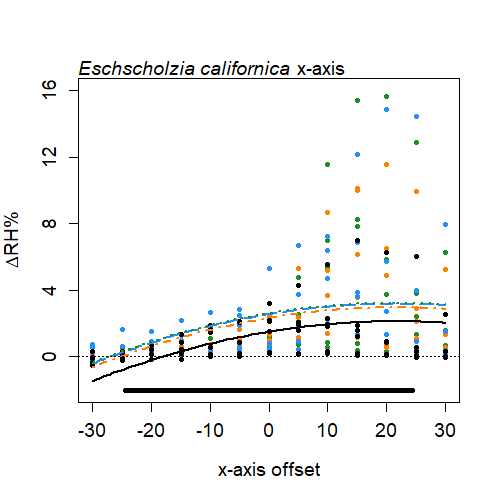

Supplement: SUPPLEMENTARY FILE 3 — A zipped file containing floral humidity structures for the flower species and controls sampled within the study as described in the main text. A word document within the zipped files explains the data set in detail. [file Data_Sheet_3.zip › Floral humidity graphs/Flowers/Eschscholzia californica X axis.png]

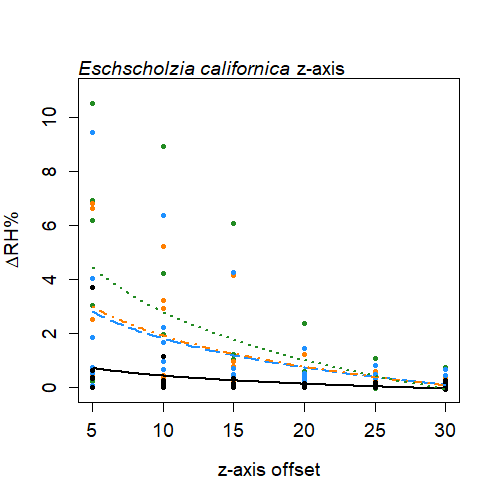

Supplement: SUPPLEMENTARY FILE 3 — A zipped file containing floral humidity structures for the flower species and controls sampled within the study as described in the main text. A word document within the zipped files explains the data set in detail. [file Data_Sheet_3.zip › Floral humidity graphs/Flowers/Eschscholzia californica Z axis.png]

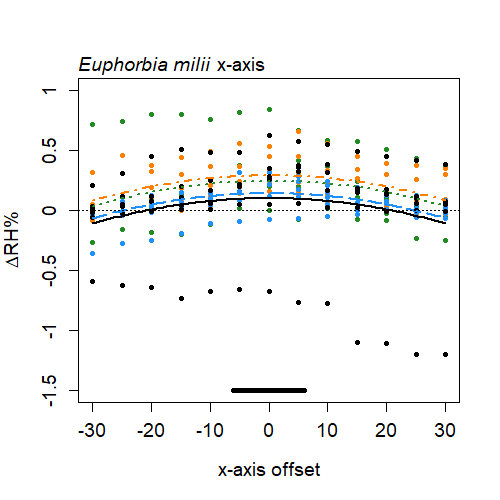

Supplement: SUPPLEMENTARY FILE 3 — A zipped file containing floral humidity structures for the flower species and controls sampled within the study as described in the main text. A word document within the zipped files explains the data set in detail. [file Data_Sheet_3.zip › Floral humidity graphs/Flowers/Euphorbia milii X axis.png]

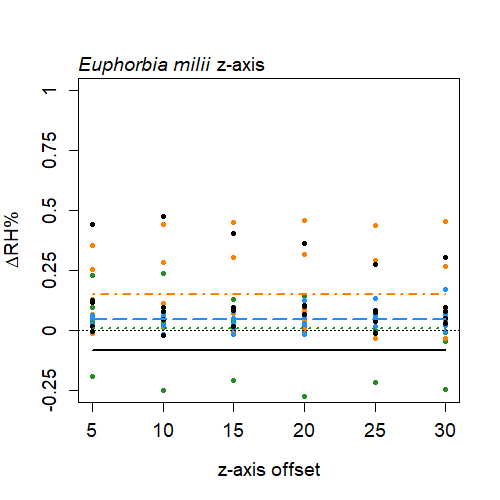

Supplement: SUPPLEMENTARY FILE 3 — A zipped file containing floral humidity structures for the flower species and controls sampled within the study as described in the main text. A word document within the zipped files explains the data set in detail. [file Data_Sheet_3.zip › Floral humidity graphs/Flowers/Euphorbia milii Z azis.png]

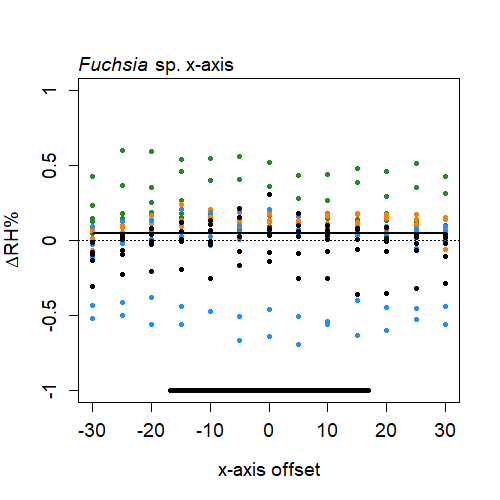

Supplement: SUPPLEMENTARY FILE 3 — A zipped file containing floral humidity structures for the flower species and controls sampled within the study as described in the main text. A word document within the zipped files explains the data set in detail. [file Data_Sheet_3.zip › Floral humidity graphs/Flowers/Fuchsia X axis.png]

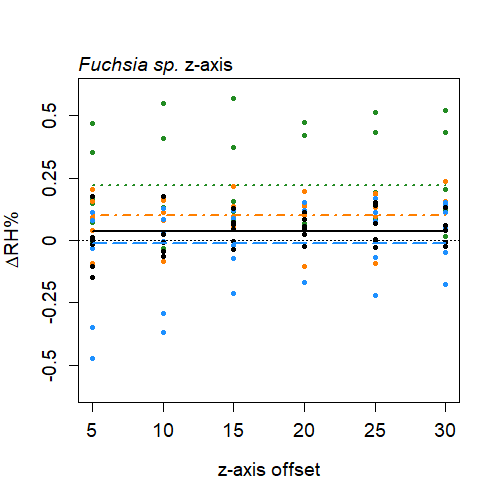

Supplement: SUPPLEMENTARY FILE 3 — A zipped file containing floral humidity structures for the flower species and controls sampled within the study as described in the main text. A word document within the zipped files explains the data set in detail. [file Data_Sheet_3.zip › Floral humidity graphs/Flowers/Fuchsia Z axis.png]

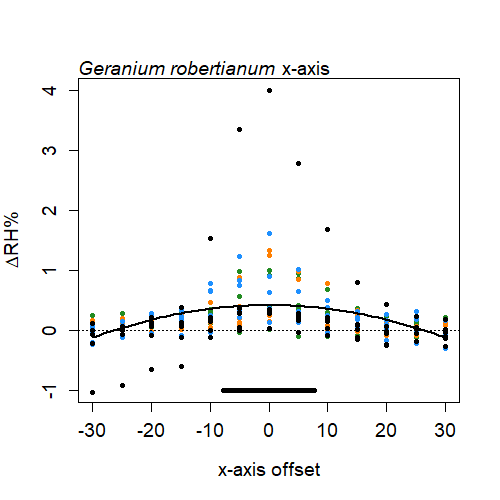

Supplement: SUPPLEMENTARY FILE 3 — A zipped file containing floral humidity structures for the flower species and controls sampled within the study as described in the main text. A word document within the zipped files explains the data set in detail. [file Data_Sheet_3.zip › Floral humidity graphs/Flowers/Geranium robertianum X axis.png]

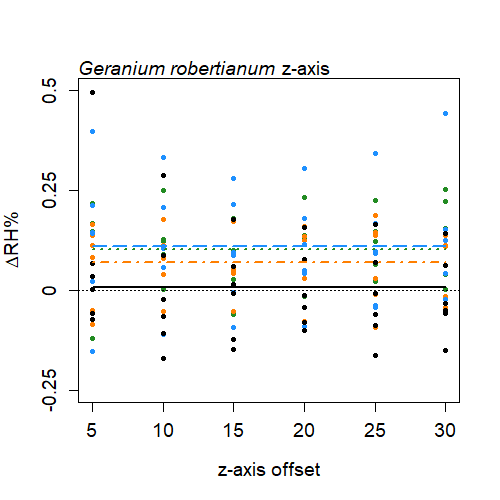

Supplement: SUPPLEMENTARY FILE 3 — A zipped file containing floral humidity structures for the flower species and controls sampled within the study as described in the main text. A word document within the zipped files explains the data set in detail. [file Data_Sheet_3.zip › Floral humidity graphs/Flowers/Geranium robertianum Z axis.png]

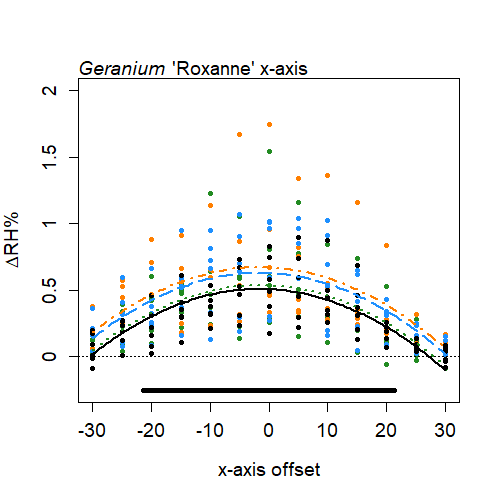

Supplement: SUPPLEMENTARY FILE 3 — A zipped file containing floral humidity structures for the flower species and controls sampled within the study as described in the main text. A word document within the zipped files explains the data set in detail. [file Data_Sheet_3.zip › Floral humidity graphs/Flowers/Geranium 'Roxanne' X axis.png]

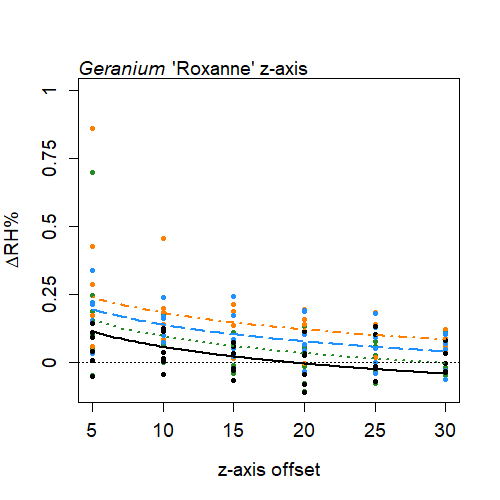

Supplement: SUPPLEMENTARY FILE 3 — A zipped file containing floral humidity structures for the flower species and controls sampled within the study as described in the main text. A word document within the zipped files explains the data set in detail. [file Data_Sheet_3.zip › Floral humidity graphs/Flowers/Geranium 'Roxanne' Z axis.png]

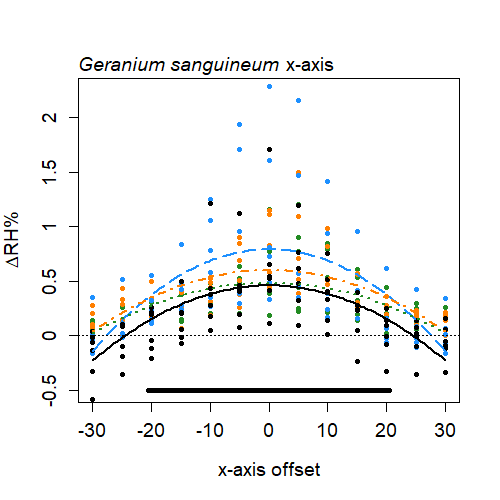

Supplement: SUPPLEMENTARY FILE 3 — A zipped file containing floral humidity structures for the flower species and controls sampled within the study as described in the main text. A word document within the zipped files explains the data set in detail. [file Data_Sheet_3.zip › Floral humidity graphs/Flowers/Geranium sanguineum X axis.png]

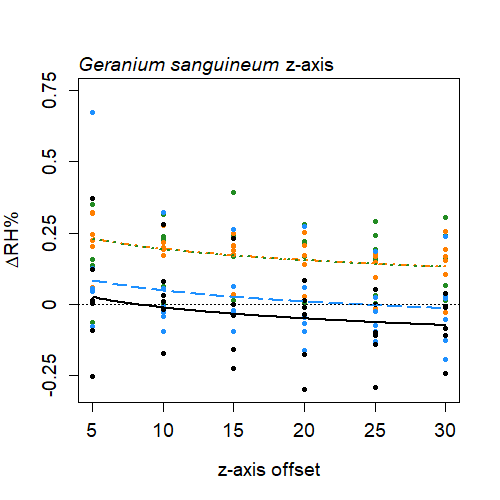

Supplement: SUPPLEMENTARY FILE 3 — A zipped file containing floral humidity structures for the flower species and controls sampled within the study as described in the main text. A word document within the zipped files explains the data set in detail. [file Data_Sheet_3.zip › Floral humidity graphs/Flowers/Geranium sanguineum Z axis.png]

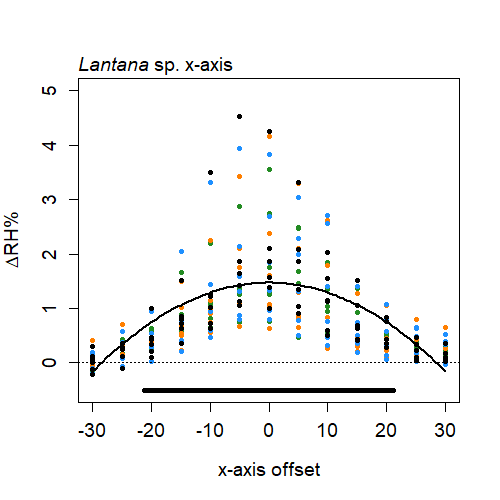

Supplement: SUPPLEMENTARY FILE 3 — A zipped file containing floral humidity structures for the flower species and controls sampled within the study as described in the main text. A word document within the zipped files explains the data set in detail. [file Data_Sheet_3.zip › Floral humidity graphs/Flowers/Lantana X axis.png]

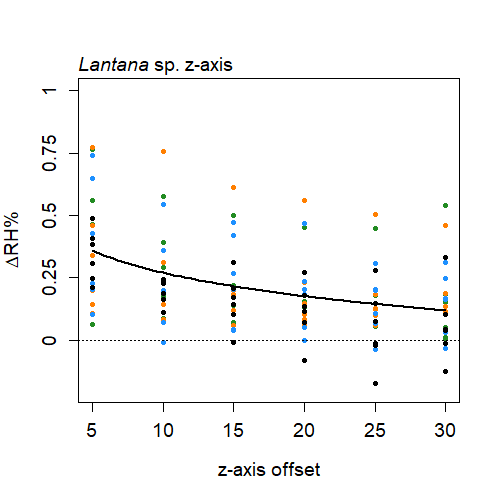

Supplement: SUPPLEMENTARY FILE 3 — A zipped file containing floral humidity structures for the flower species and controls sampled within the study as described in the main text. A word document within the zipped files explains the data set in detail. [file Data_Sheet_3.zip › Floral humidity graphs/Flowers/Lantana Z axis.png]

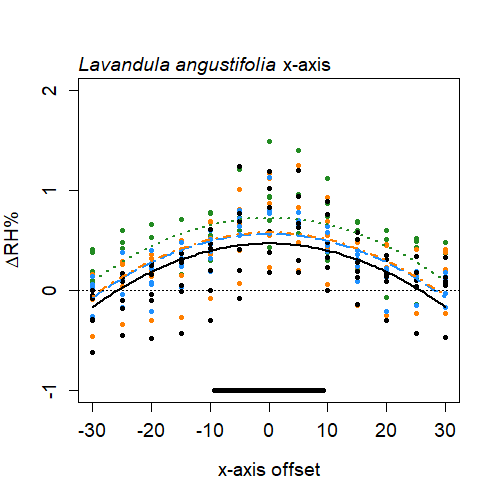

Supplement: SUPPLEMENTARY FILE 3 — A zipped file containing floral humidity structures for the flower species and controls sampled within the study as described in the main text. A word document within the zipped files explains the data set in detail. [file Data_Sheet_3.zip › Floral humidity graphs/Flowers/Lavandula angustifolia X axis.png]

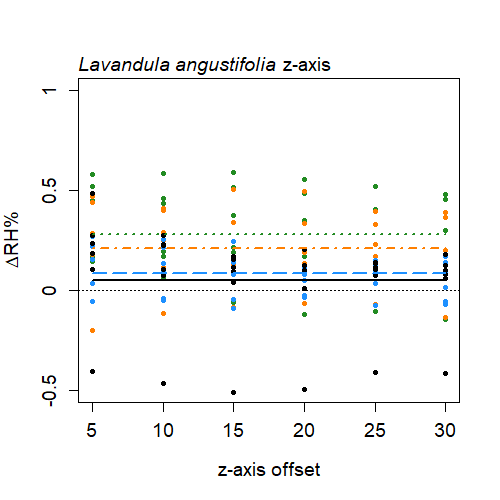

Supplement: SUPPLEMENTARY FILE 3 — A zipped file containing floral humidity structures for the flower species and controls sampled within the study as described in the main text. A word document within the zipped files explains the data set in detail. [file Data_Sheet_3.zip › Floral humidity graphs/Flowers/Lavandula angustifolia Z axis.png]

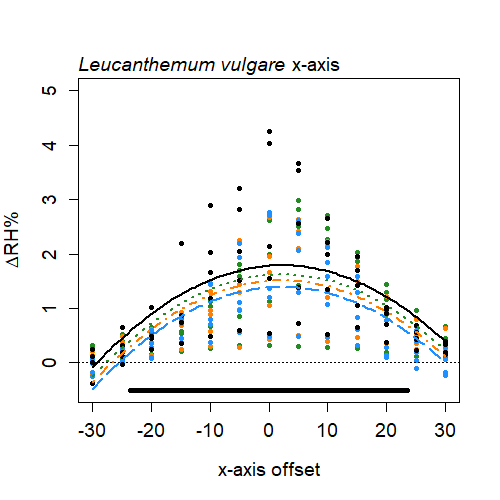

Supplement: SUPPLEMENTARY FILE 3 — A zipped file containing floral humidity structures for the flower species and controls sampled within the study as described in the main text. A word document within the zipped files explains the data set in detail. [file Data_Sheet_3.zip › Floral humidity graphs/Flowers/Leucanthemum vulgare X axis.png]

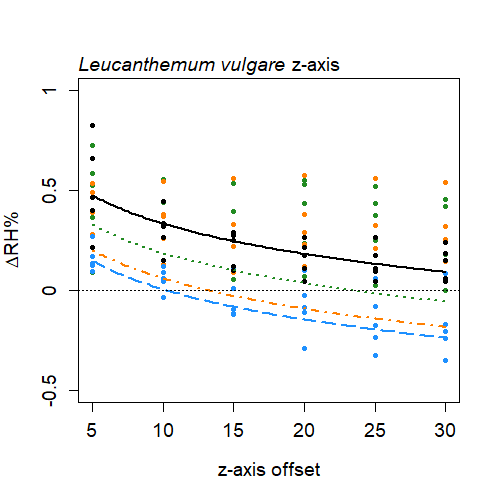

Supplement: SUPPLEMENTARY FILE 3 — A zipped file containing floral humidity structures for the flower species and controls sampled within the study as described in the main text. A word document within the zipped files explains the data set in detail. [file Data_Sheet_3.zip › Floral humidity graphs/Flowers/Leucanthemum vulgare Z axis.png]

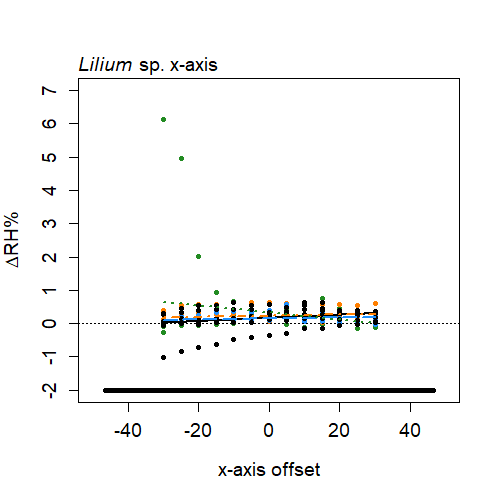

Supplement: SUPPLEMENTARY FILE 3 — A zipped file containing floral humidity structures for the flower species and controls sampled within the study as described in the main text. A word document within the zipped files explains the data set in detail. [file Data_Sheet_3.zip › Floral humidity graphs/Flowers/Lilium X axis.png]

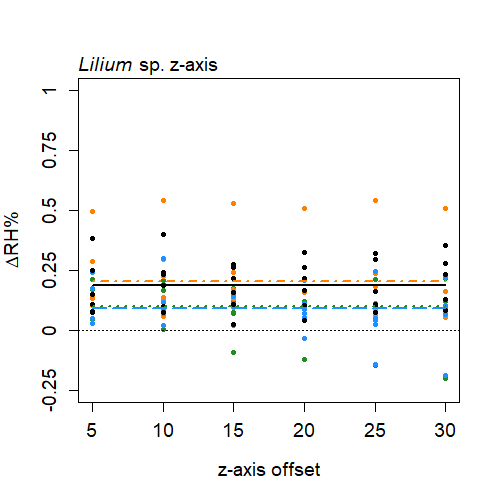

Supplement: SUPPLEMENTARY FILE 3 — A zipped file containing floral humidity structures for the flower species and controls sampled within the study as described in the main text. A word document within the zipped files explains the data set in detail. [file Data_Sheet_3.zip › Floral humidity graphs/Flowers/Lilium Z axis.png]

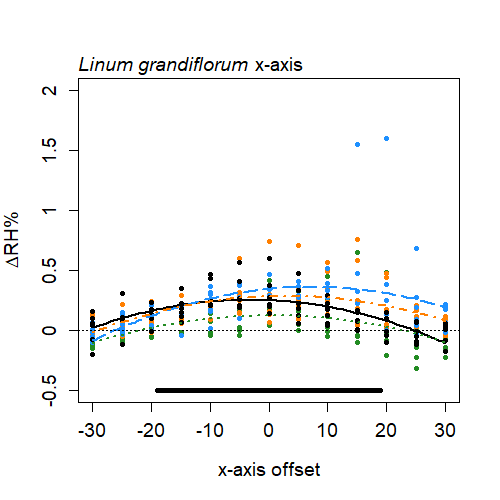

Supplement: SUPPLEMENTARY FILE 3 — A zipped file containing floral humidity structures for the flower species and controls sampled within the study as described in the main text. A word document within the zipped files explains the data set in detail. [file Data_Sheet_3.zip › Floral humidity graphs/Flowers/Linum grandiflorum X axis.png]

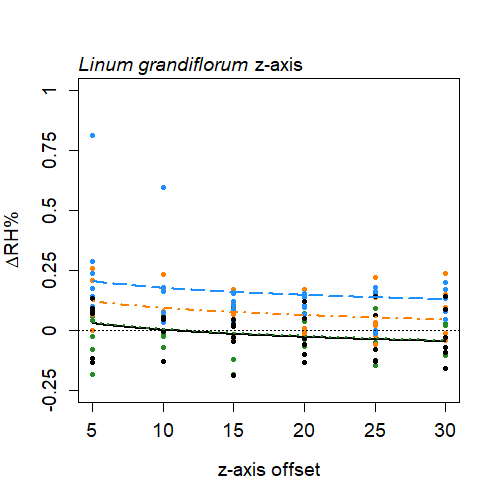

Supplement: SUPPLEMENTARY FILE 3 — A zipped file containing floral humidity structures for the flower species and controls sampled within the study as described in the main text. A word document within the zipped files explains the data set in detail. [file Data_Sheet_3.zip › Floral humidity graphs/Flowers/Linum grandiflorum Z axis.png]

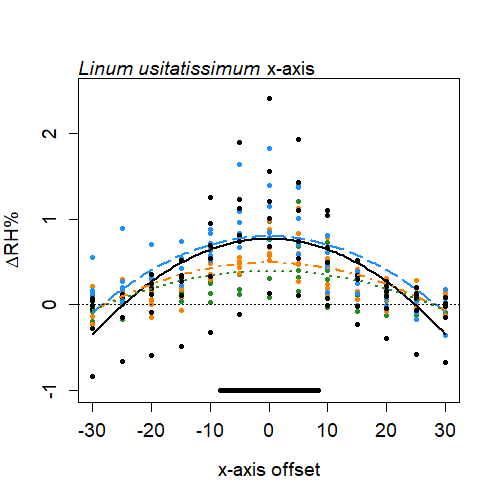

Supplement: SUPPLEMENTARY FILE 3 — A zipped file containing floral humidity structures for the flower species and controls sampled within the study as described in the main text. A word document within the zipped files explains the data set in detail. [file Data_Sheet_3.zip › Floral humidity graphs/Flowers/Linum usitatissimum X axis.png]

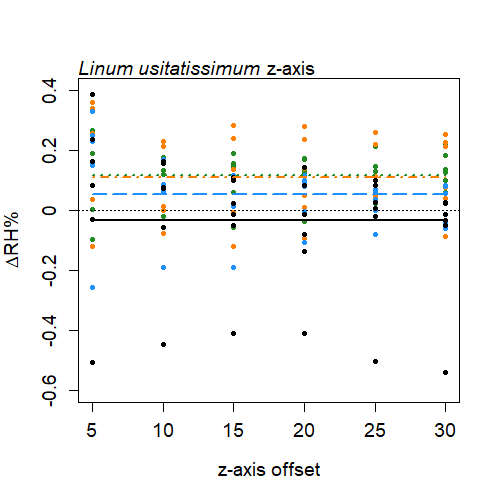

Supplement: SUPPLEMENTARY FILE 3 — A zipped file containing floral humidity structures for the flower species and controls sampled within the study as described in the main text. A word document within the zipped files explains the data set in detail. [file Data_Sheet_3.zip › Floral humidity graphs/Flowers/Linum usitatissimum Z axis.png]

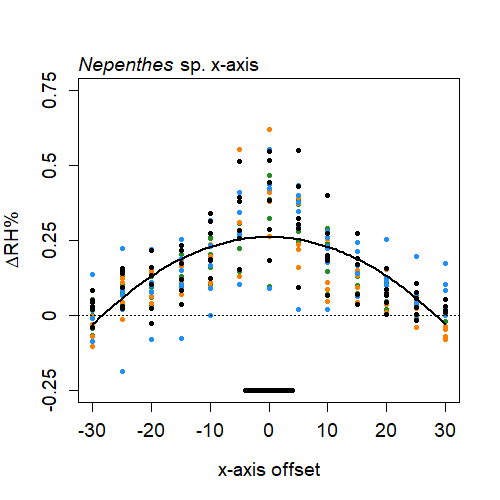

Supplement: SUPPLEMENTARY FILE 3 — A zipped file containing floral humidity structures for the flower species and controls sampled within the study as described in the main text. A word document within the zipped files explains the data set in detail. [file Data_Sheet_3.zip › Floral humidity graphs/Flowers/Nepenthes X axis.png]

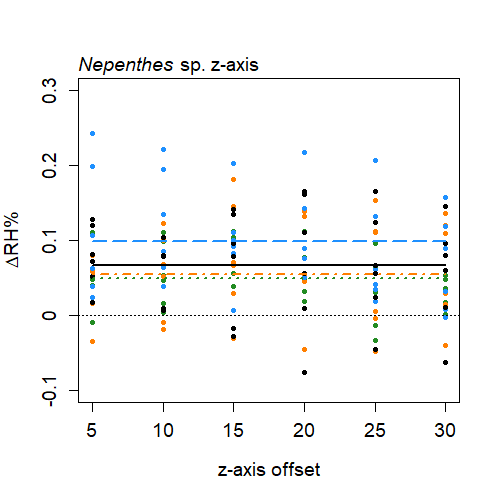

Supplement: SUPPLEMENTARY FILE 3 — A zipped file containing floral humidity structures for the flower species and controls sampled within the study as described in the main text. A word document within the zipped files explains the data set in detail. [file Data_Sheet_3.zip › Floral humidity graphs/Flowers/Nepenthes Z axis.png]

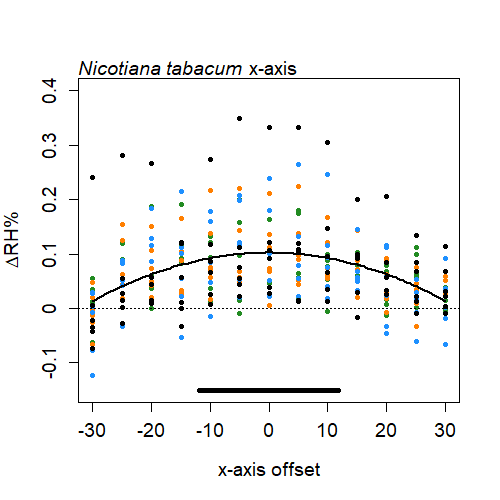

Supplement: SUPPLEMENTARY FILE 3 — A zipped file containing floral humidity structures for the flower species and controls sampled within the study as described in the main text. A word document within the zipped files explains the data set in detail. [file Data_Sheet_3.zip › Floral humidity graphs/Flowers/Nicotiana tabacum X axis.png]

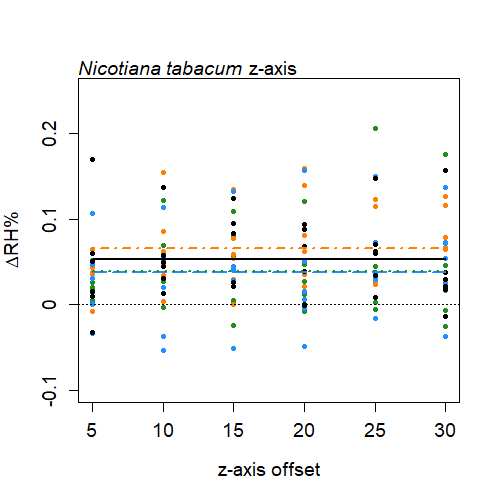

Supplement: SUPPLEMENTARY FILE 3 — A zipped file containing floral humidity structures for the flower species and controls sampled within the study as described in the main text. A word document within the zipped files explains the data set in detail. [file Data_Sheet_3.zip › Floral humidity graphs/Flowers/Nicotiana tabacum Z axis.png]

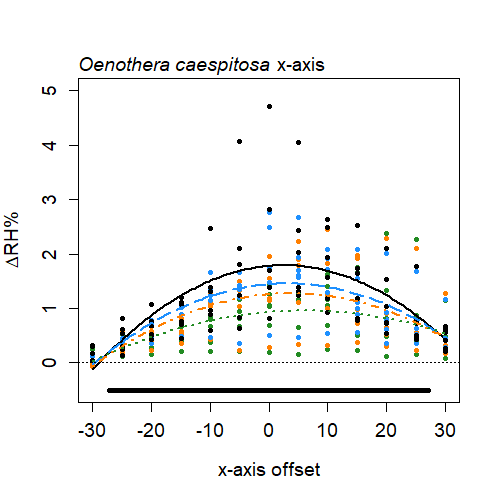

Supplement: SUPPLEMENTARY FILE 3 — A zipped file containing floral humidity structures for the flower species and controls sampled within the study as described in the main text. A word document within the zipped files explains the data set in detail. [file Data_Sheet_3.zip › Floral humidity graphs/Flowers/Oenothera caespitosa X axis.png]

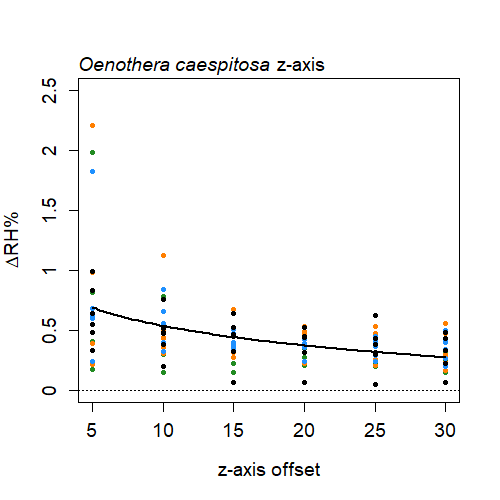

Supplement: SUPPLEMENTARY FILE 3 — A zipped file containing floral humidity structures for the flower species and controls sampled within the study as described in the main text. A word document within the zipped files explains the data set in detail. [file Data_Sheet_3.zip › Floral humidity graphs/Flowers/Oenothera caespitosa Z axis.png]

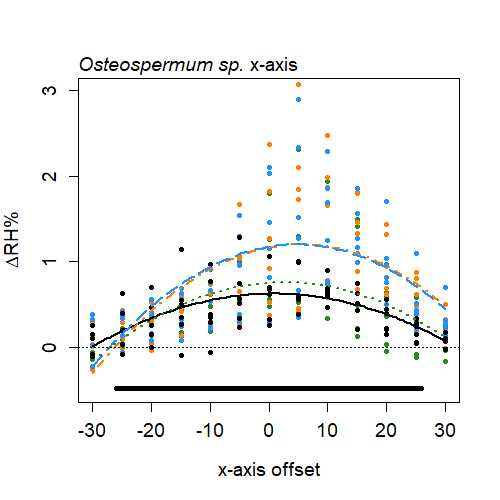

Supplement: SUPPLEMENTARY FILE 3 — A zipped file containing floral humidity structures for the flower species and controls sampled within the study as described in the main text. A word document within the zipped files explains the data set in detail. [file Data_Sheet_3.zip › Floral humidity graphs/Flowers/Osteospermum X axis.png]

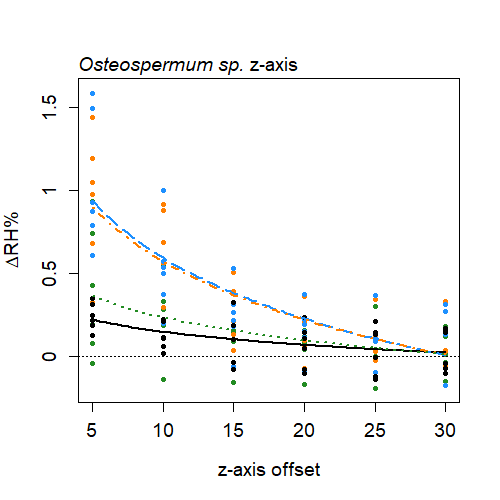

Supplement: SUPPLEMENTARY FILE 3 — A zipped file containing floral humidity structures for the flower species and controls sampled within the study as described in the main text. A word document within the zipped files explains the data set in detail. [file Data_Sheet_3.zip › Floral humidity graphs/Flowers/Osteospermum Z axis.png]

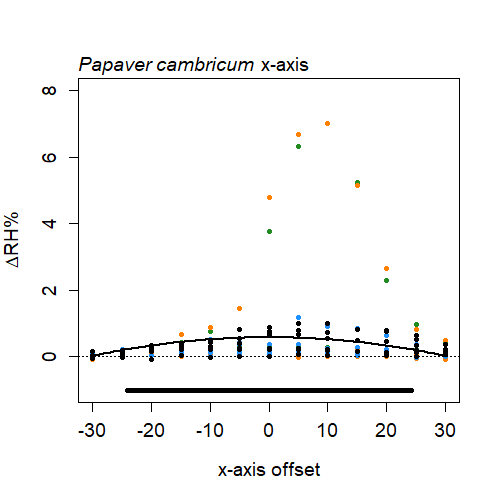

Supplement: SUPPLEMENTARY FILE 3 — A zipped file containing floral humidity structures for the flower species and controls sampled within the study as described in the main text. A word document within the zipped files explains the data set in detail. [file Data_Sheet_3.zip › Floral humidity graphs/Flowers/Papaver cambricum X axis.png]

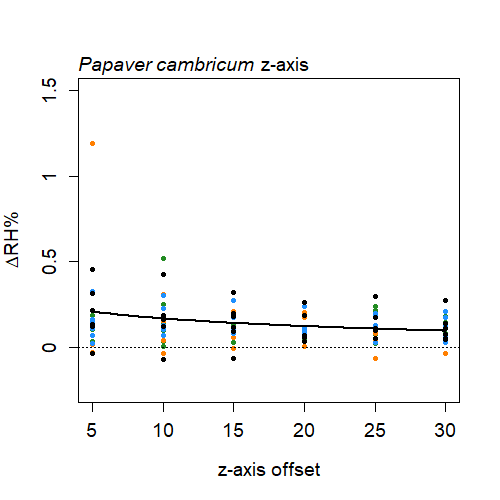

Supplement: SUPPLEMENTARY FILE 3 — A zipped file containing floral humidity structures for the flower species and controls sampled within the study as described in the main text. A word document within the zipped files explains the data set in detail. [file Data_Sheet_3.zip › Floral humidity graphs/Flowers/Papaver cambricum Z axis.png]

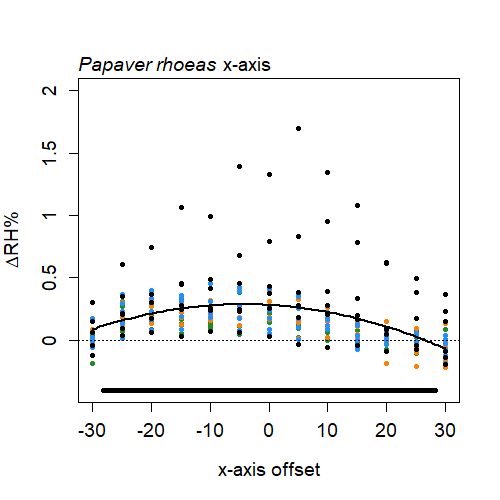

Supplement: SUPPLEMENTARY FILE 3 — A zipped file containing floral humidity structures for the flower species and controls sampled within the study as described in the main text. A word document within the zipped files explains the data set in detail. [file Data_Sheet_3.zip › Floral humidity graphs/Flowers/Papaver rhoeas X axis.png]

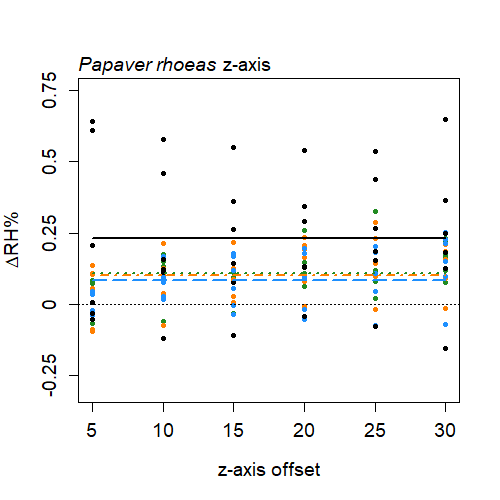

Supplement: SUPPLEMENTARY FILE 3 — A zipped file containing floral humidity structures for the flower species and controls sampled within the study as described in the main text. A word document within the zipped files explains the data set in detail. [file Data_Sheet_3.zip › Floral humidity graphs/Flowers/Papaver rhoeas Z axis.png]

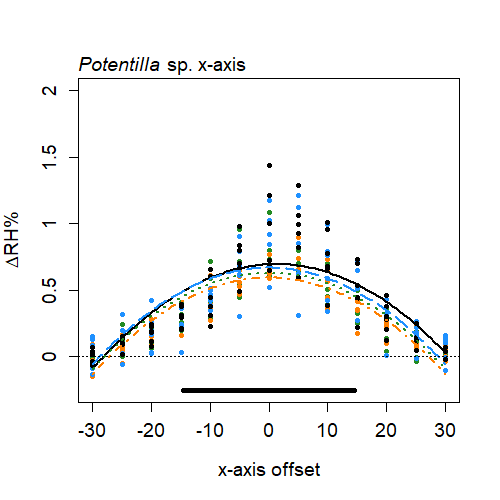

Supplement: SUPPLEMENTARY FILE 3 — A zipped file containing floral humidity structures for the flower species and controls sampled within the study as described in the main text. A word document within the zipped files explains the data set in detail. [file Data_Sheet_3.zip › Floral humidity graphs/Flowers/Potentilla X axis.png]

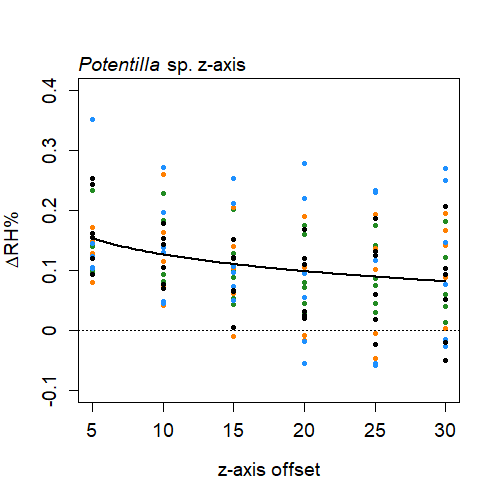

Supplement: SUPPLEMENTARY FILE 3 — A zipped file containing floral humidity structures for the flower species and controls sampled within the study as described in the main text. A word document within the zipped files explains the data set in detail. [file Data_Sheet_3.zip › Floral humidity graphs/Flowers/Potentilla Z axis.png]

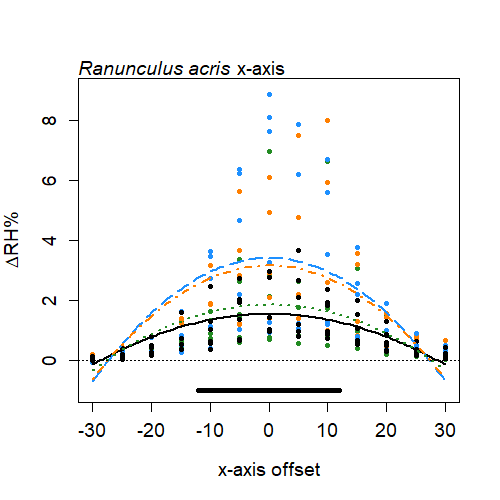

Supplement: SUPPLEMENTARY FILE 3 — A zipped file containing floral humidity structures for the flower species and controls sampled within the study as described in the main text. A word document within the zipped files explains the data set in detail. [file Data_Sheet_3.zip › Floral humidity graphs/Flowers/Ranunculus acris X axis.png]

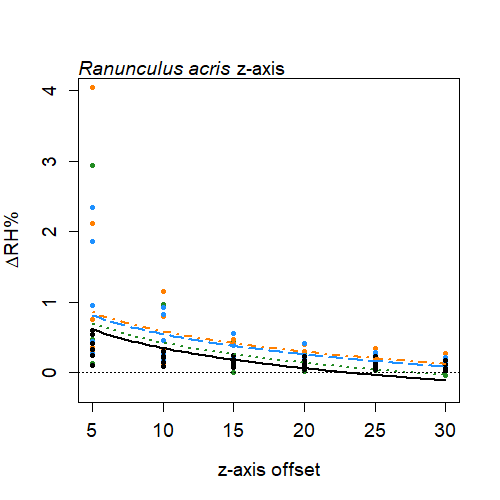

Supplement: SUPPLEMENTARY FILE 3 — A zipped file containing floral humidity structures for the flower species and controls sampled within the study as described in the main text. A word document within the zipped files explains the data set in detail. [file Data_Sheet_3.zip › Floral humidity graphs/Flowers/Ranunculus acris Z axis.png]

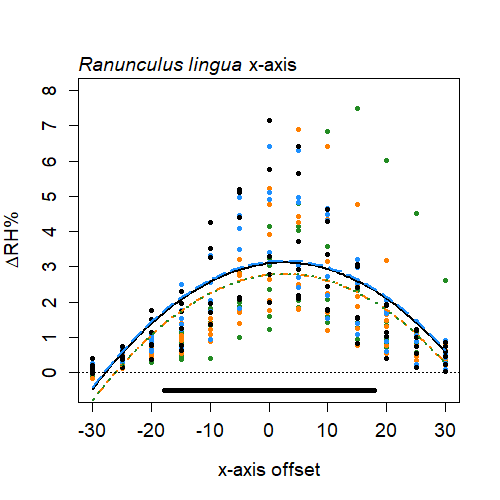

Supplement: SUPPLEMENTARY FILE 3 — A zipped file containing floral humidity structures for the flower species and controls sampled within the study as described in the main text. A word document within the zipped files explains the data set in detail. [file Data_Sheet_3.zip › Floral humidity graphs/Flowers/Ranunculus lingua X axis.png]

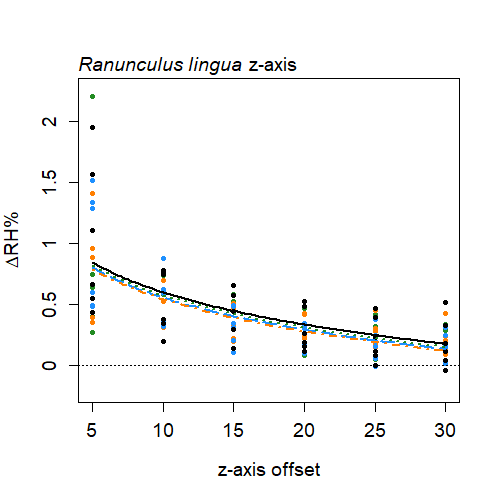

Supplement: SUPPLEMENTARY FILE 3 — A zipped file containing floral humidity structures for the flower species and controls sampled within the study as described in the main text. A word document within the zipped files explains the data set in detail. [file Data_Sheet_3.zip › Floral humidity graphs/Flowers/Ranunculus lingua Z axis.png]

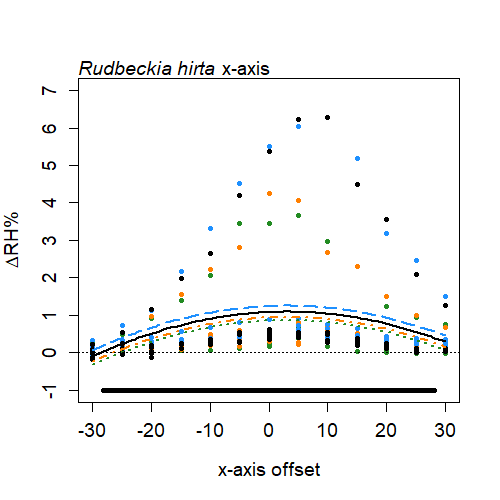

Supplement: SUPPLEMENTARY FILE 3 — A zipped file containing floral humidity structures for the flower species and controls sampled within the study as described in the main text. A word document within the zipped files explains the data set in detail. [file Data_Sheet_3.zip › Floral humidity graphs/Flowers/Rudbeckia hirta X axis.png]

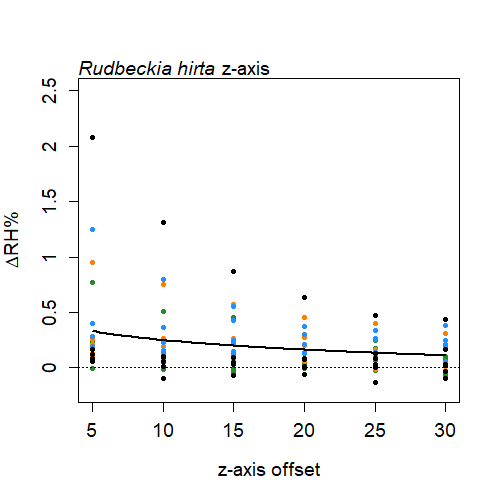

Supplement: SUPPLEMENTARY FILE 3 — A zipped file containing floral humidity structures for the flower species and controls sampled within the study as described in the main text. A word document within the zipped files explains the data set in detail. [file Data_Sheet_3.zip › Floral humidity graphs/Flowers/Rudbeckia hirta Z axis.png]

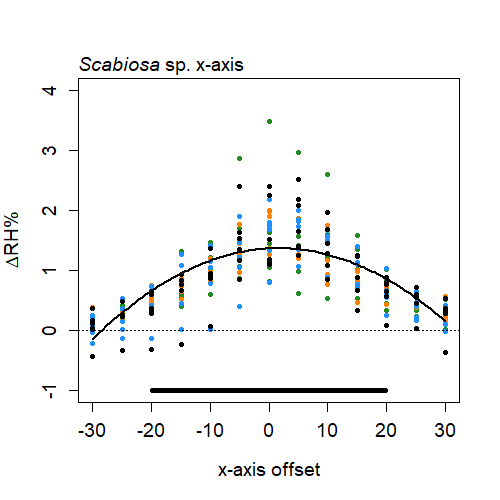

Supplement: SUPPLEMENTARY FILE 3 — A zipped file containing floral humidity structures for the flower species and controls sampled within the study as described in the main text. A word document within the zipped files explains the data set in detail. [file Data_Sheet_3.zip › Floral humidity graphs/Flowers/Scabiosa X axis.png]

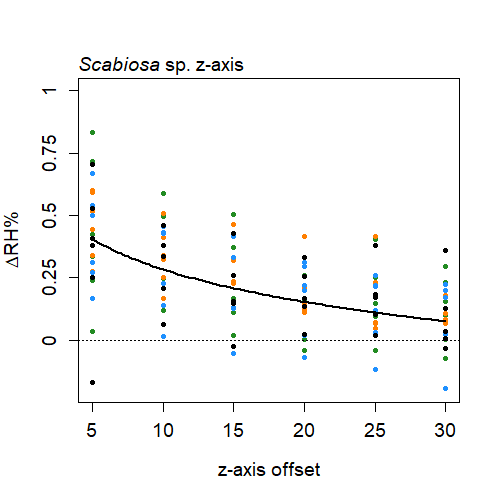

Supplement: SUPPLEMENTARY FILE 3 — A zipped file containing floral humidity structures for the flower species and controls sampled within the study as described in the main text. A word document within the zipped files explains the data set in detail. [file Data_Sheet_3.zip › Floral humidity graphs/Flowers/Scabiosa Z axis.png]

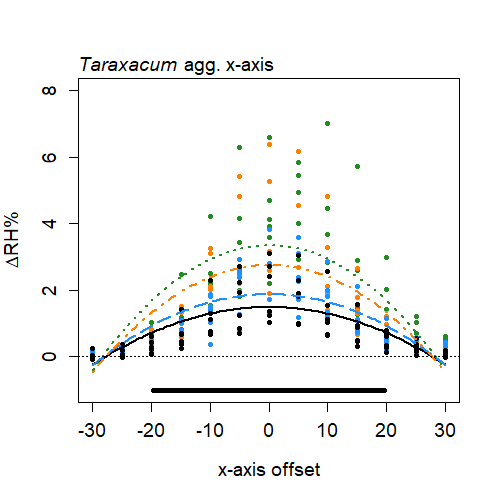

Supplement: SUPPLEMENTARY FILE 3 — A zipped file containing floral humidity structures for the flower species and controls sampled within the study as described in the main text. A word document within the zipped files explains the data set in detail. [file Data_Sheet_3.zip › Floral humidity graphs/Flowers/Taraxacum X axis.png]

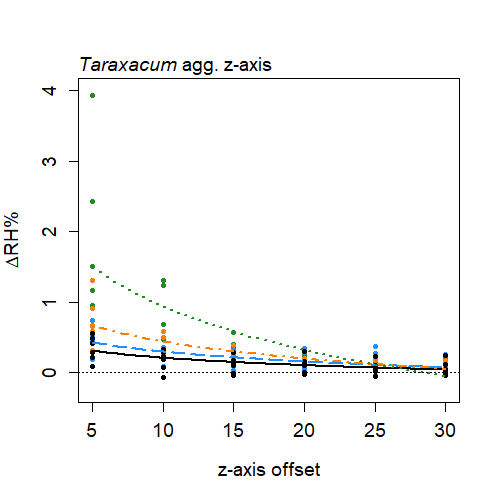

Supplement: SUPPLEMENTARY FILE 3 — A zipped file containing floral humidity structures for the flower species and controls sampled within the study as described in the main text. A word document within the zipped files explains the data set in detail. [file Data_Sheet_3.zip › Floral humidity graphs/Flowers/Taraxacum Z axis.png]

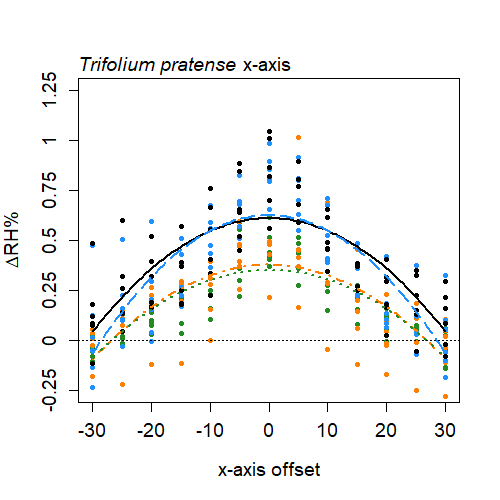

Supplement: SUPPLEMENTARY FILE 3 — A zipped file containing floral humidity structures for the flower species and controls sampled within the study as described in the main text. A word document within the zipped files explains the data set in detail. [file Data_Sheet_3.zip › Floral humidity graphs/Flowers/Trifolium pratense X axis.png]

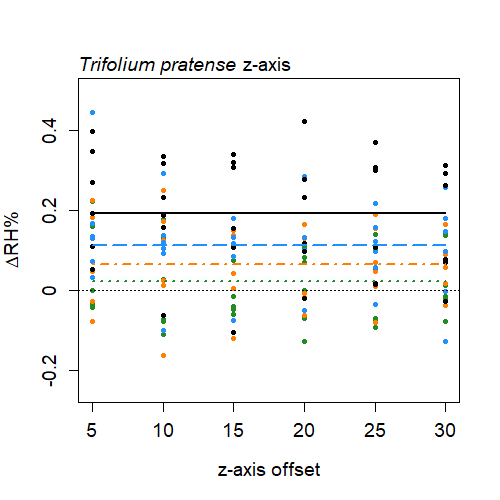

Supplement: SUPPLEMENTARY FILE 3 — A zipped file containing floral humidity structures for the flower species and controls sampled within the study as described in the main text. A word document within the zipped files explains the data set in detail. [file Data_Sheet_3.zip › Floral humidity graphs/Flowers/Trifolium pratense Z axis.png]

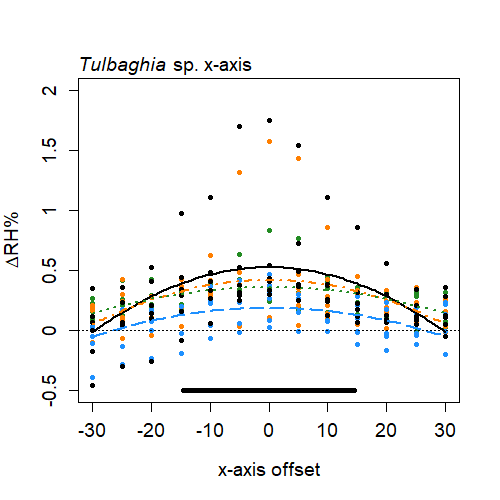

Supplement: SUPPLEMENTARY FILE 3 — A zipped file containing floral humidity structures for the flower species and controls sampled within the study as described in the main text. A word document within the zipped files explains the data set in detail. [file Data_Sheet_3.zip › Floral humidity graphs/Flowers/Tulbaghia X axis.png]

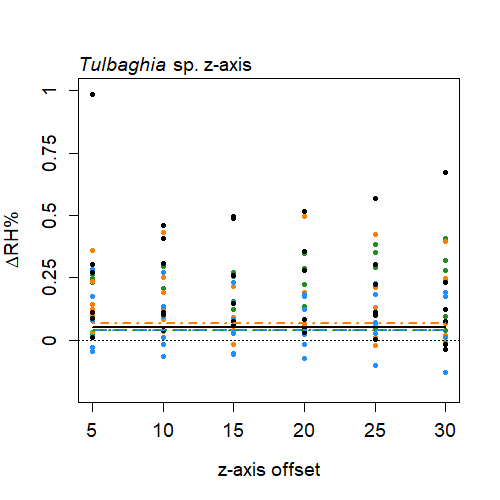

Supplement: SUPPLEMENTARY FILE 3 — A zipped file containing floral humidity structures for the flower species and controls sampled within the study as described in the main text. A word document within the zipped files explains the data set in detail. [file Data_Sheet_3.zip › Floral humidity graphs/Flowers/Tulbaghia Z axis.png]

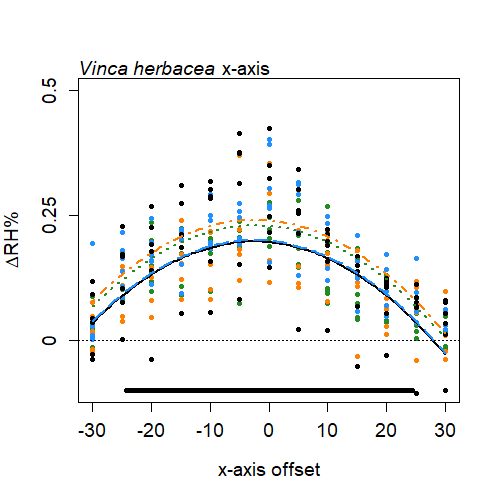

Supplement: SUPPLEMENTARY FILE 3 — A zipped file containing floral humidity structures for the flower species and controls sampled within the study as described in the main text. A word document within the zipped files explains the data set in detail. [file Data_Sheet_3.zip › Floral humidity graphs/Flowers/Vinca herbacea X axis.png]

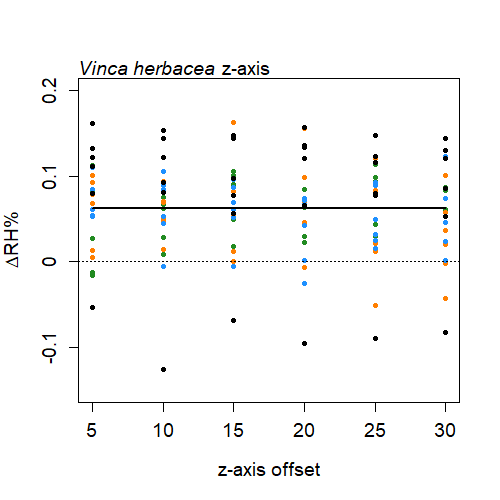

Supplement: SUPPLEMENTARY FILE 3 — A zipped file containing floral humidity structures for the flower species and controls sampled within the study as described in the main text. A word document within the zipped files explains the data set in detail. [file Data_Sheet_3.zip › Floral humidity graphs/Flowers/Vinca herbacea Z axis.png]

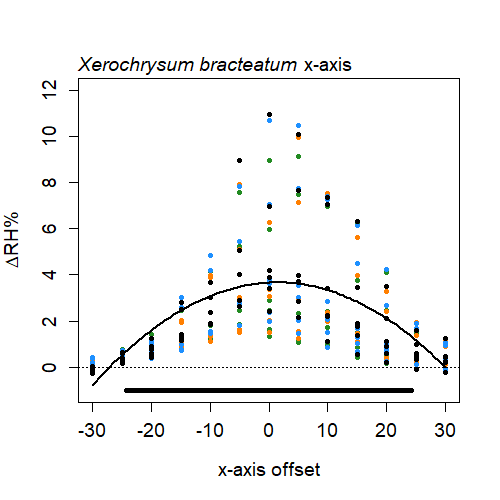

Supplement: SUPPLEMENTARY FILE 3 — A zipped file containing floral humidity structures for the flower species and controls sampled within the study as described in the main text. A word document within the zipped files explains the data set in detail. [file Data_Sheet_3.zip › Floral humidity graphs/Flowers/Xerochrysum bracteatum X axis.png]

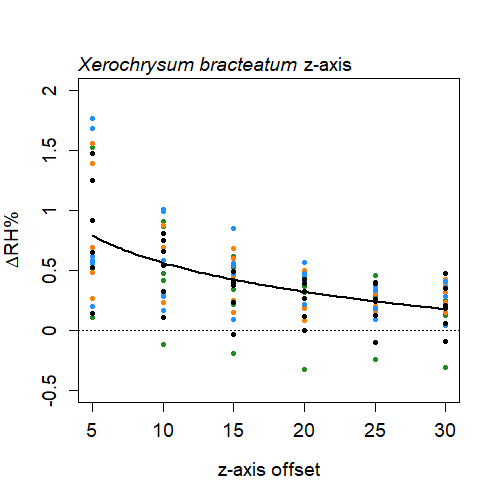

Supplement: SUPPLEMENTARY FILE 3 — A zipped file containing floral humidity structures for the flower species and controls sampled within the study as described in the main text. A word document within the zipped files explains the data set in detail. [file Data_Sheet_3.zip › Floral humidity graphs/Flowers/Xerochrysum bracteatum Z axis.png]

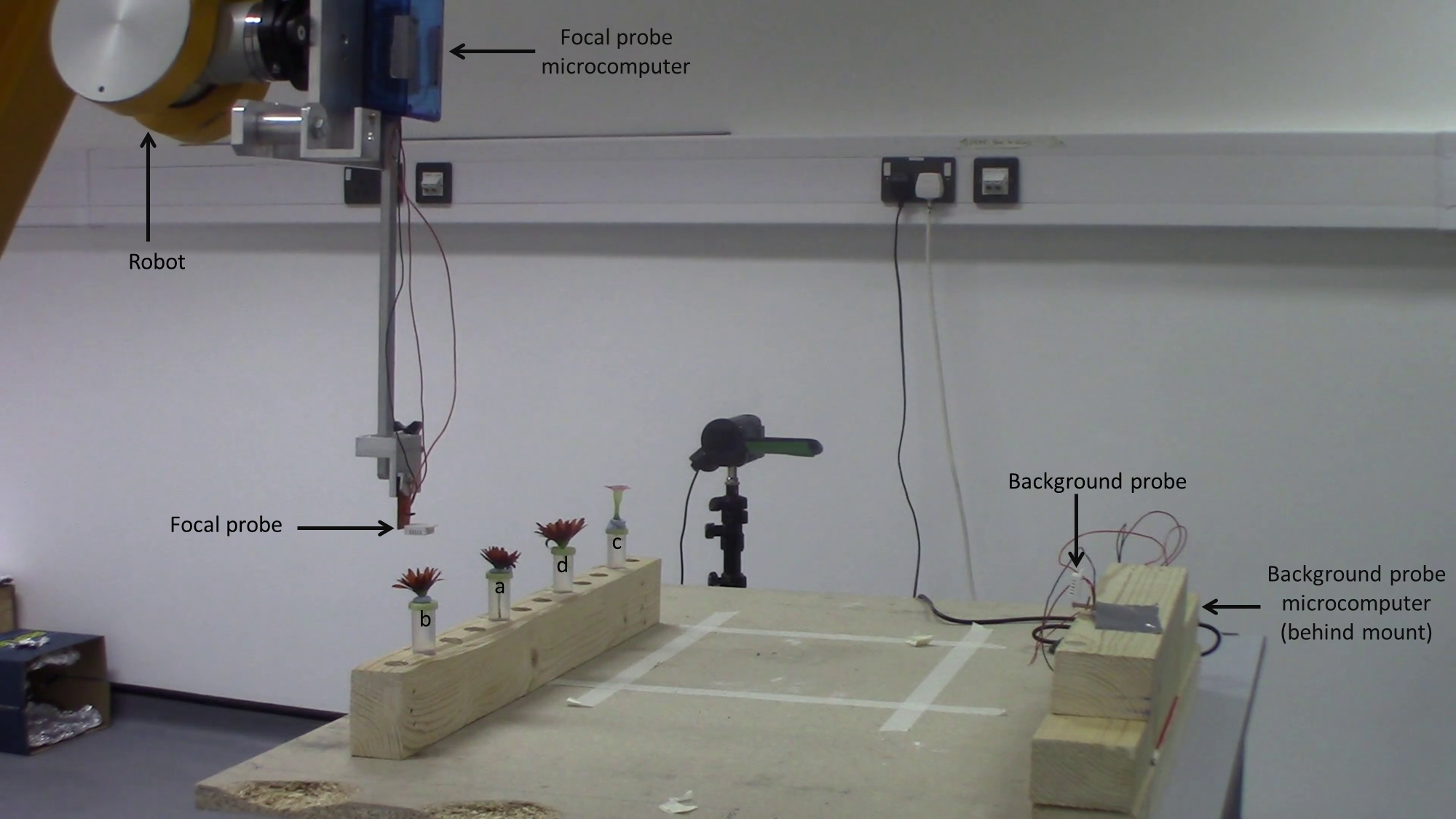

Supplement: SUPPLEMENTARY IMAGE 1 — A labeled frame of Supplementary Video 1 which provides a guide for identifying objects in the video. In addition to the robot, the locations of the focal probe and background probe are indicated, as are the locations of their respective Arduino UNO microcomputers. However, the background probe microcomputer is obscured by the wooden mount supporting the background probe. Flower positions are indicated by letters ‘a,’ ‘b,’ ‘c,’ and ‘d’ as in Figure 3A, indicating the randomly selected sampling order chosen by the robot in this sequence. [file Image_1.TIFF]
